# Development of the Clinical Gestalt Assessment: a visual clinical global impression scale for Proteus syndrome

**DOI:** 10.1186/s13023-022-02325-6

**Published:** 2022-04-23

**Authors:** Christopher A. Ours, Mia B. Hodges, Neal Oden, Julie C. Sapp, Leslie G. Biesecker

**Affiliations:** 1 Center for Precision Health Research, National Human Genome Research Institute, National Institutes of Health, 10 Center Drive 8D47B, Bethesda, MD 20892 USA; 2 The EMMES Corporation, Rockville, MD USA

**Keywords:** Proteus syndrome, Clinician-reported outcome measures, Outcome assessments

## Abstract

**Background:**

Clinical outcome assessments are important tools for measuring the natural history of disease and efficacy of an intervention. The heterogenous phenotype and difficult to quantity features of Proteus syndrome present challenges to measuring clinical outcomes. To address these, we designed a global clinical assessment for Proteus syndrome, a rare mosaic overgrowth disorder. The Clinical Gestalt Assessment (CGA) aims to evaluate change over time in this phenotypically diverse disorder.

**Results:**

We gathered paired serial photographs and radiographs obtained at 12-to-36-month intervals from our natural history study of Proteus syndrome. The chronologic order of each set was blinded and presented to clinicians familiar with overgrowth disorders. They were asked to determine the chronologic order and, based on that response, rate global clinical change using a seven-point scale (Much Worse, Worse, Minimally Worse, No Change, Minimally Improved, Improved, Much Improved). Following a pilot, we tested the inter-rater reliability of the CGA using eight cases rated by eight clinicians. Raters identified the correct chronologic order in 53 of 64 (83%) of responses. There was low inter-rater variance and poor to moderate reliability with an intraclass correlation coefficient of 0.46 (95% CI 0.24–0.75). The overall estimate of global change was Minimally Worse over time, which is an accurate reflection of the natural history of Proteus syndrome.

**Conclusions:**

The CGA is a tool to evaluate clinical change over time in Proteus syndrome and may be a useful adjunct to measure clinical outcomes in prospective therapeutic trials.

**Supplementary information:**

The online version contains supplementary material available at 10.1186/s13023-022-02325-6.

## Background

Clinical outcome assessments are critical to the description of the natural history of disease and measuring the efficacy of an intervention. Establishing fit-for-purpose clinical outcome assessments with sufficient reliability, validity, and responsiveness can be challenging in rare disease research where conditions may be heterogenous and clinical data are limited. This has led to the development of composite endpoints and clinical global scales [1]. Overgrowth disorders, such as *PIK3CA*-related overgrowth spectrum (PROS) and Proteus syndrome, caused by somatic activating genetic variants are rare and heterogeneous in both phenotype and severity. Mosaic disorders such as these have even greater inter-individual variation than constitutional disorders. Individuals with these disorders may experience substantial disease progression, particularly in childhood. These features make them amenable to composite or global outcomes. Measurement of change over time and response to intervention is particularly important as target therapies are being studied in these disorders [2, 3].

Galvanized by a need to assess outcomes in heterogeneous populations, psychiatrists developed the Clinical Global Impression (CGI) scale to enable measurement of the behavioral and cognitive aspects of disease. The CGI was originally designed as a clinician observer-rating following a structured psychiatric interview and distills the clinical impression to a seven-point scale [4]. Versions of this scale have been created to measure illness severity (CGI-S) and improvement (CGI-I) in depression, anxiety, autism spectrum disorder, and schizophrenia [5–7]. The CGI scale has also been adapted for use in neurodevelopmental disorders such as Rett, Prader–Willi, and Angelman syndromes [8–12]. These methods of measuring global change have been successfully applied to interventional clinical trials as primary and secondary outcomes [13, 14].

Proteus syndrome is a rare sporadic and progressive overgrowth disorder caused by a post-zygotic activating variant of *AKT1* [15]. The incidence is difficult to quantify but has been estimated to be 1/1,000,000 births. There are a wide range of manifestations that may affect nearly every tissue. The skin, subcutaneous tissue, bone, and lungs are most commonly affected [16]. Each individual has a unique constellation of overgrowth varying in anatomic site and severity. Despite its phenotypic heterogeneity, a unifying feature of Proteus syndrome is relentless progression and significant mortality, primarily from thromboembolic events, during childhood [17].

There are few published quantitative measurements of Proteus syndrome progression. Leg length discrepancy was reported in a case series of eight individuals who underwent surgical intervention for lower extremity overgrowth [18]. In this study, leg length discrepancy was measured using scanogram radiographs which demonstrated preoperative progression followed by improvement after epiphysiodeses. Another hallmark of the progressive manifestations of Proteus syndrome is the cerebriform connective tissue nevus (CCTN). This lesion is an expansive collagenoma that most commonly occurs on the plantar surface of the foot. Photography of the CCTN has been established as a reliable and reproducible method of measuring the area of this lesion. Shown through repeated measures of serial photography, CCTN lesions increase at a rate of about 5% more involvement of the plantar surface per year during childhood sometimes resulting in complete coverage of the foot [19]. These quantitative metrics to assess progression in Proteus syndrome are important tools but are limited to particular features of the disorder that are not consistently present in all individuals with Proteus syndrome.

Unlike psychiatric and neurodevelopmental disorders, the physical manifestations of Proteus syndrome can be assessed visually through photography and radiography. The commonality of disease progression and ability to visually assess overgrowth led us to develop a visual global assessment adaptation of the CGI. A key motivation for this assessment tool is for use as an outcome measure in clinical trials. Early phase clinical trials and expanded access use of the pan-AKT inhibitor, miransertib (MK-7075), have shown signs of efficacy in Proteus syndrome [2, 20, 21]. Further research of the efficacy of molecularly targeted therapy for Proteus syndrome is needed.

We developed a Clinical Gestalt Assessment (CGA) which presents visual data to clinicians familiar with overgrowth disorders who rate an individual’s global clinical change over time on a seven-point scale. We applied the CGA to the natural history of Proteus syndrome to evaluate the reliability of this method and propose its use in future clinical trials.

## Methods

This study was performed using data collected under human subjects research protocol 94-HG-0132, a longitudinal natural history study of Proteus syndrome that began in 1994 and includes extensive phenotyping of individuals with Proteus syndrome and related disorders. Individuals provided written informed consent to the Natural History of Proteus Syndrome study (94-HG-0132) approved by the National Institutes of Health Institutional Review Board. Written informed consent from parents or guardians was obtained for minors. We reviewed the clinical records from January 1, 2000–December 31, 2019 to identify individuals who met the following criteria: clinical-molecular diagnosis of Proteus syndrome [22], clinical photography and skeletal survey radiographs obtained at a 12 to 36 month interval, and age younger than 18 years at earlier evaluation. A mosaic *AKT1* c.49G>A p.(Glu17Lys) variant in an affected skin biopsy had been confirmed by restriction fragment length polymorphism assay in each included individual. Clinical photography and radiographs were all obtained at the National Institutes of Health Clinical Center (NIHCC).

**Fig. 1:**
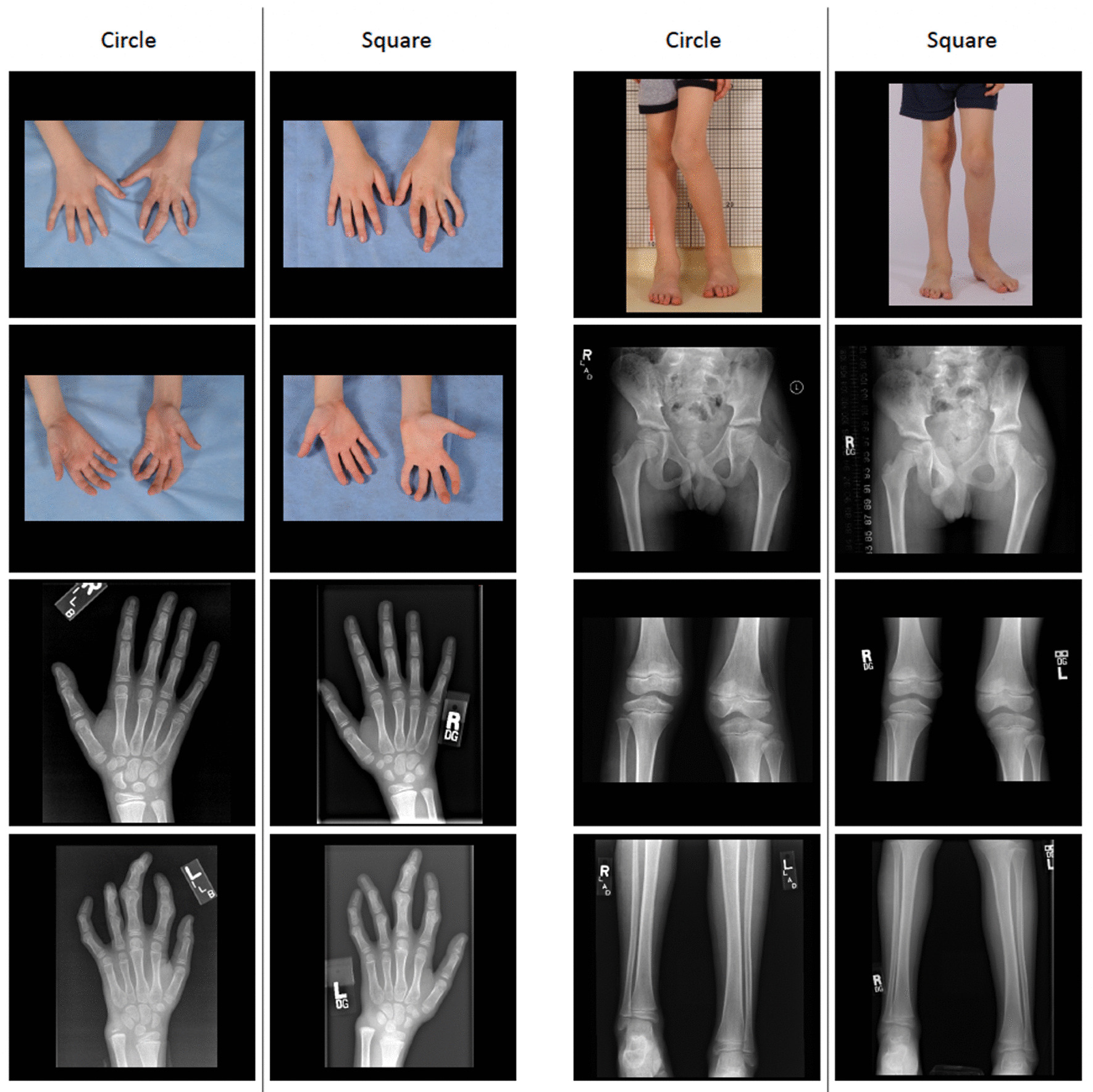
Portion of images presented in Case 1. Square represents the earlier time point

Photographs and radiographs of each individual were organized by timepoint and anatomic region (head and neck, torso, upper extremity, lower extremity, and specific skin lesions). All regions were included regardless of disease involvement. Images were rotated and arranged to provide a vertically oriented side-by-side comparison. In order to blind raters to the chronology of the images and assign nonordered names, the timepoints were randomized to receive a label of “circle” or “square” (Fig. 1). Images with facial features were de-identified to preserve anonymity.

**Fig. 2:**
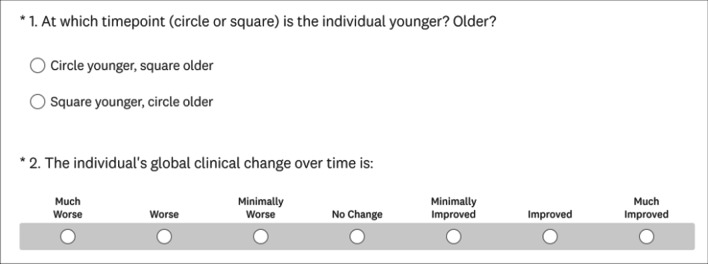
Screenshot of electronic questionnaire presented to raters

The CGA consisted of two questions for each pair of timepoints. First, raters were asked to determine chronology of the timepoints by responding with either “circle younger, square older” or “square younger, circle older”. With chronology established by the rater, they were then asked to rate the individual’s global clinical change on a seven-point scale (Much Worse, Worse, Minimally Worse, No Change, Minimally Improved, Improved, Much Improved). For ratings in which the rater incorrectly assigned chronology the response was reflected across the scale. For example, a rating of Minimally Improved would be converted to Minimally Worse and ratings of No Change remained the same. For analysis, these categories were converted to an integer (Much Worse = 1, Worse = 2, Minimally Worse = 3, No Change = 4, Minimally Improved = 5, Improved = 6, Much Improved = 7) and treated as a continuous variable. The CGA was administered using the SurveyMonkey online platform (Fig. 2) and images were provided in a secure portable document format file (Additional file 1: Fig. S1).

This study was performed in two stages. First, the CGA was piloted by medical genetics physician trainees. This pilot had additional questions including a rating of clinical change by anatomic region and the opportunity to provide feedback on the provided instructions, quality of images, and the rating scale. The scale also used different language for the seven levels (Very Much Worse, Much Worse, Minimally Worse, No Change, Minimally Improved, Much Improved, Very Much Improved). Responses from the pilot were used for power calculations to indicate the number of raters needed to test the inter-rater reliability in the second stage of the study. In response to feedback, the language of the seven-point scale was changed to the aforementioned choices and questions regarding change by anatomic region were eliminated. These changes were intended to increase discrimination at the extremes of the scale and improve ease of administration.

In the second stage of this study, we invited practicing clinical geneticists and pediatric hematologists/oncologists with expertise in overgrowth disorders to complete the revised CGA. These responses were used to calculate the reliability of the CGA and estimate an average CGA rating.

To assess reliability of the CGA, we calculated the intraclass correlation coefficient (ICC). The ICC is an index that describes a measurement tool’s reproducibility. Factors that contribute the variability of a rating measurement such as the CGA include the variability among cases, variability among raters, and random noise. The ICC was calculated by dividing the variability in cases by the total variability (i.e., the sum of variability from cases, raters, and noise). A higher ICC reflected increased reproducibility as the contribution of variability was from cases rather than raters and noise. To quantitate these variabilities data were analyzed as a mixed two-way ANOVA without replication and with random effects for case, rater, and error. Pilot data were used to create a similar ANOVA model which was used for simulation and power calculations. The ICC was calculated as case variance divided by the sum total variances of cases, raters, and error. By convention, this analysis was notated ICC [1, 2] and was interpreted as the reliability when using a single rating. As a post-hoc analysis, we determined the reliability when the average rating of multiple raters is used rather than a single rater, this was notated ICC(2,k) [23]. The proportion of correct chronology responses and confidence interval were estimated using a generalized linear mixed model with normal random effects for case and rater and a logit link. This was performed in SAS Version 9.4. ICC confidence intervals were calculated in R Version 4.0.2 using the RStudio Version 1.0.1073 psych package.

## Results

We identified 41 participants younger than 18 years who had at least one evaluation at the NIHCC that included clinical photography and a skeletal survey. Of these, 12 participants had serial evaluations that included clinical photography and skeletal survey. Eight of these 12 participants met the evaluation interval criteria of 12 to 36 months. The median age at earlier evaluation was 5.4 years (range 1.7–12.3). The median interval between evaluations was 16.8 months (range 12.1–26.4). In the pilot stage, five raters completed responses for three cases. Chronology was correctly assigned in 11/15 (73.3%) responses. The average rating of global change was 3.47 corresponding to Minimally Worse to No Change. Using a two-way ANOVA model, the contributions of variability in the CGA from cases, raters, and noise (i.e., error) were estimated as variances. The case variance estimate was 0.41, rater variance estimate was 0, error variance estimate was 0.83. Based on these data, eight raters were needed to provide 80% power to reject the null hypothesis of an ICC equal to zero.

**Fig. 3:**
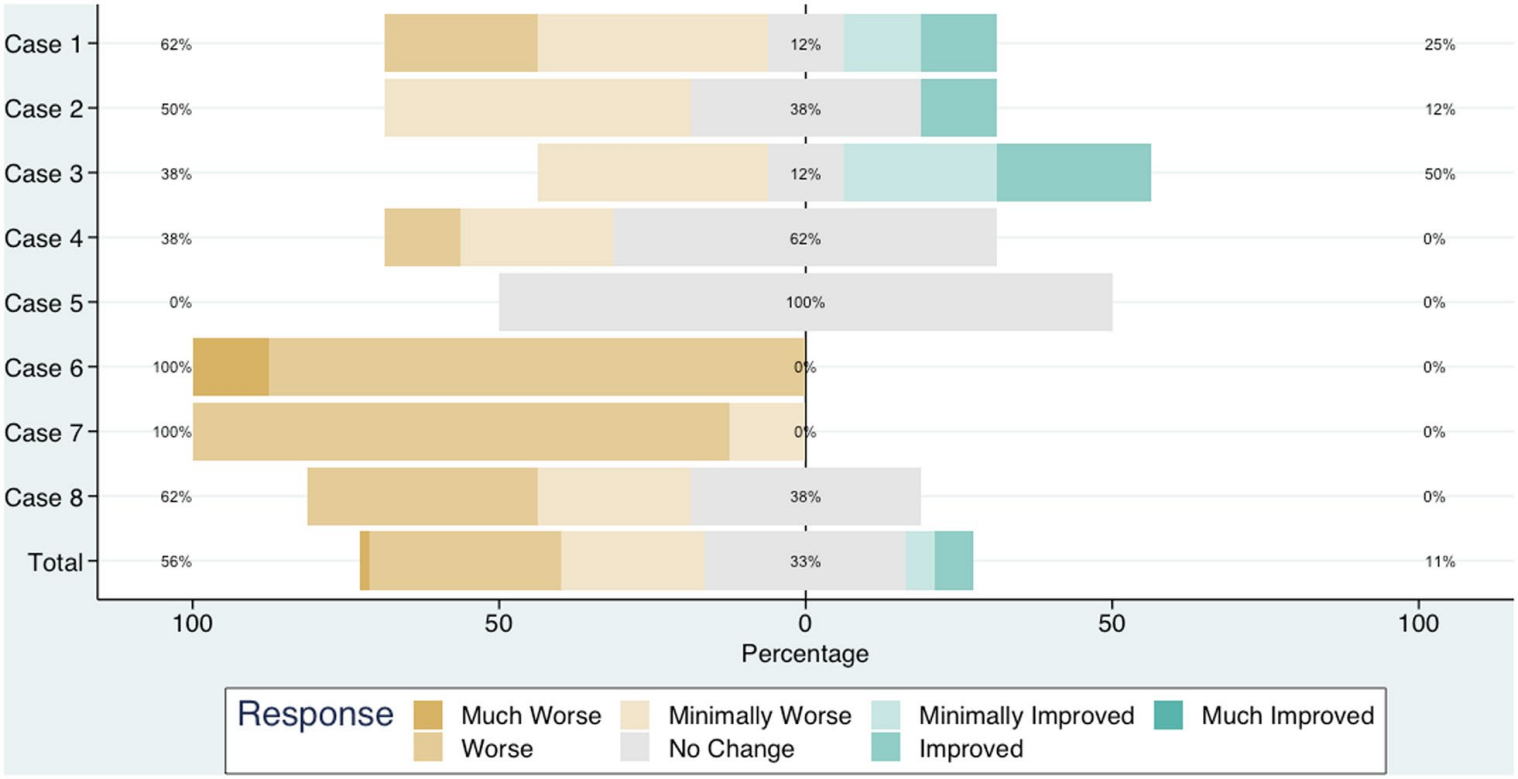
Summary of rater responses by case and in total (N = 8)

In the second stage, eight physician specialists in clinical genetics and pediatric hematology/oncology who are familiar with overgrowth syndromes completed the CGA for the eight cases. Chronology was correctly assigned in 53/64 (82.8% (95% CI 71.3–90.3%)) responses. Incorrect chronology responses predominantly occurred in two cases accounting for 8/11 (73%) of incorrect responses. Case 5 had four incorrect chronology responses but all ratings including those with correct chronology responses were No Change. In Case 6 and Case 7, seven of the eight raters agreed on a rating of Minimally Worse. In five of the eight cases (Cases 1, 2, 6, 7, and 8) at least half of raters indicated worsening over time (Minimally Worse, Worse, or Much Worse). There were no ratings of Much Improved among all responses. In total, the ratings were Much Worse 1/64 (1.6%), Worse 20/64 (31.3%), Minimally Worse 15/64 (23.4%), No Change 21/64 (32.8%), Minimally Improved 3/64 (4.7%), Improved 4/64 (6.3%), Much Improved 0/64 (0%) (Fig. 3, Additional file 2: Table S1).

Using the responses from eight raters of the second stage, a mixed two-way ANOVA model without replication was used to estimate the variance of cases, raters, and residual error. The case variance estimate was 0.67 (*p* = 0.052), rater variance estimate was 0.1 (*p* = 0.44), and error variance estimate was 0.8 (*p* = < 0.0001). The ICC [1, 2] was 0.46 (95% CI 0.24–0.75) and ICC (2,k) was 0.87 (95% CI 0.72–0.96). The average estimated rating was 3.3 (95% CI 2.5–4.0) indicating a rating of Minimally Worse.

## Discussion

Fit-for-purpose clinical assessments are often necessary to evaluate clinical features of rare disease and have particular utility for measuring efficacy in interventional clinical trials. Heterogenous and difficult to quantify features of rare diseases present particular challenges to developing these tools. This study is a proof of principle of a novel method to assess clinical change in individuals with Proteus syndrome using a seven-point scale and visual assessment of clinical photography and plain radiographs. The use of a seven-point scale modeled from the CGI-I scale provides a blinded, semiquantitative assessment of clinical progression. The ICC [1, 2] using a single rating indicated poor to moderate reliability [24]. However, interpreting the CGA using the average rating of multiple raters rather than a single rater improved the reliability. The fixed effect rating estimate of 3.3 (95% CI 2.5–4.0) corresponds to Minimally Worse and is consistent with prior publications describing the progressive natural history of Proteus syndrome [25–27]. This prediction is suggestive of the content validity of the CGA. Raters were able to judge differences over time using this visual method, the CGA was moderately reliable when using the average rating of multiple raters, and ratings derived from cases in a longitudinal natural history study were consistent with the natural history of Proteus syndrome.

The CGA addresses criticisms typical of clinical global scales. Global scales of improvement are often at risk of recall and response shift biases that may occur when the rater unconsciously compares current data to recent clinical encounters rather than the true baseline [28]. Standardization of the clinical examination and having defined components subject to rater clinical judgement are needed for sufficient sensitivity of a global scale [29]. The CGA addresses these by including images from a predefined list of anatomic regions and limits bias in the curation of the images by including images irrespective of disease involvement. Investigator bias is limited through the inclusion of raters not directly involved in the research study. Blinding and simultaneous presentation of data from two timepoints limits recall and response shift biases and allows the CGA to be used in single arm interventional studies when blinding to the intervention is not possible. This is particularly valuable in the study of rare disease where randomization in clinical trials is not always feasible. However, the CGA remains subject to the variability of clinical judgement between raters and may benefit from including more definitive instructions of what constitutes clinical change or by providing additional clinical details beyond the static images.

In the second stage of this study, the CGA’s reliability as measured by the ICC was poor to moderate. The source of this poor reliability was reflective of the lack of complete agreement between raters in five of the eight cases. The theme that Proteus syndrome worsens over time may also have contributed to the poor reliability. A ceiling effect on the ICC can occur when there is lack of variation among cases [30]. Among all ratings, 56/64 (87.5%) represented only three levels (Worse, Minimally Worse, No Change) of the seven-point scale. The lack of variation in cases was affirmed in the statistical model where case variance was not statistically significantly different from zero. A alternative approach to improve reliability would be to take the average rating of several raters, a technique that could also mitigate the variation introduced by differences in individual clinical judgement. A post-hoc analysis assessing reliability by taking the average rating from multiple raters rather than a single rating yielded an ICC(2,k) of 0.87 (95% CI 0.72–0.96) indicating moderate to excellent reliability. Given this improvement in reliability, the average of multiple raters should be used in future application of the CGA. Use of an average of rating has been suggested in other disease-specific scales to improve CGI reliability [31].

The CGA is a useful measurement of clinical change over time or, in the context of a clinical trial, after an intervention. The resolution of a seven-point scale was chosen to ensure a conceptual understanding of a rating that a slider or visual analog scale may not offer.

The language used for each level efficiently demarcates a minimally important clinical difference. When assessing an intervention, due to the progressive nature of Proteus syndrome and finding the average rating among a natural history cohort was Minimally Worse, we propose that ratings of No Change or any rating of improvement may be clinically meaningful. When comparing groups or an individual with multiple evaluable timepoints, a one-point change in the CGA rating is likely to designate a minimal clinically important difference in Proteus syndrome. However, anchoring with other observational and patient or clinician reported outcomes obtained contemporaneously with CGA timepoints may better define clinically meaningful change. It is important to recognize that the CGA only includes clinical information available by inspection of photographs and radiographs and does not include other clinically meaningful data such as pain and quality of life. It may be advantageous to consider including these data along with photographs and radiographs. Future research is needed to evaluate the validity of the scale through association with other objective measures of Proteus syndrome (e.g., pulmonary function, walking distance, pain) and correlation with patient global impression scales.

A possible ascertainment bias may exist as the data were retrospectively gathered from available photographs and radiographs. We reviewed the clinical records of all participants on our natural history of Proteus syndrome study and identified only eight eligible records. It may be the case that these images are available because an individual experienced a significant clinical event or worsening, which subsequently led to their repeat evaluation at the NIHCC. Future uses of the CGA should consider prospective and consistent photography and radiographic techniques to avoid potential bias of retrospectively collected data as was used in this study. However, another explanation for the limited number of records is that most individuals have directed radiographs of sites of overgrowth and not serial skeletal surveys which were part of our eligibility criteria.

## Conclusions

The CGA addresses challenges of evaluating an individual with a complex phenotype within a heterogenous population. Rare disease research can often be limited by a small population, particularly when phenotypic heterogeneity exists that further subdivides a disorder. The CGA can be used not only to demonstrate the progressive natural history of Proteus syndrome, as was done in this study, but also to support outcome measurements in future interventional studies. The results from this study support additional research to improve the validity of the CGA scale and its use as a clinically meaningful assessment.

## Supplementary Information

**Additional file 1: Fig. S1.** Cases reviewed by raters.

**Additional file 2: Table S1.** Chronology key and rater responses.

## Data Availability

The datasets generated during and/or analyzed during the current study are available from the corresponding author on reasonable request.
