# Supplementary material for: Development of the Clinical Gestalt Assessment: a visual clinical global impression scale for Proteus syndrome
Source: Orphanet J Rare Dis. 2022 Apr 23;17:173. doi: 10.1186/s13023-022-02325-6 (PMC9034583; doi:10.1186/s13023-022-02325-6)

Case 1

Case 1

Circle | Square

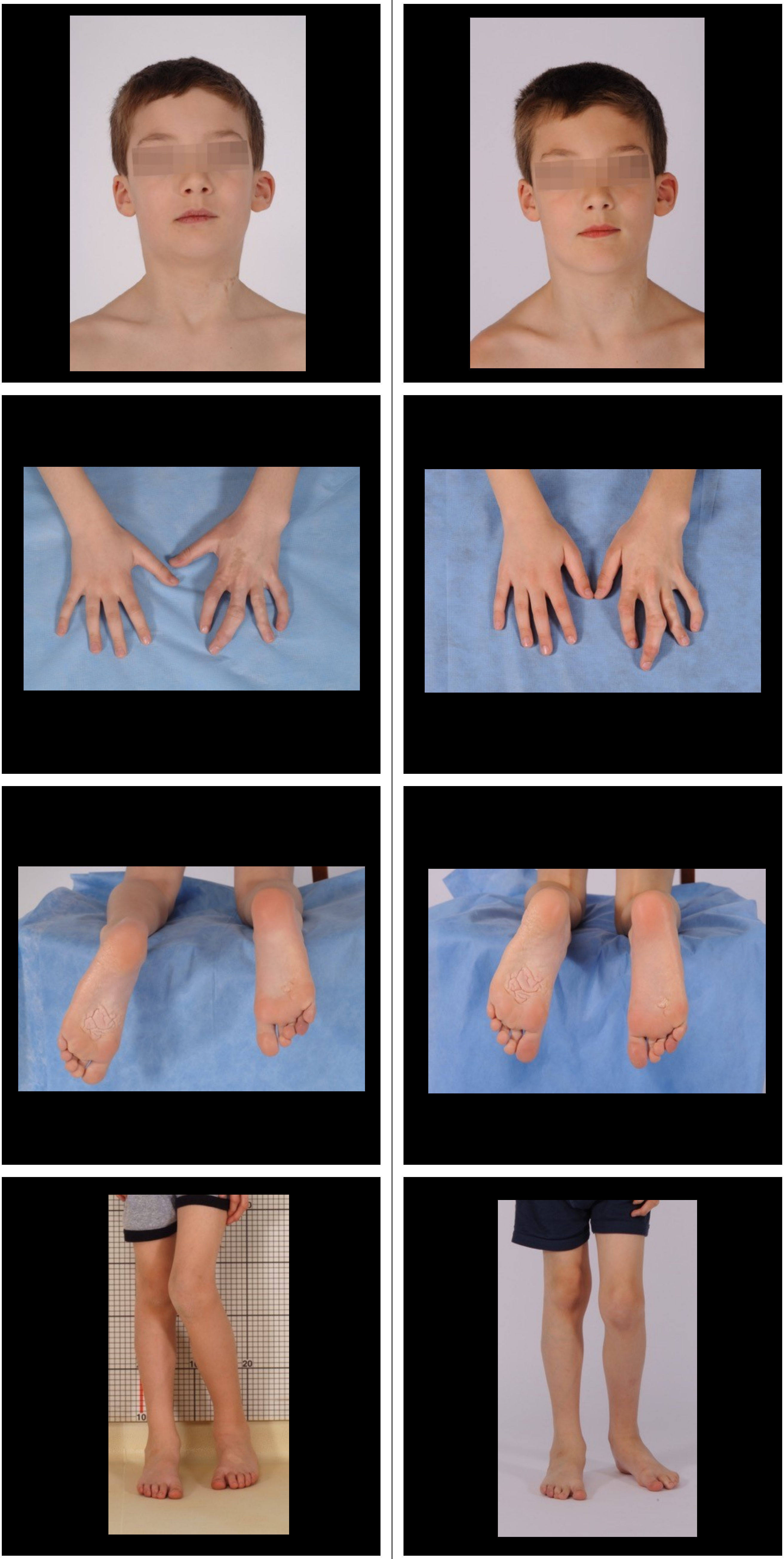

Case 1

Circle

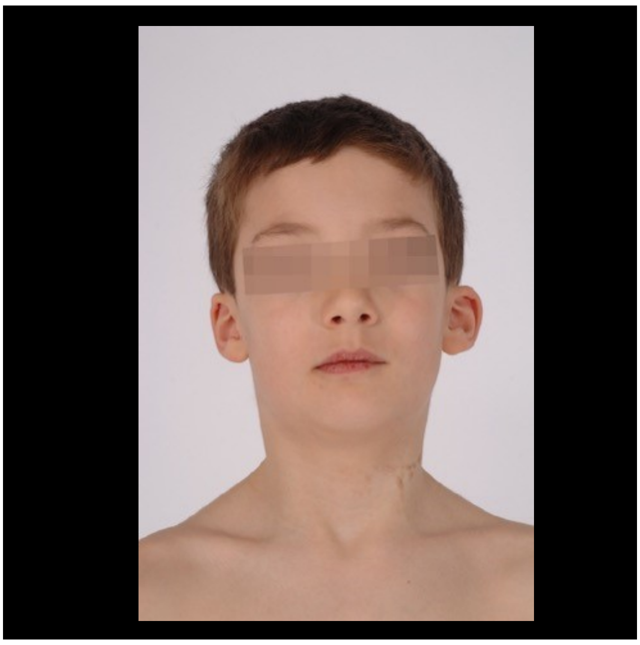

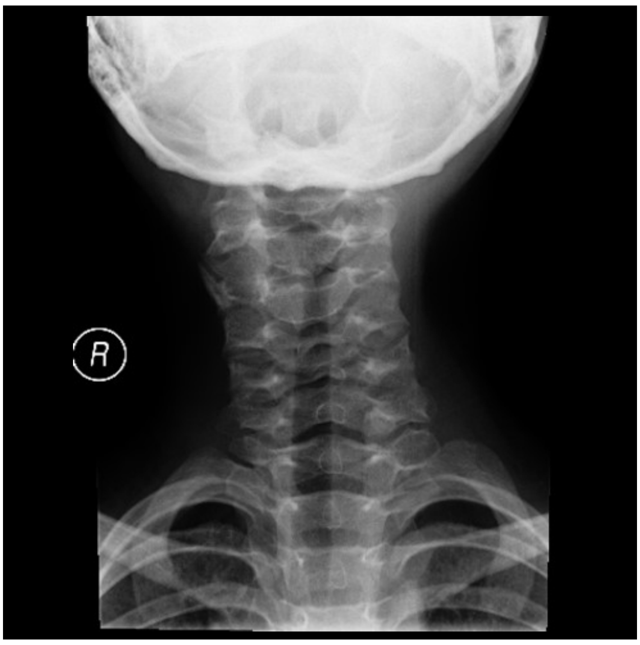

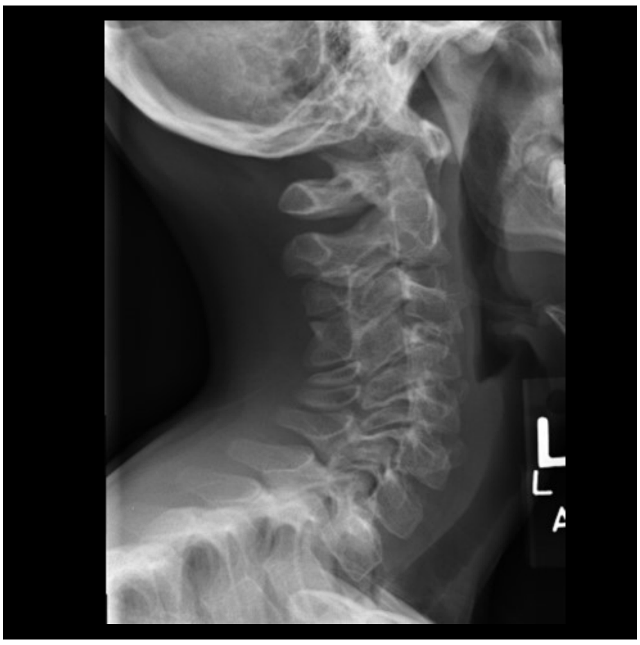

Square

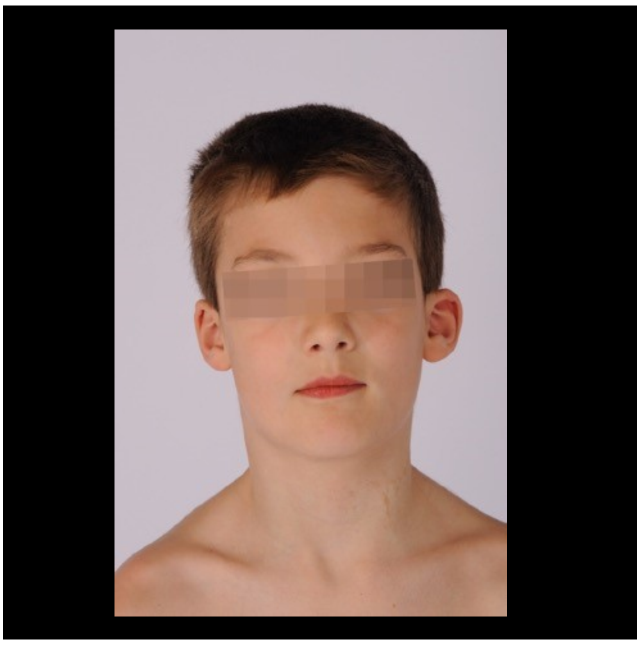

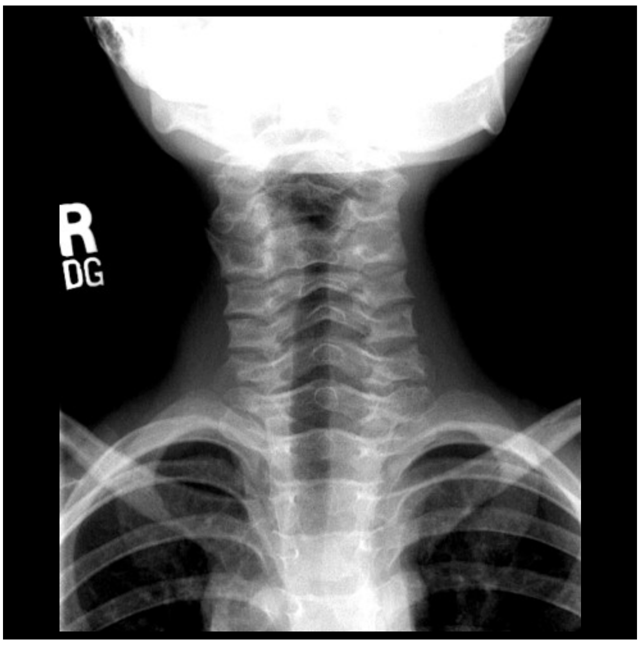

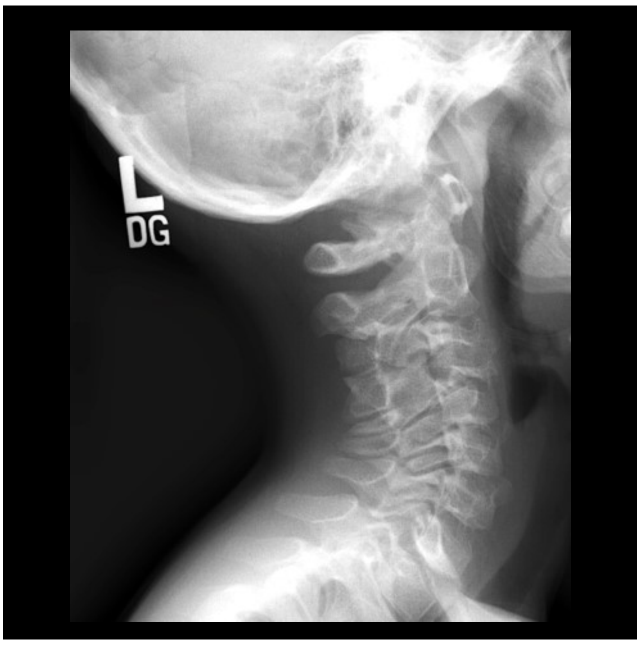

Case 1

Circle	Square
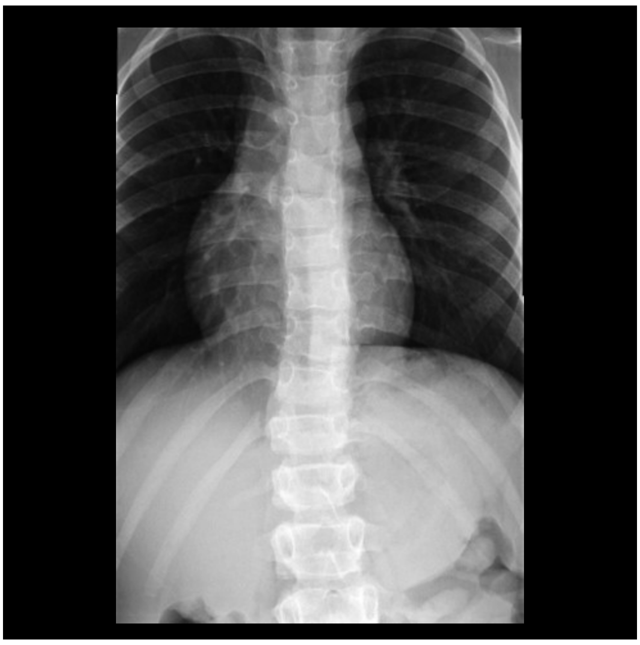	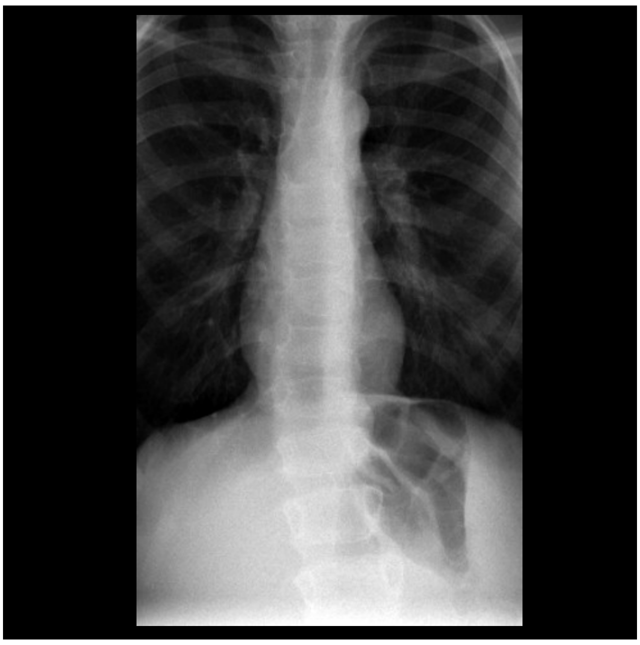
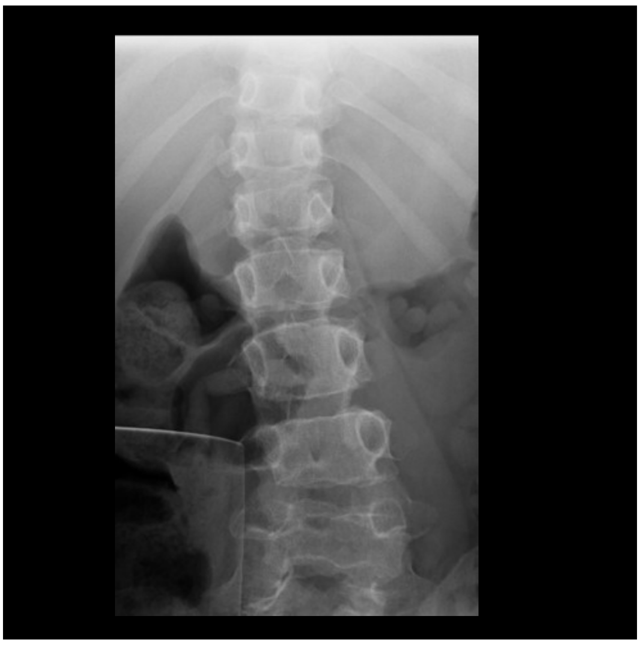	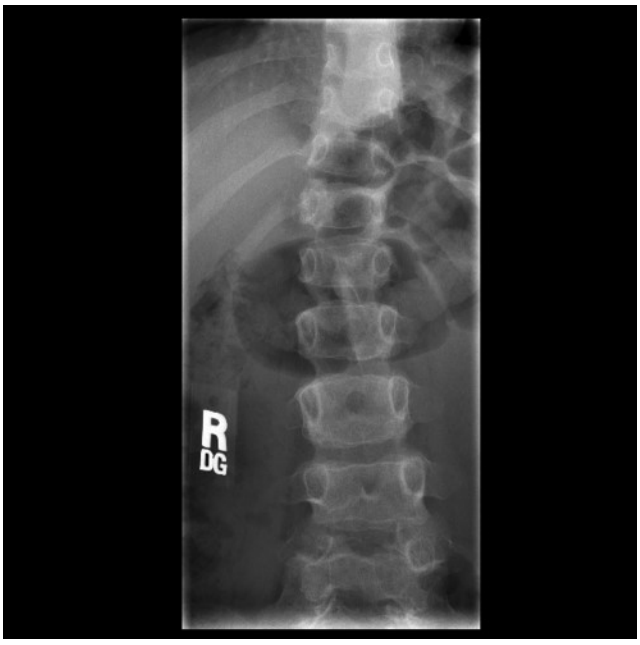

Case 1

Circle	Square
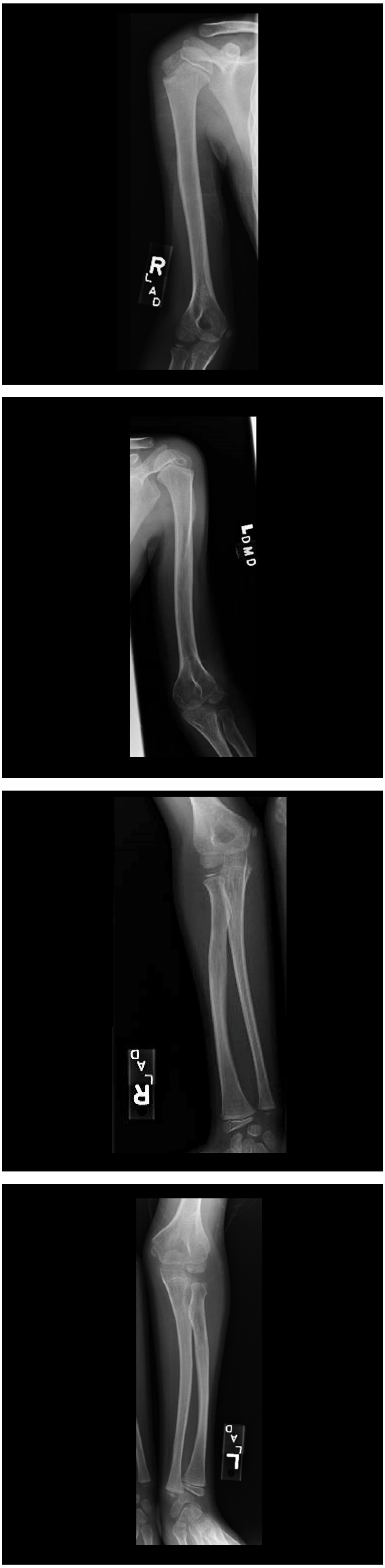	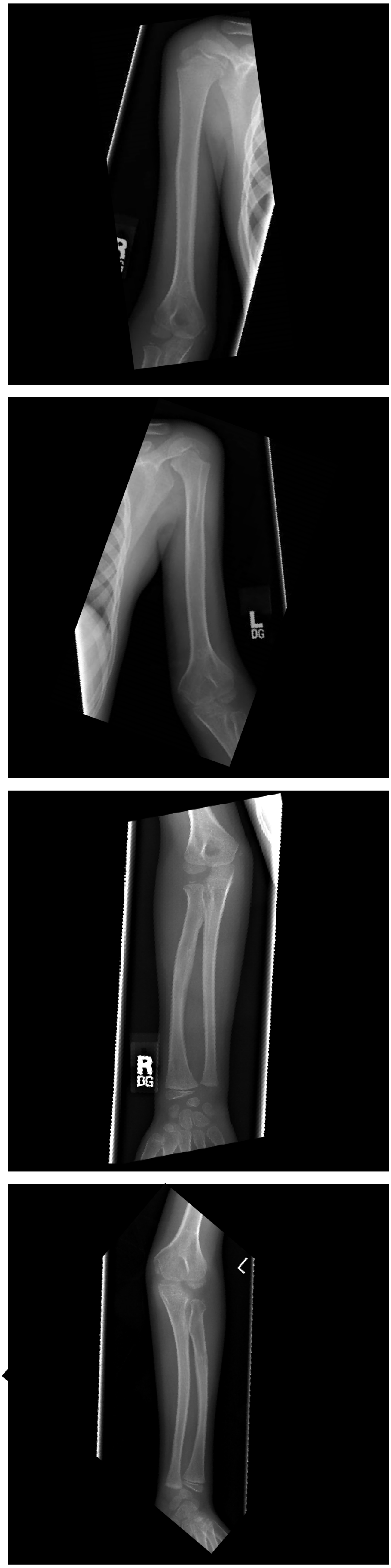

Case 1

Circle

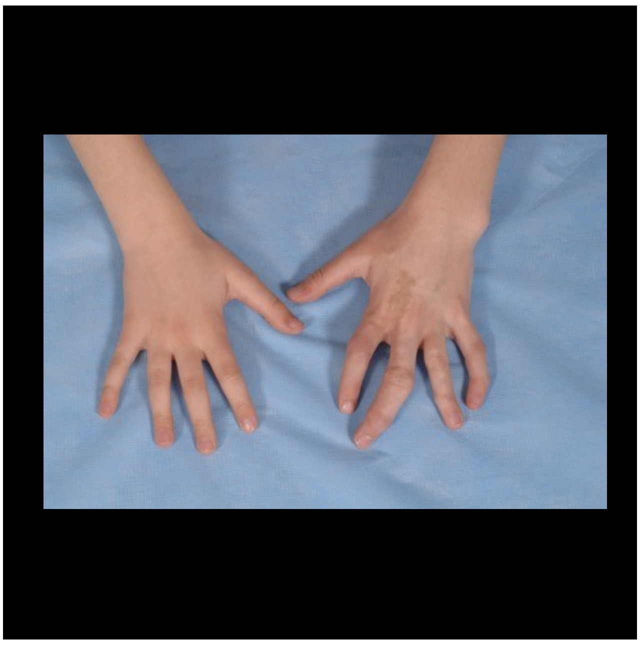

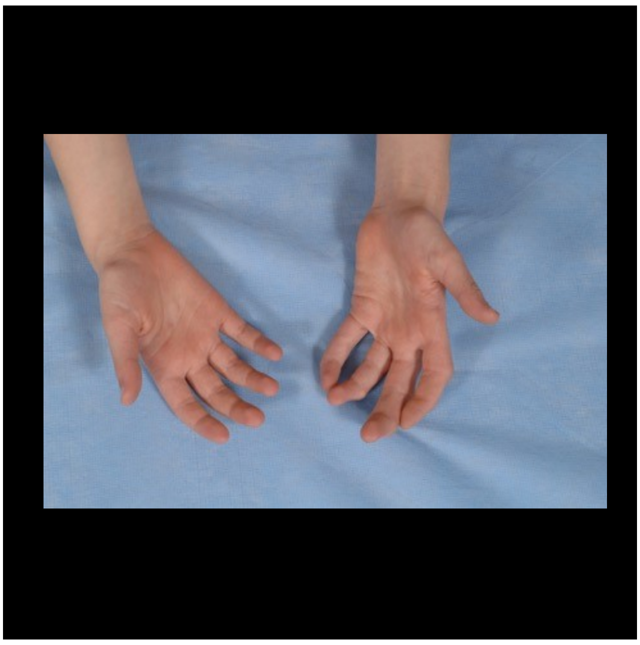

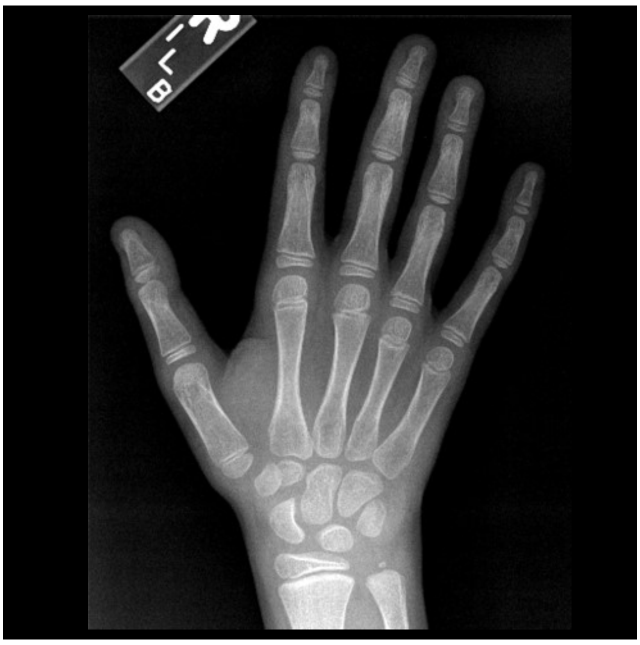

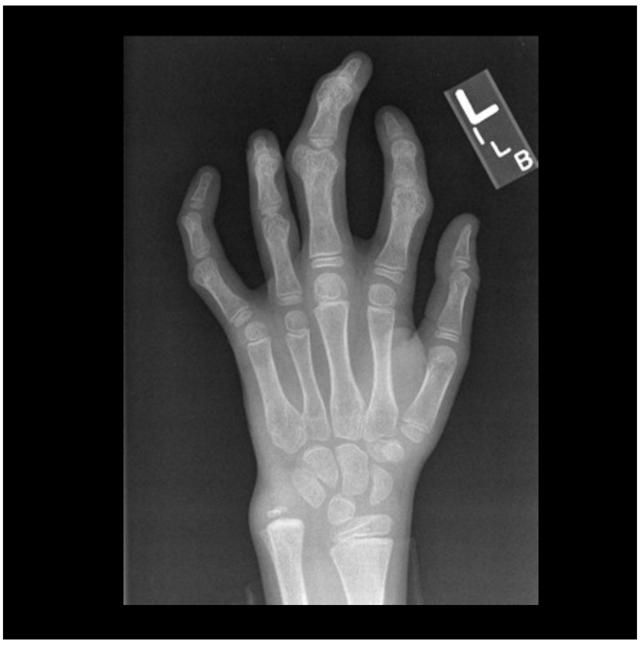

Square

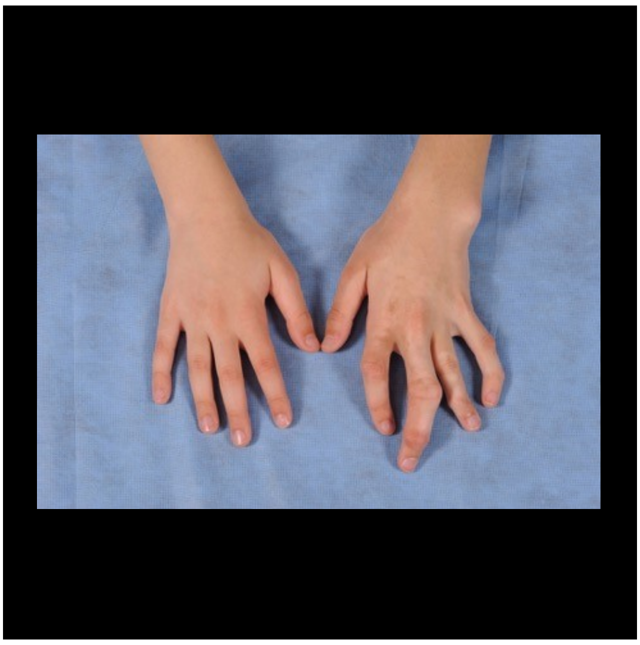

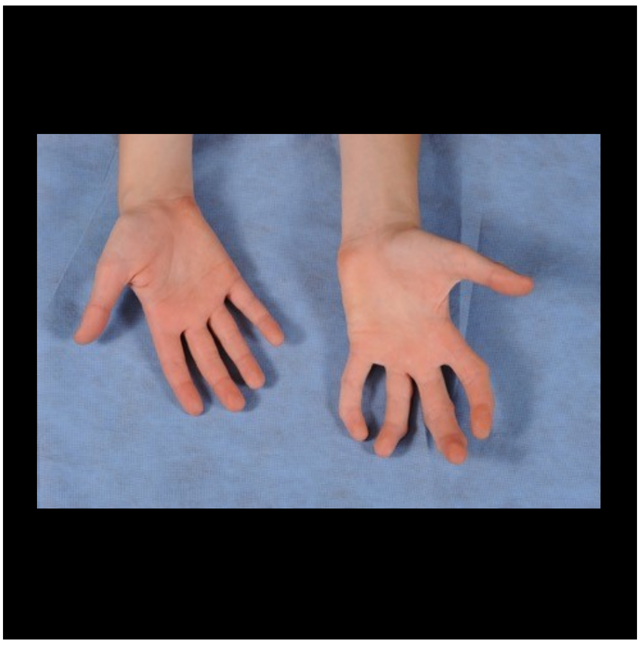

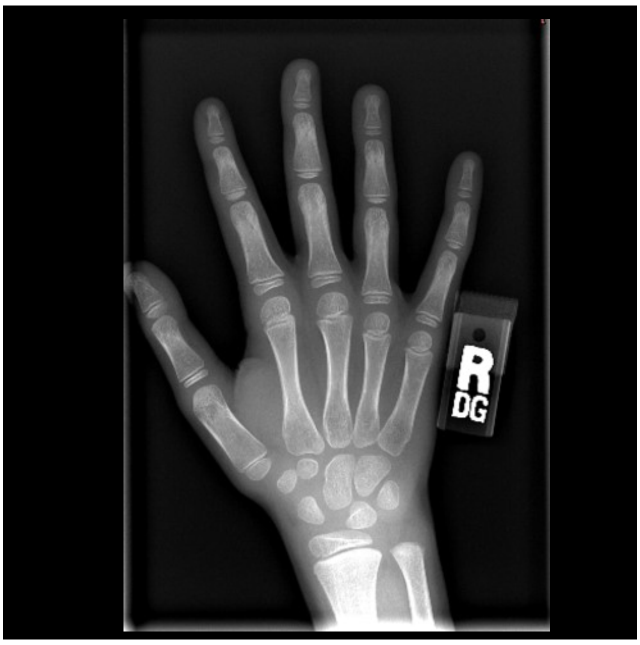

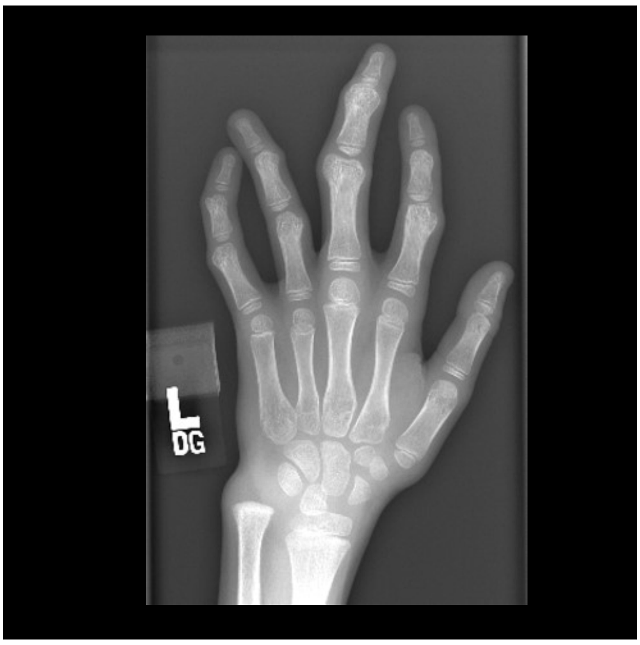

Case 1

Circle

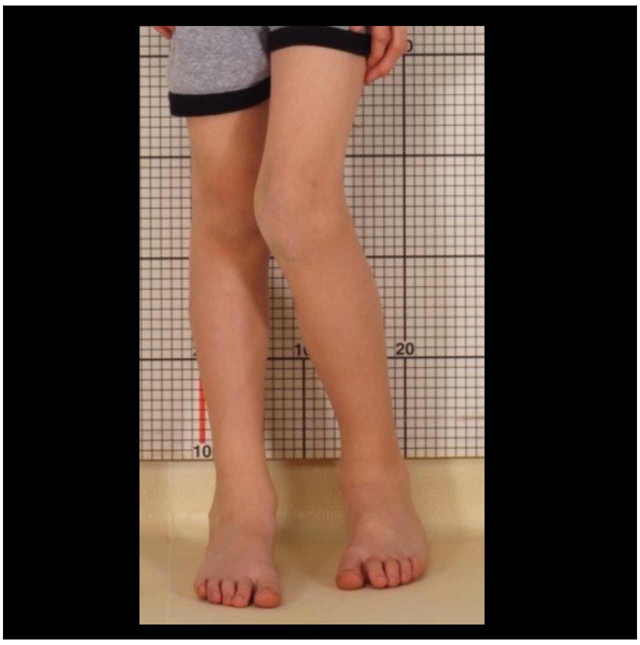

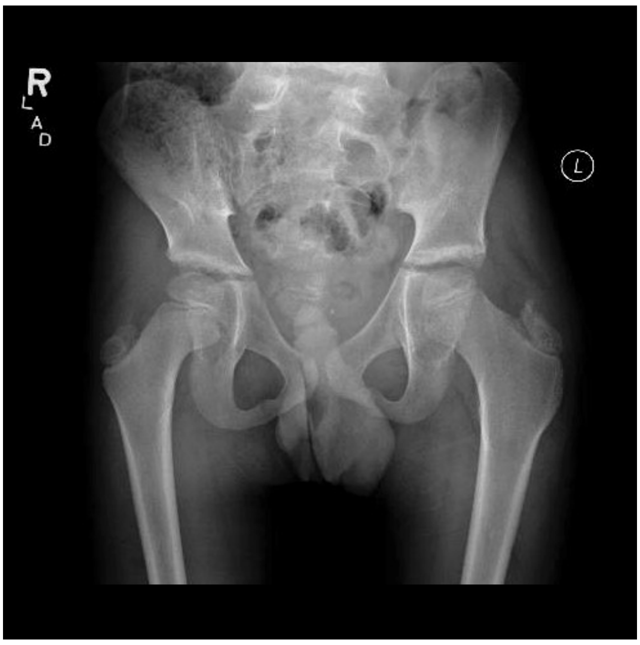

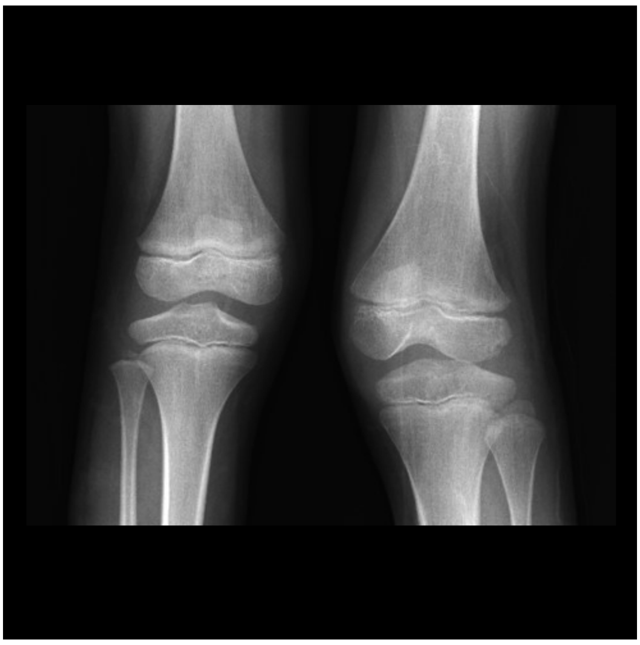

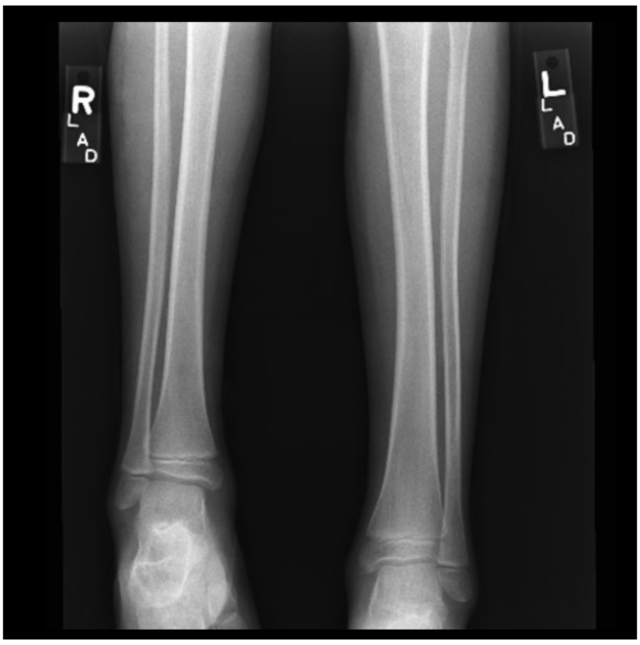

Square

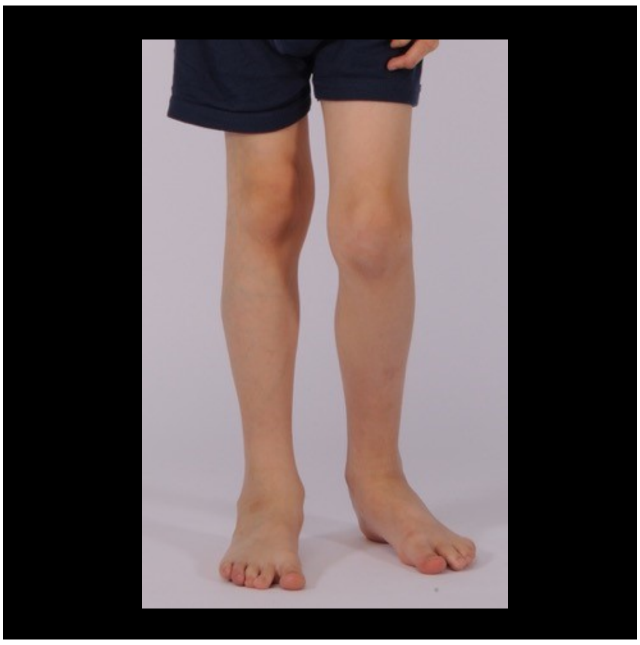

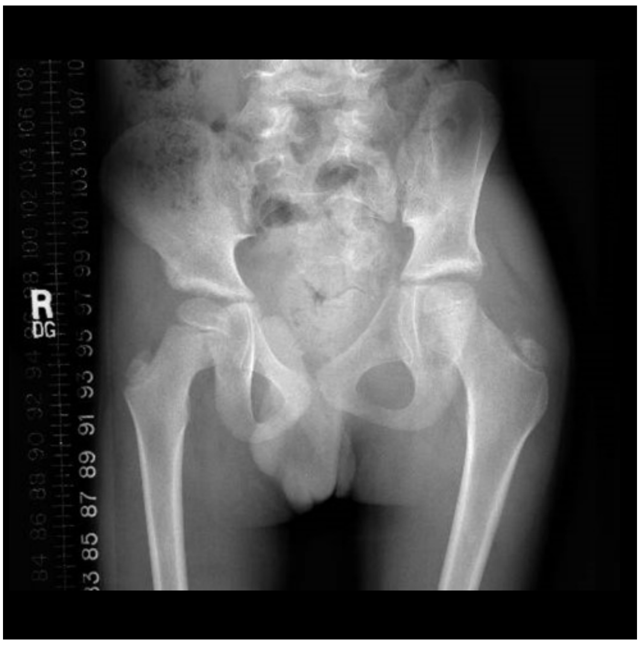

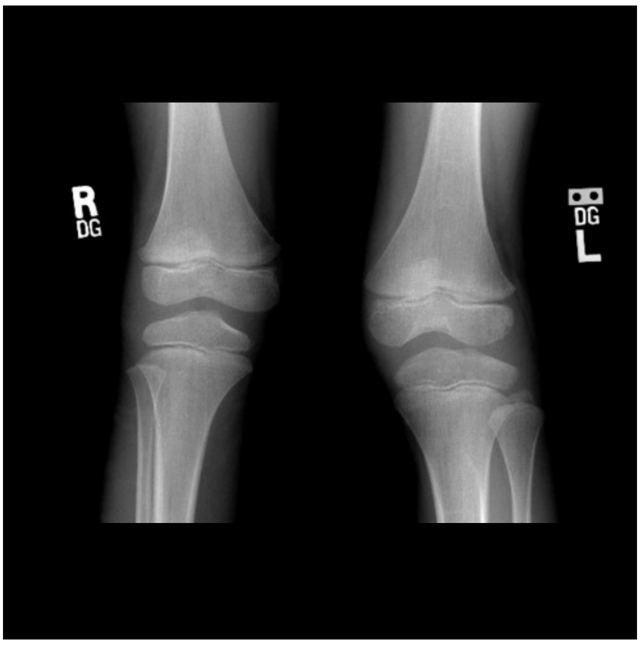

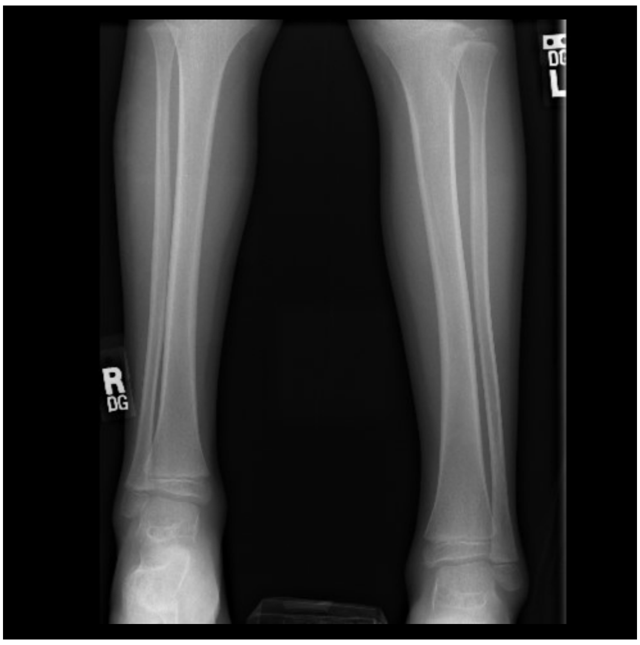

Case 1

Circle

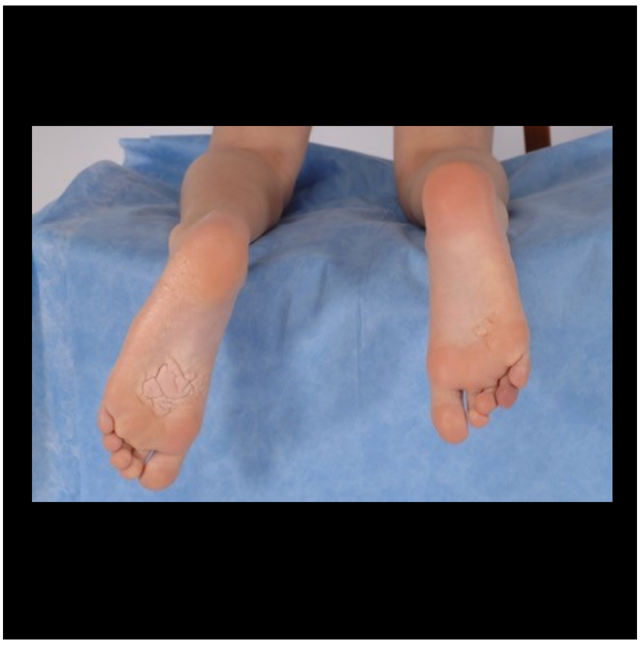

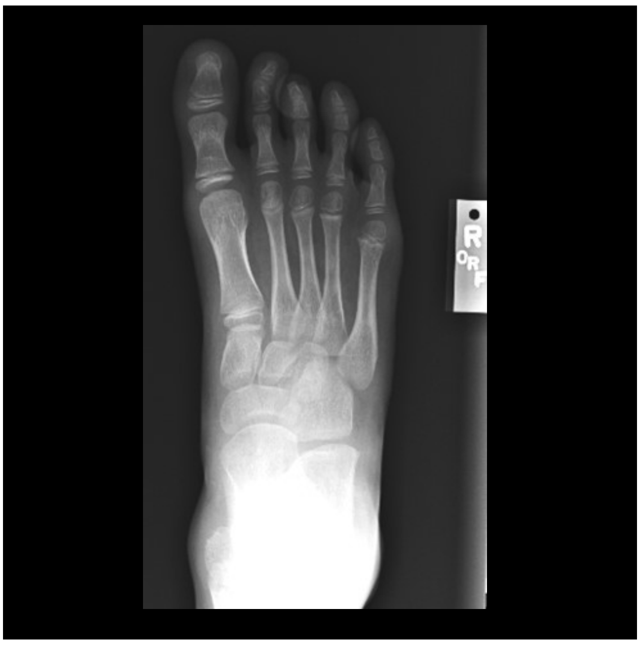

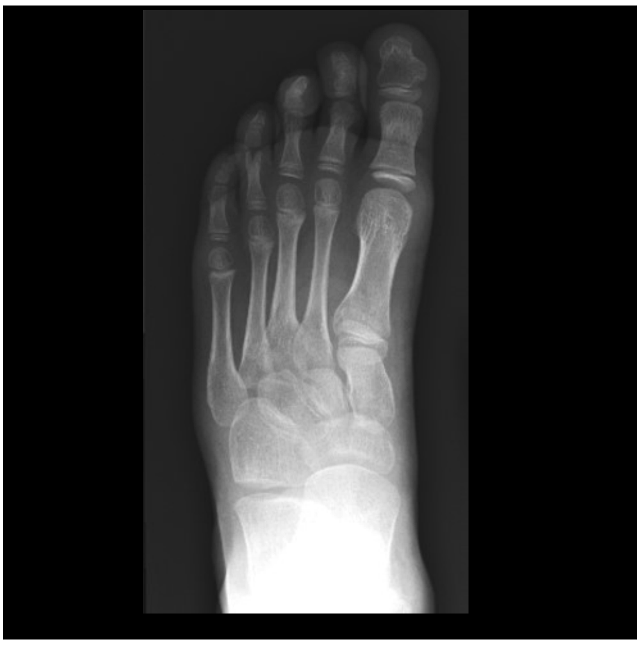

Square

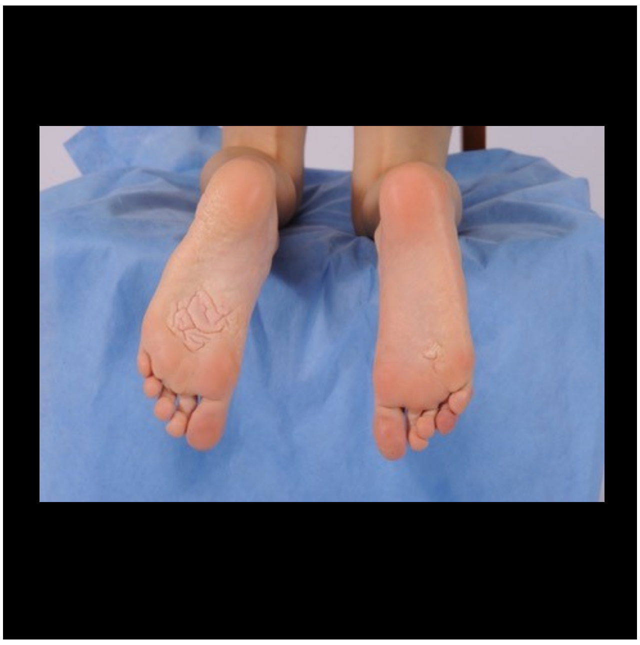

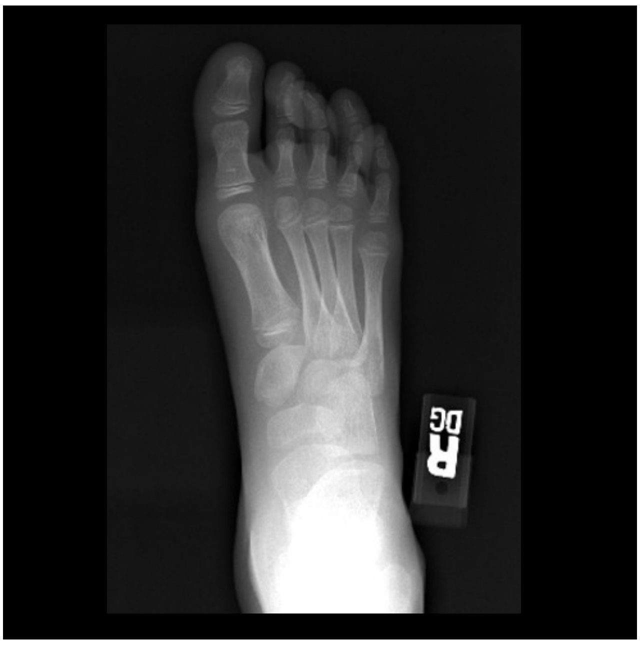

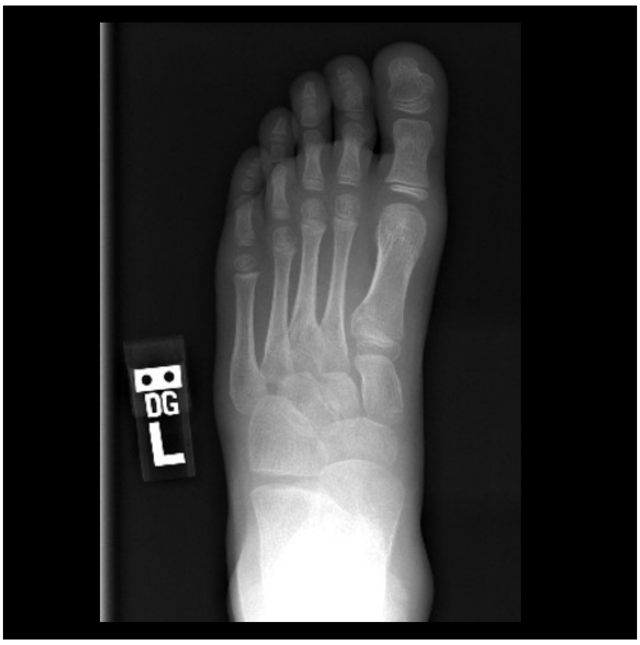

Case 2

Case 2

Circle | Square

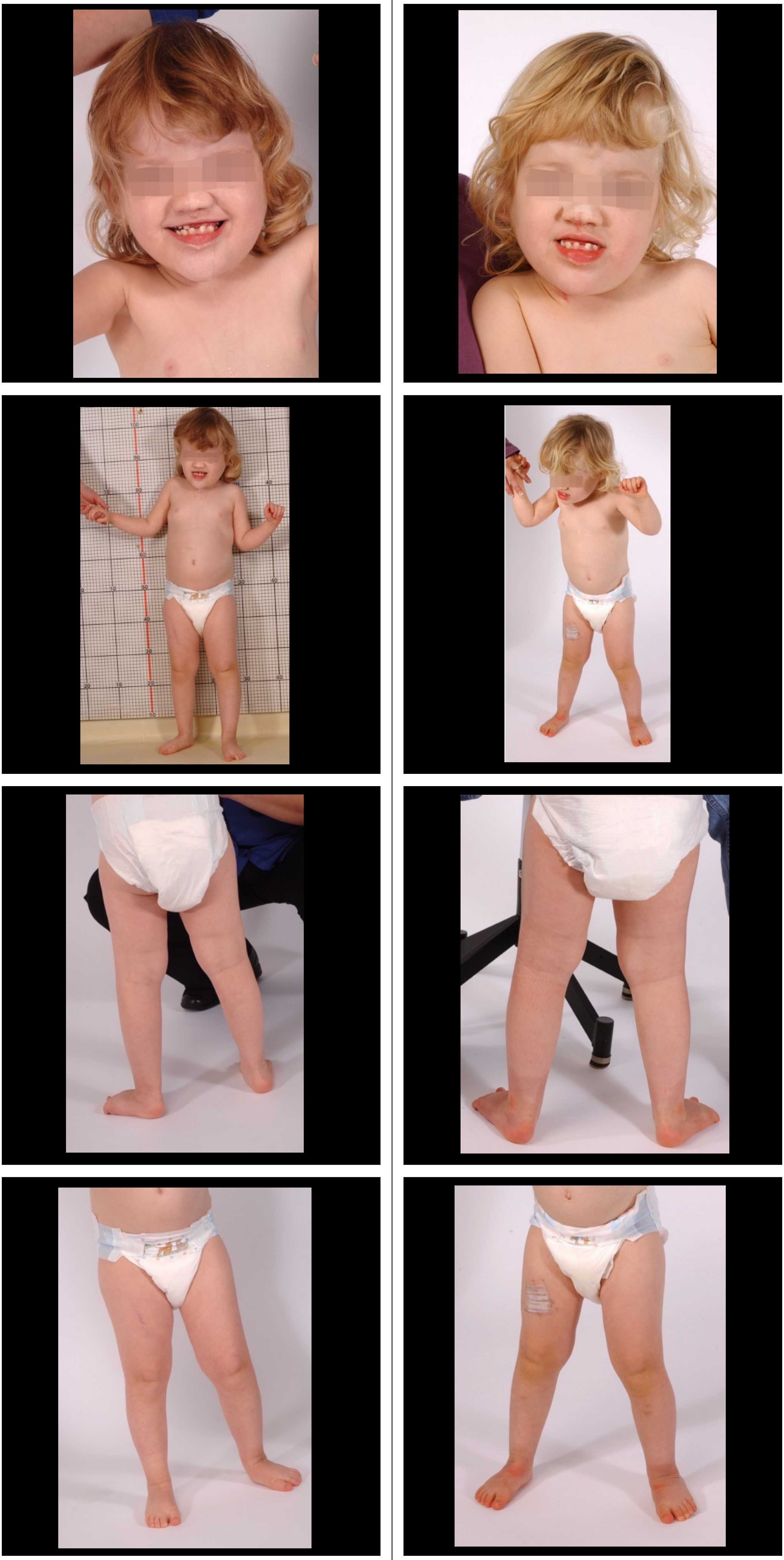

Case 2

Circle

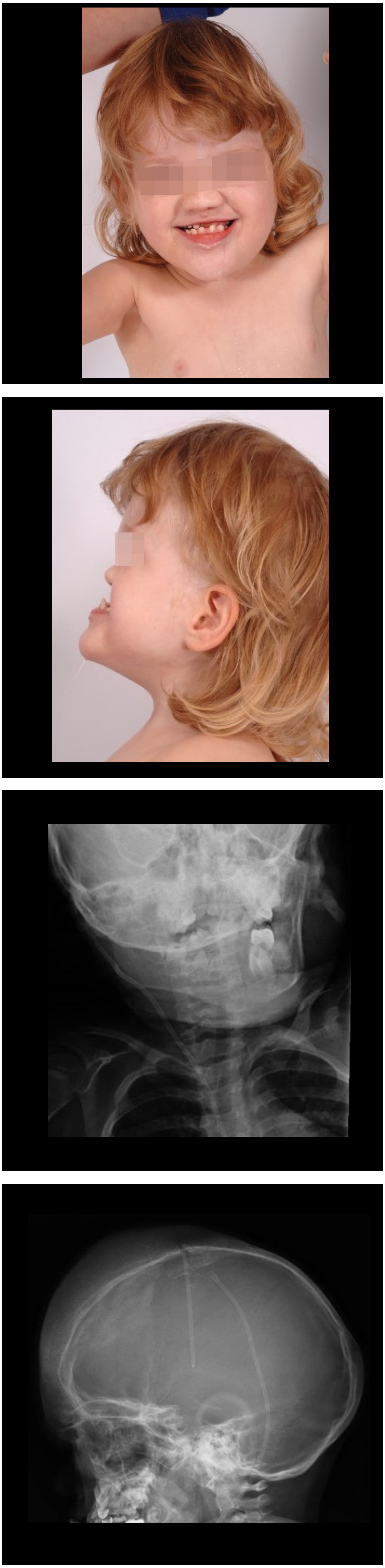

Square

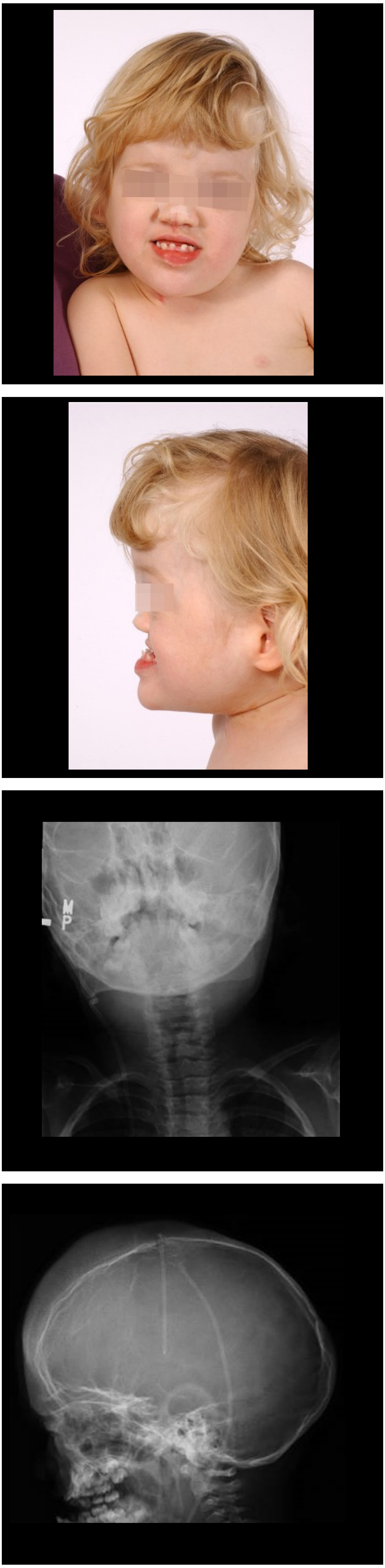

Case 2

Circle

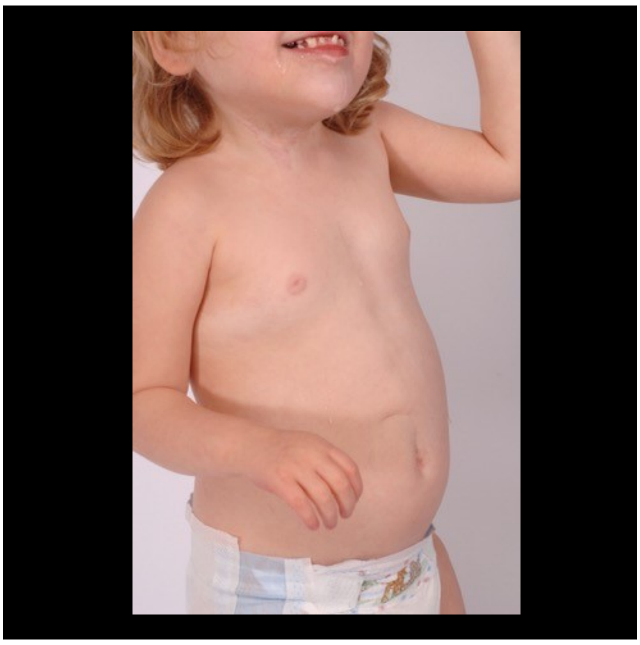

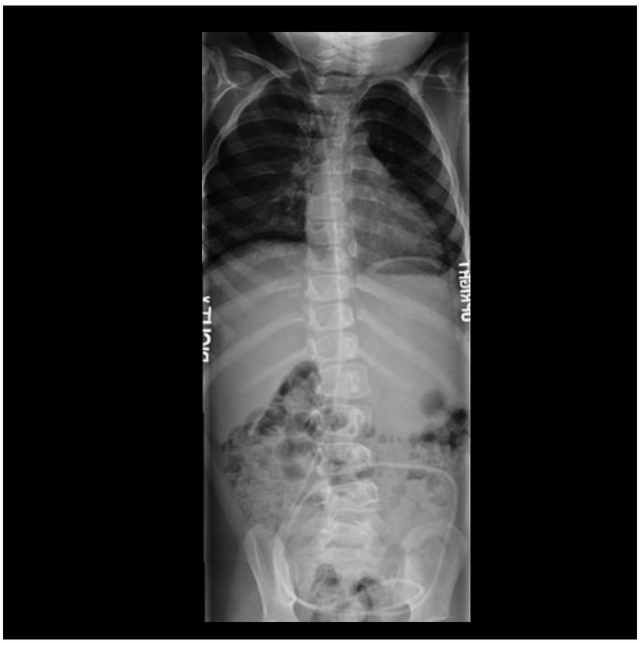

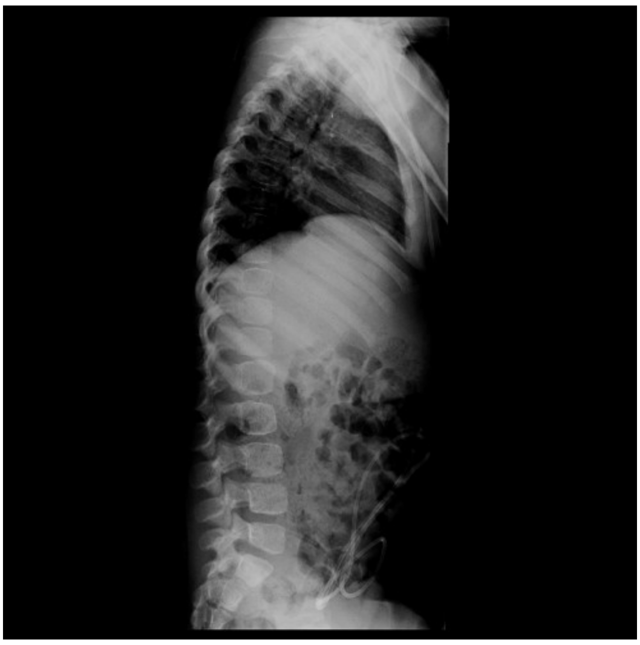

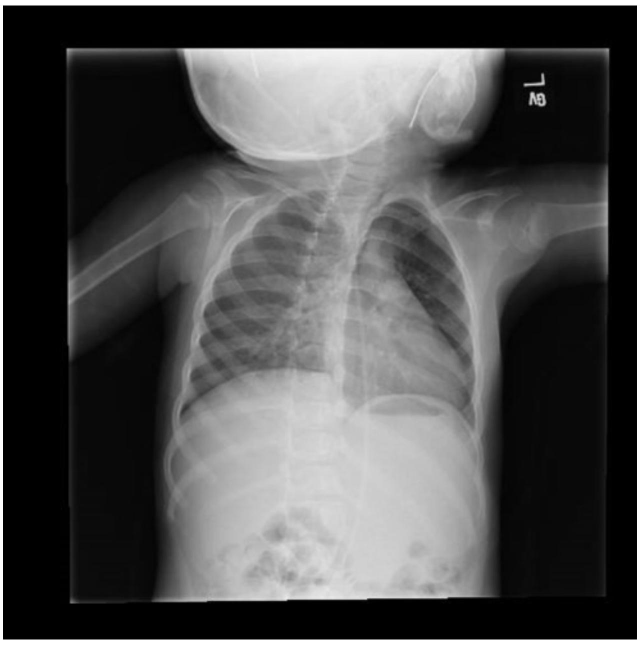

Square

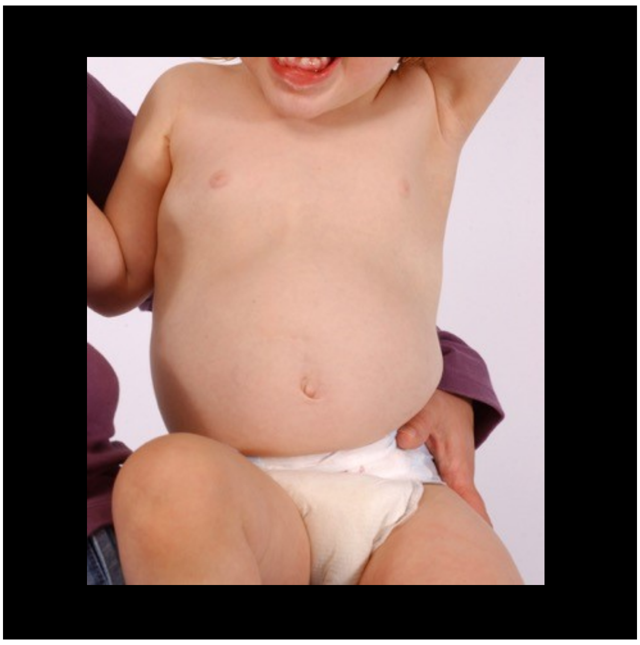

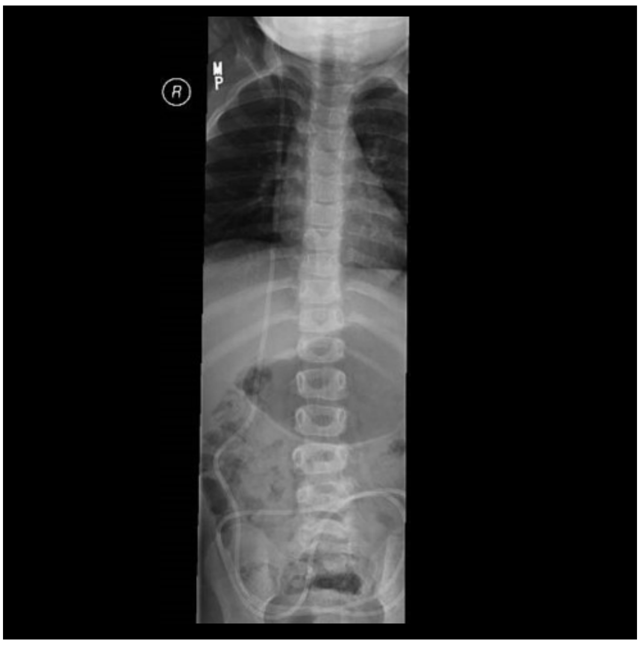

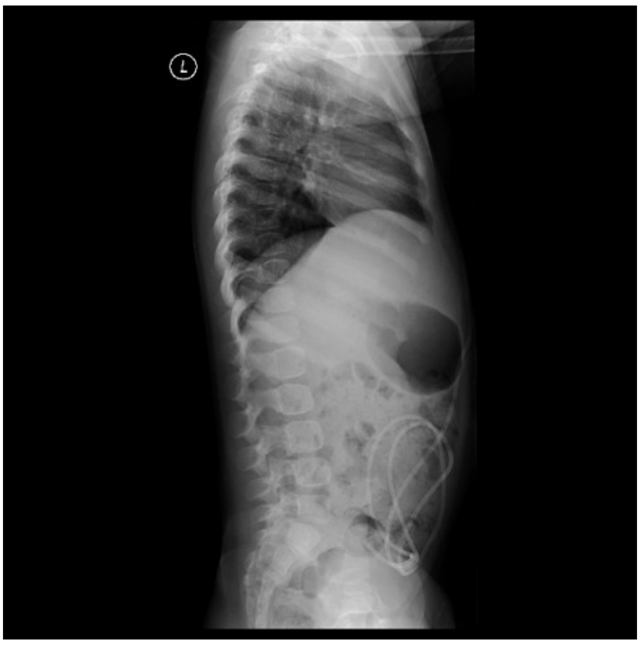

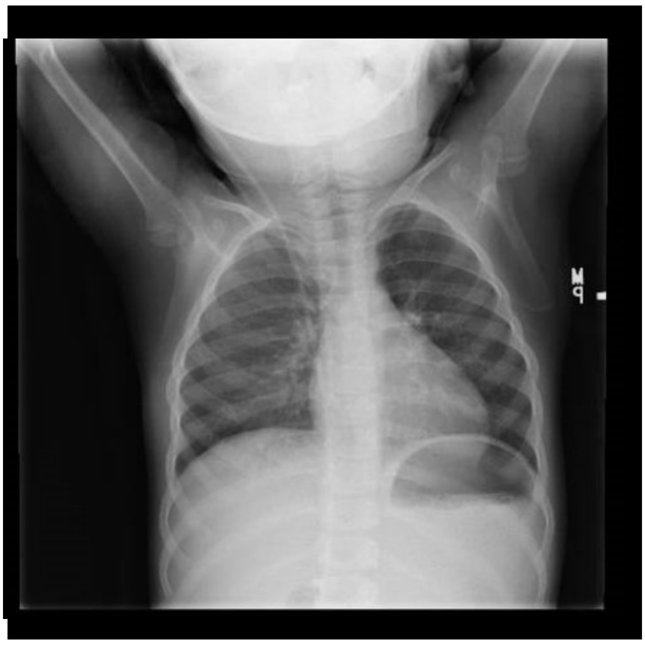

Case 2

Circle

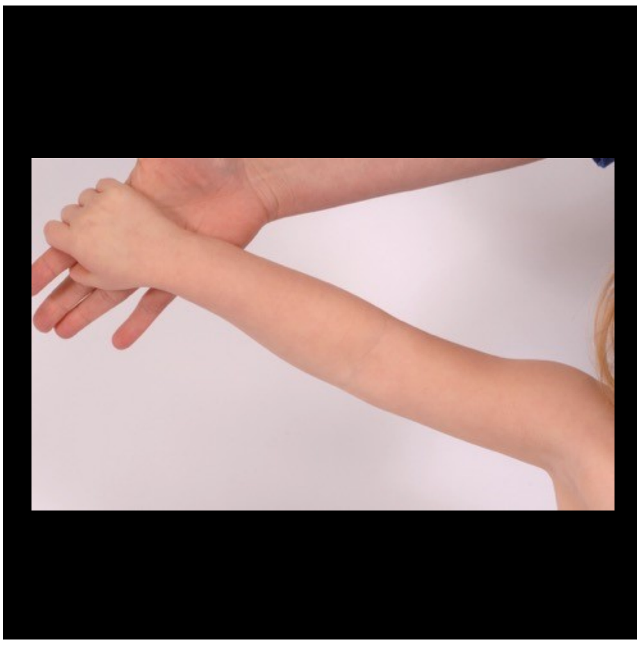

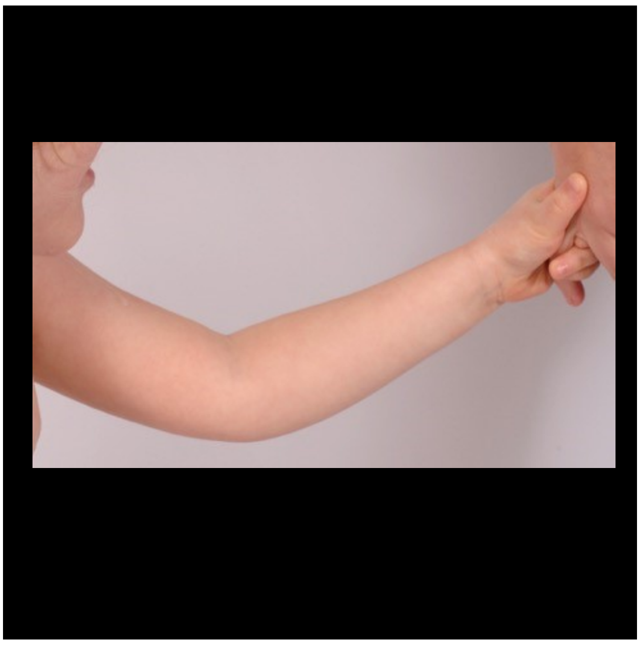

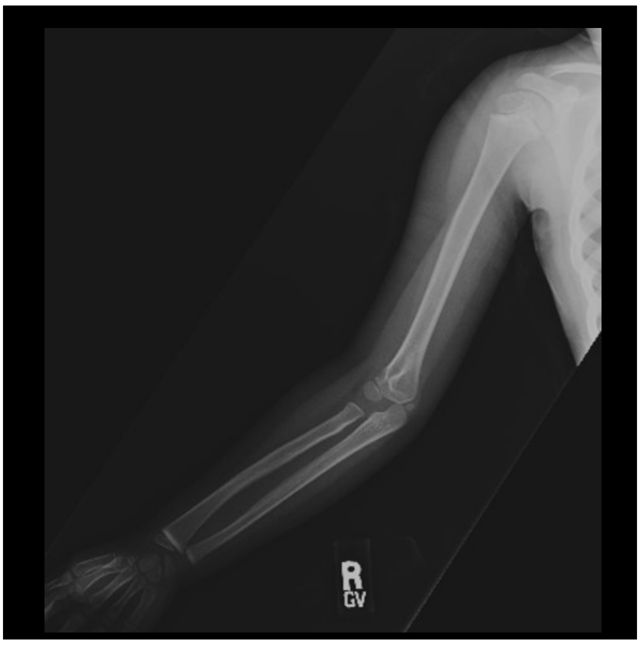

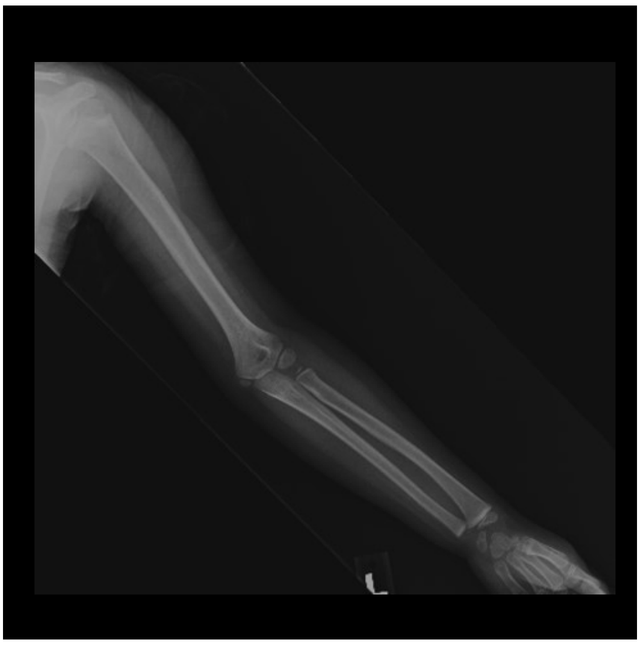

Square

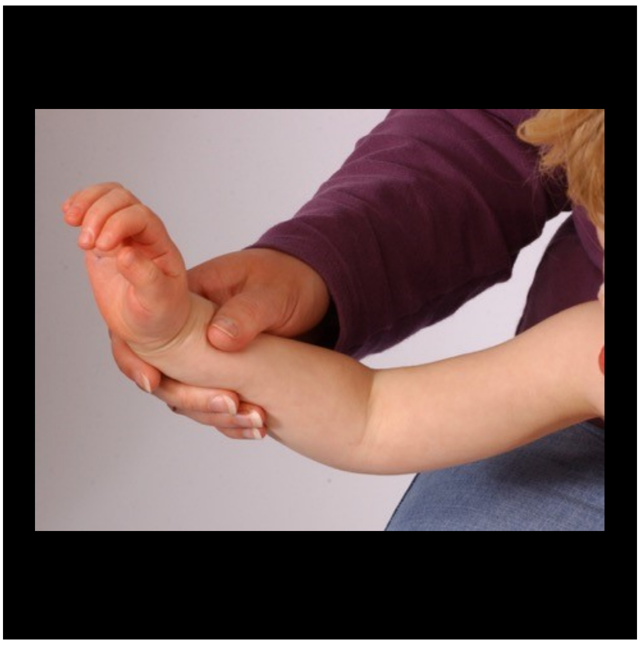

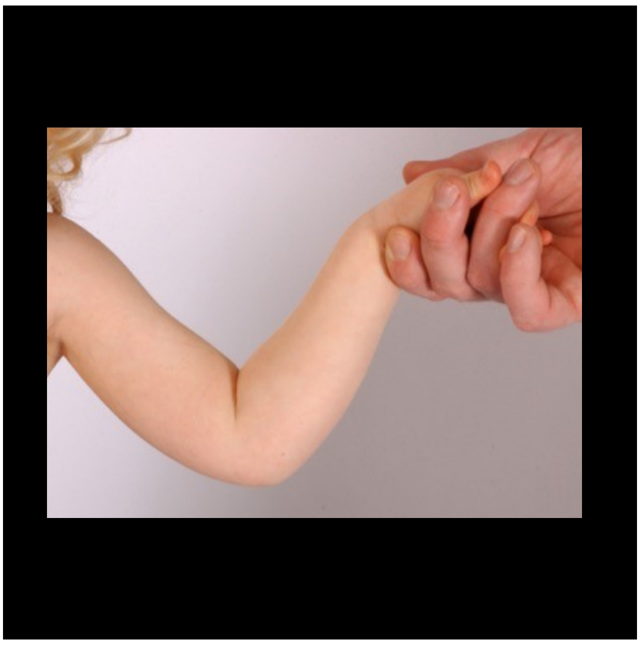

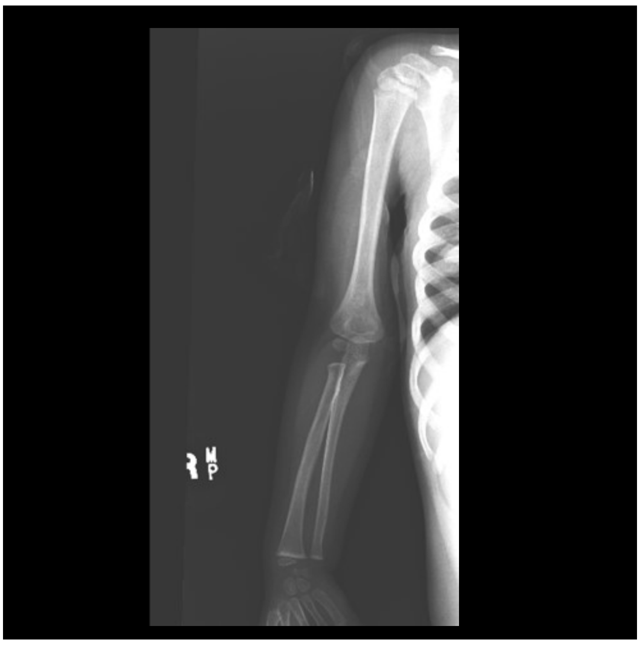

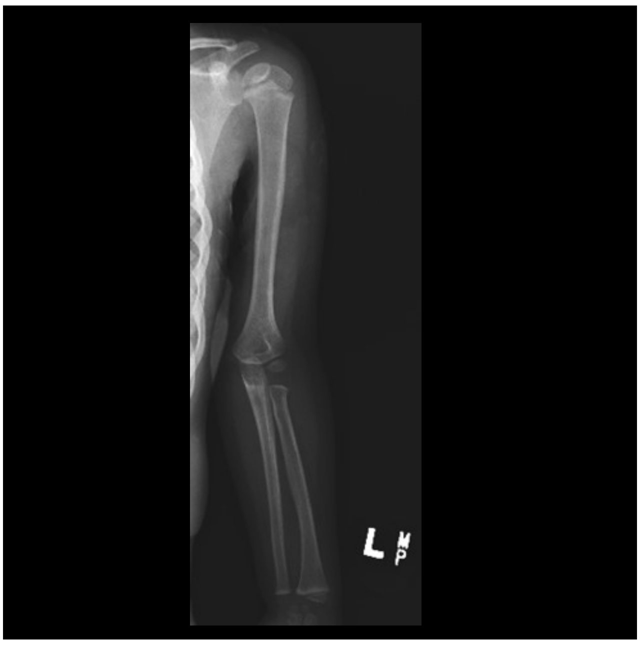

Case 2

Circle

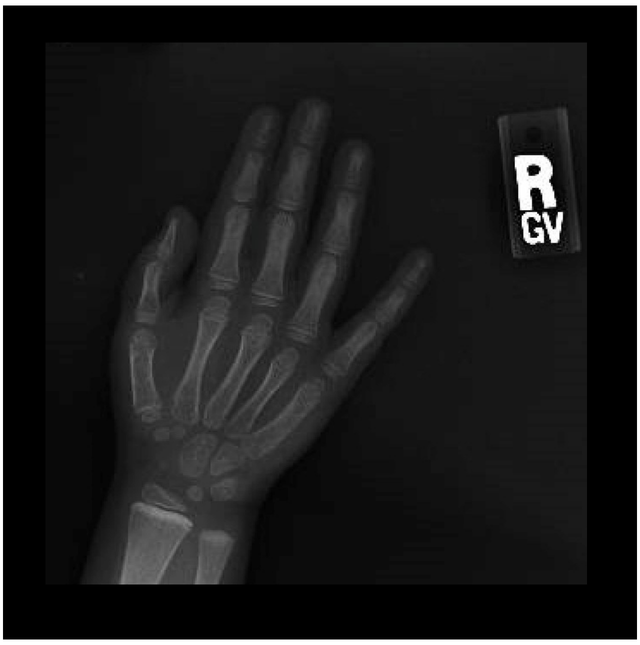

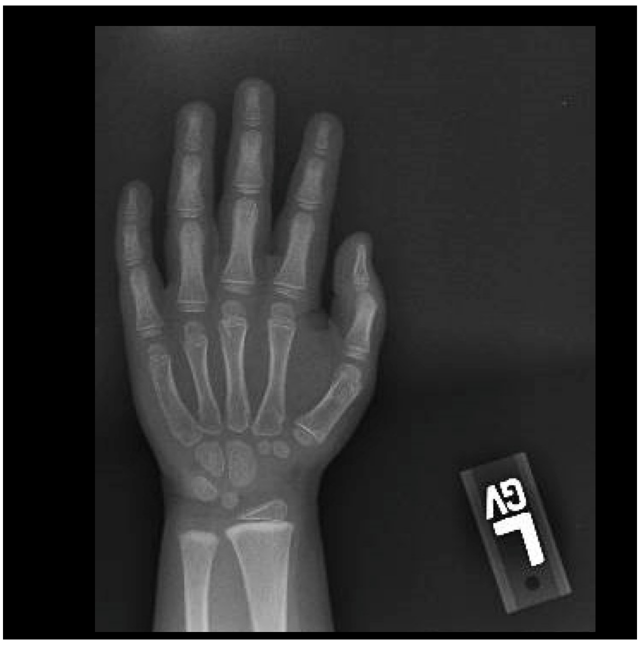

Square

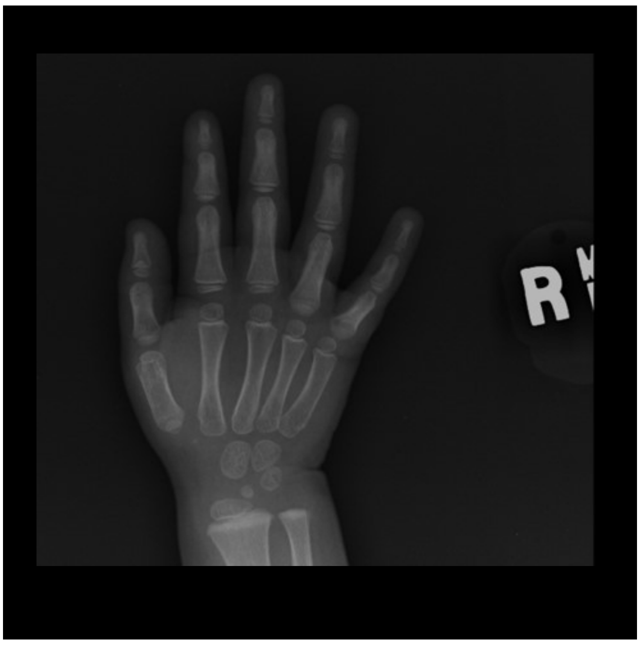

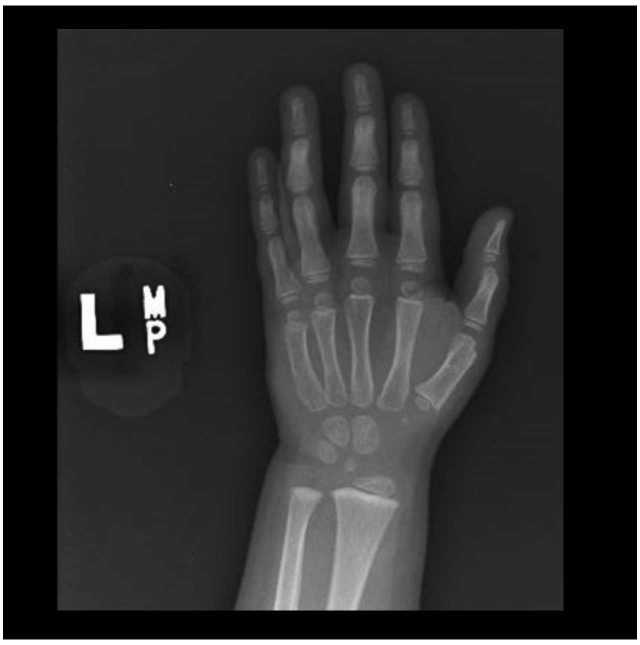

Case 2

Circle | Square

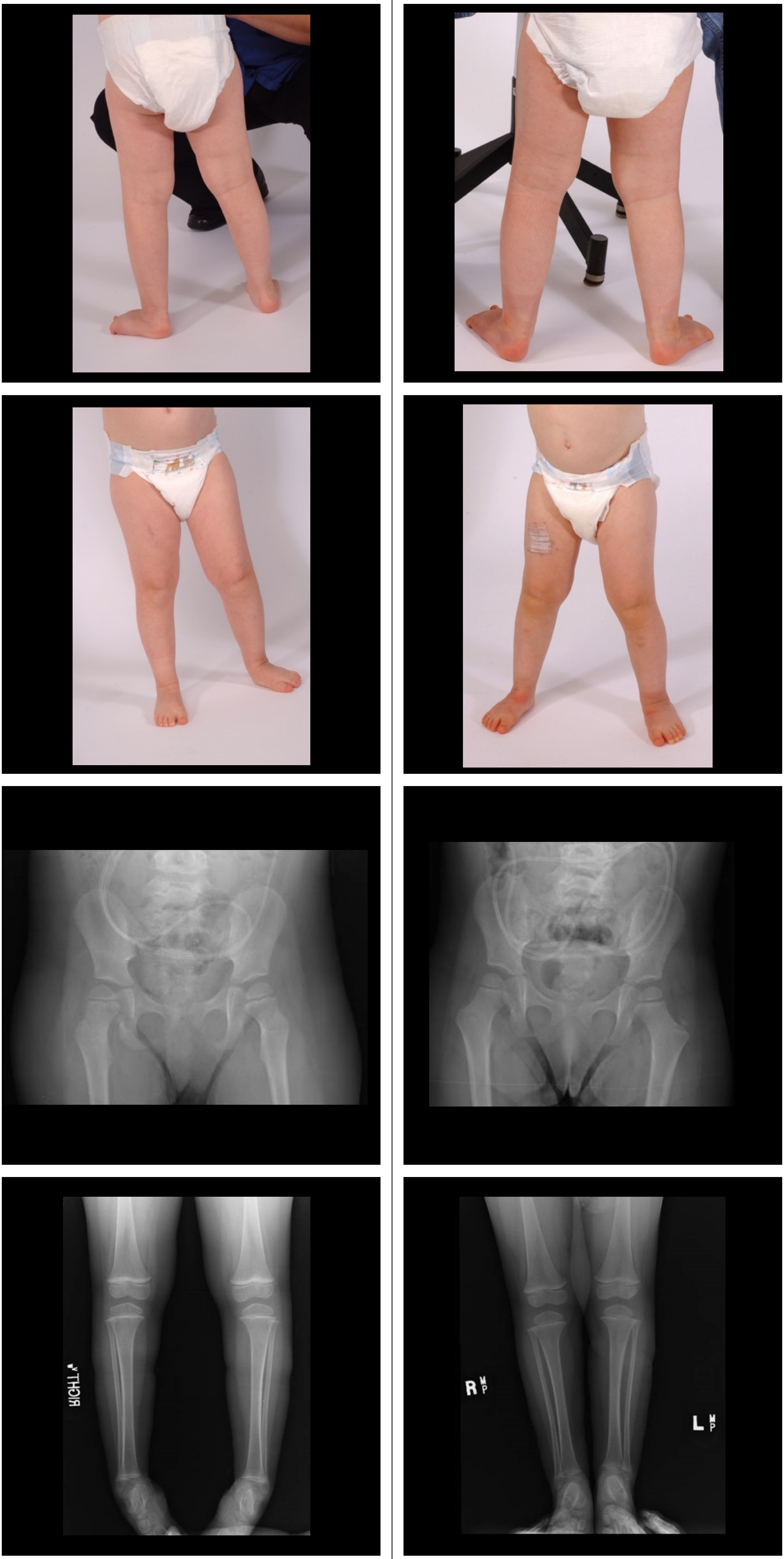

Case 2

Circle

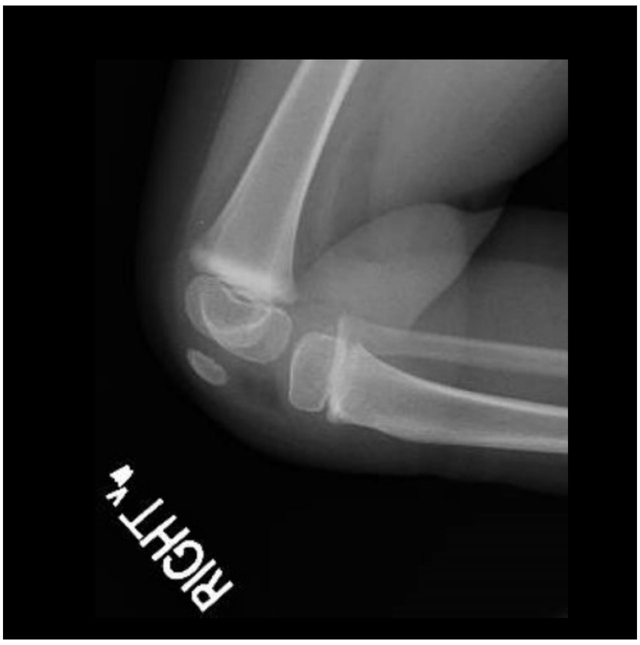

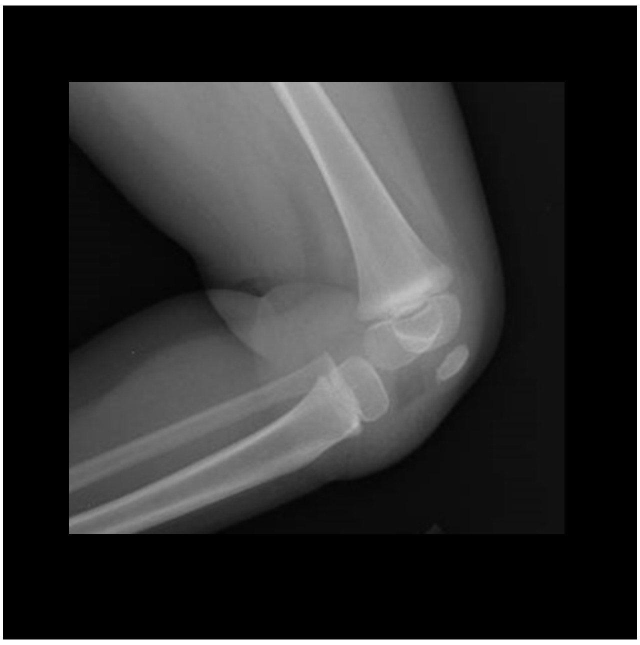

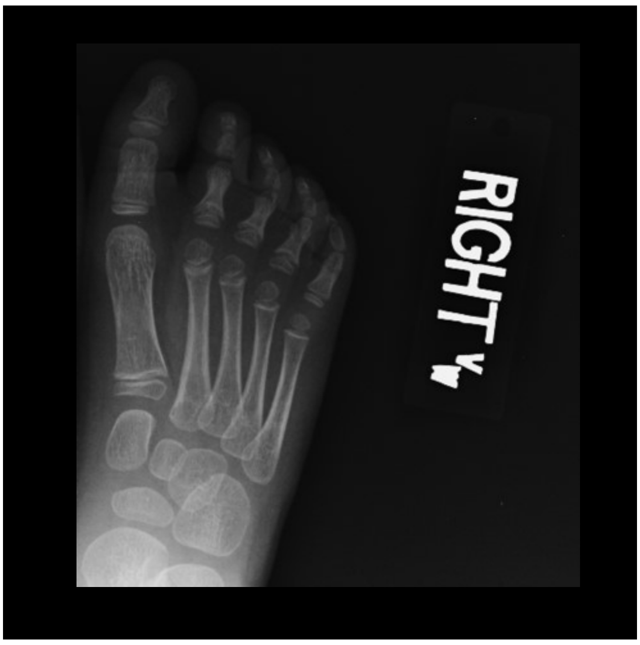

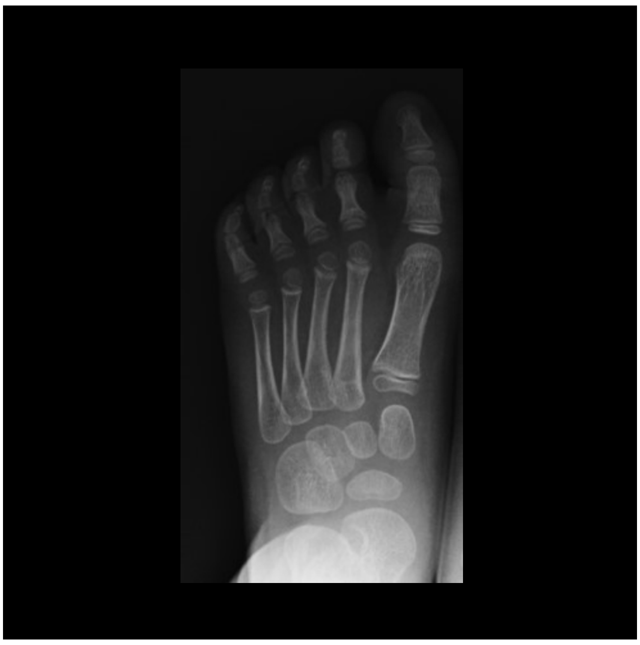

Square

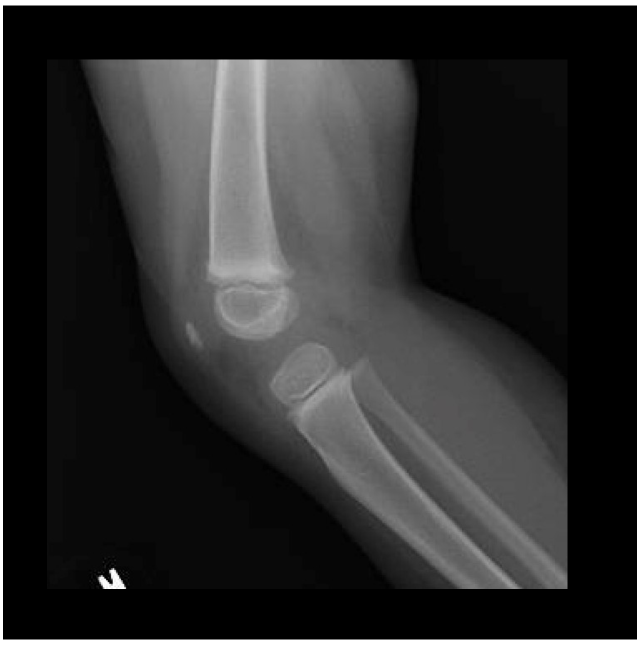

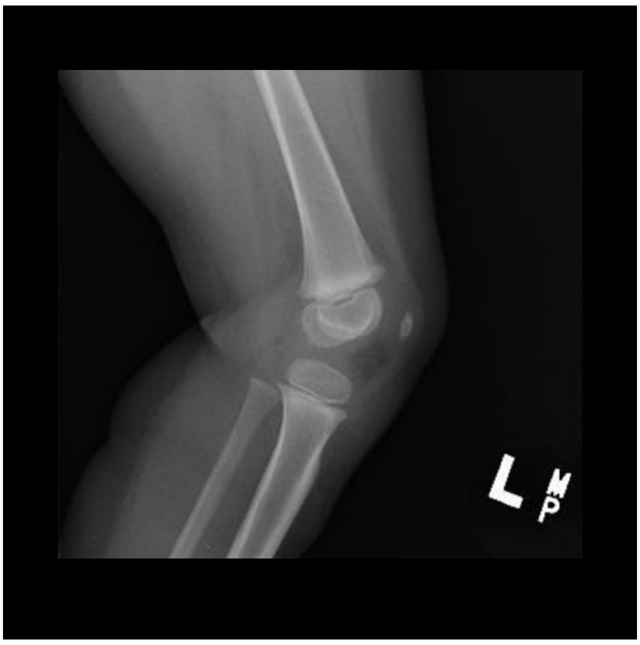

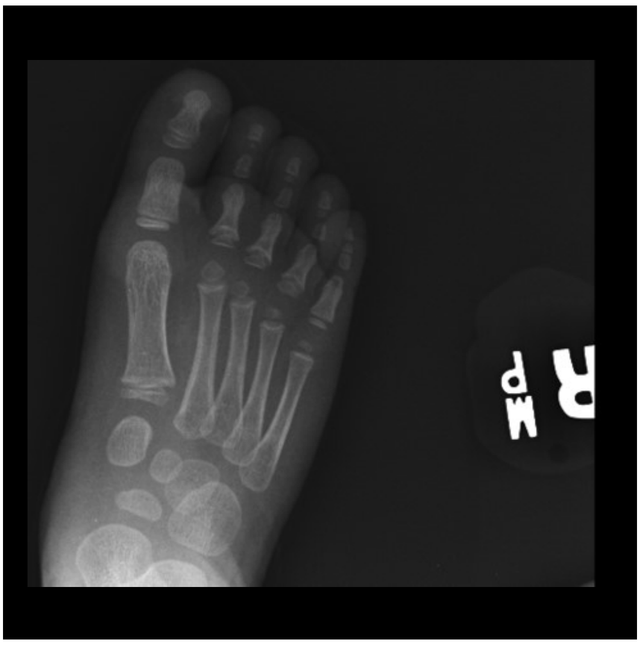

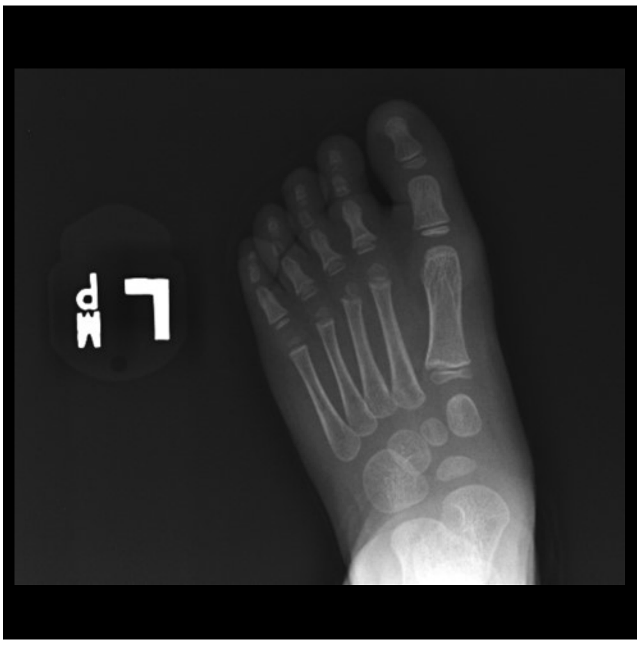

Case 3

Case 3

Circle | Square

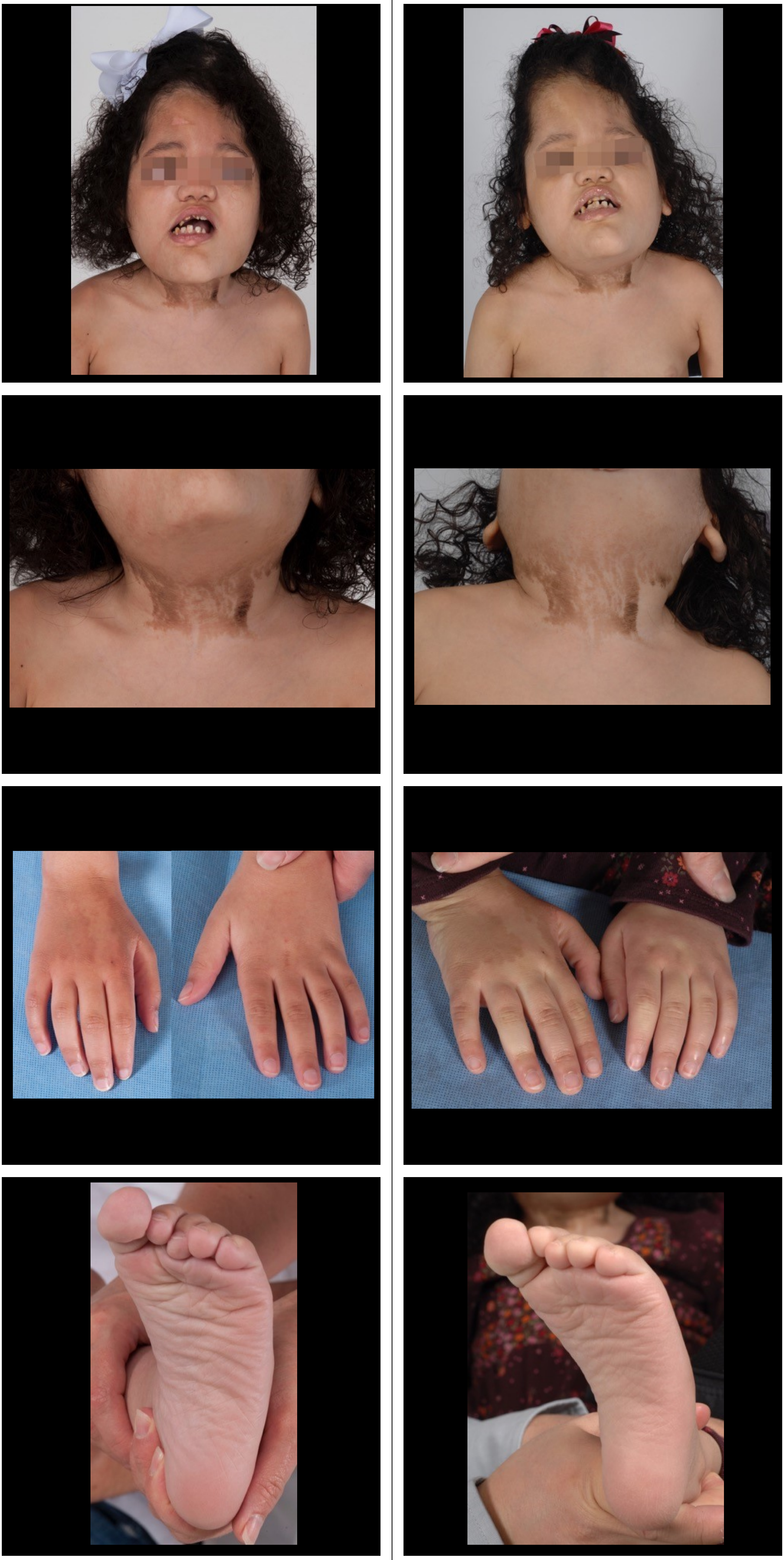

Case 3

Circle | Square

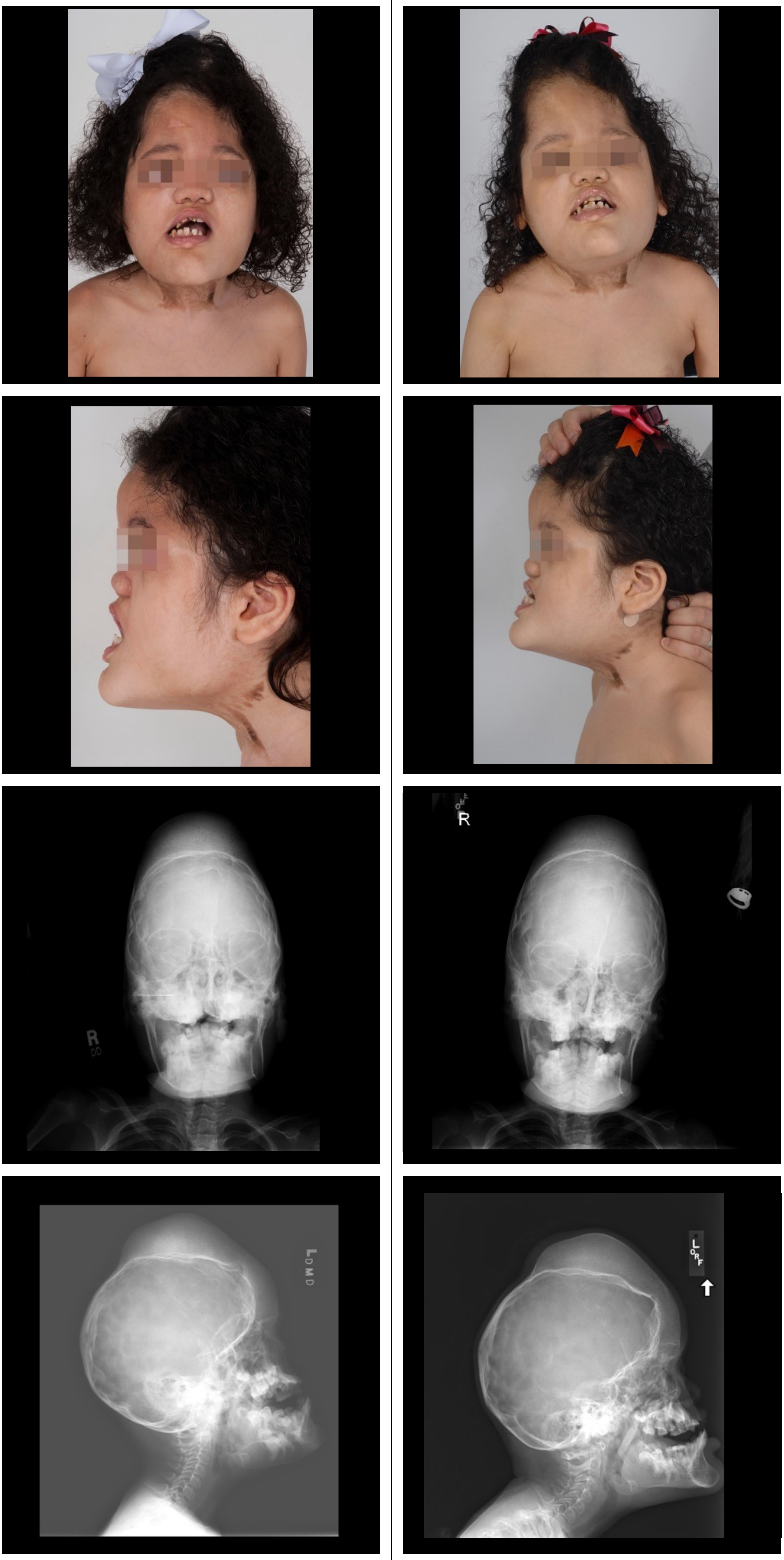

Case 3

Circle

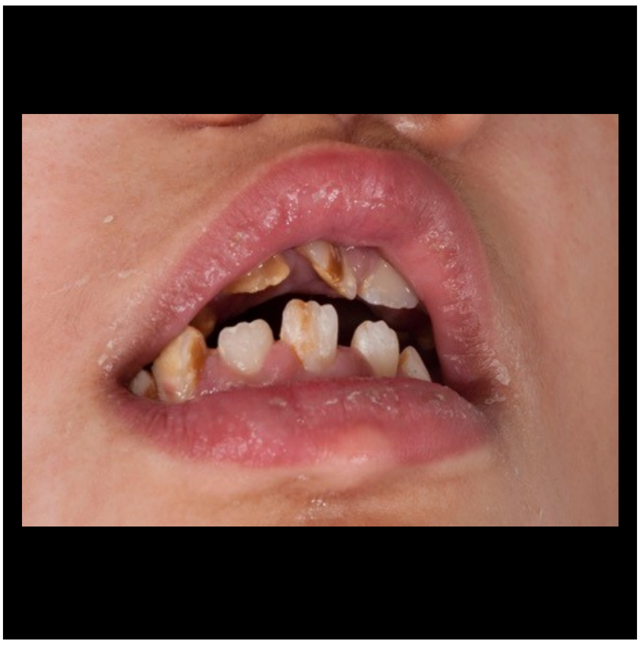

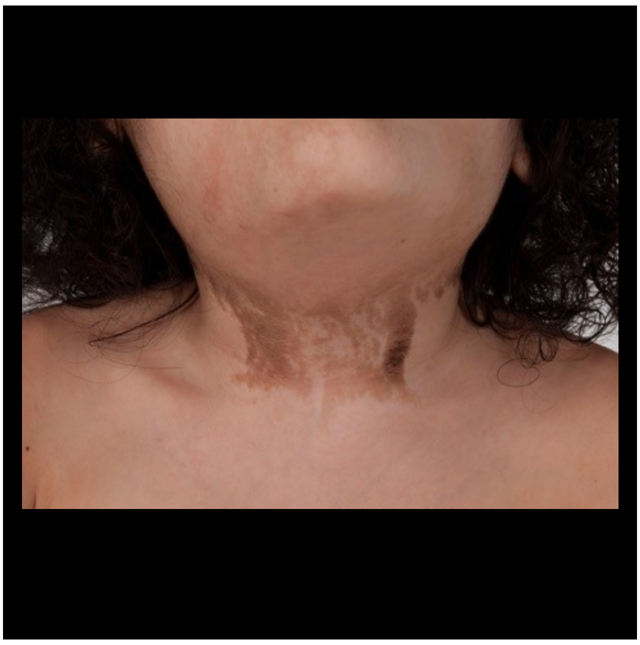

Square

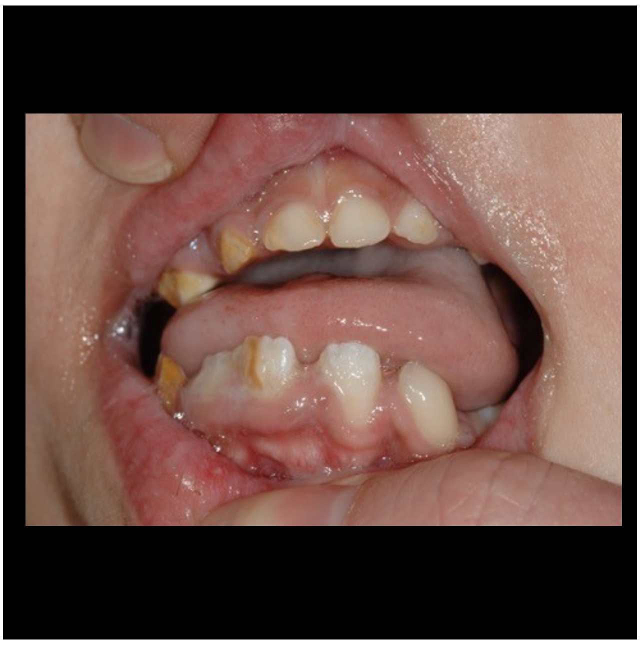

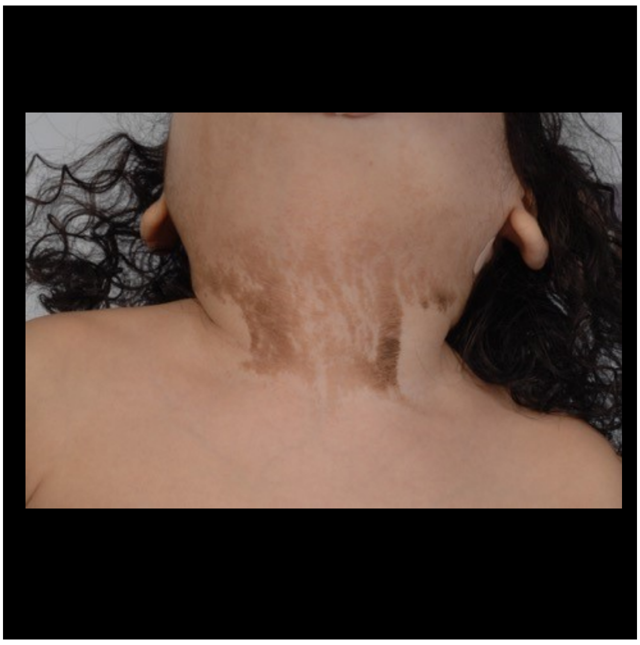

Case 3

Circle | Square

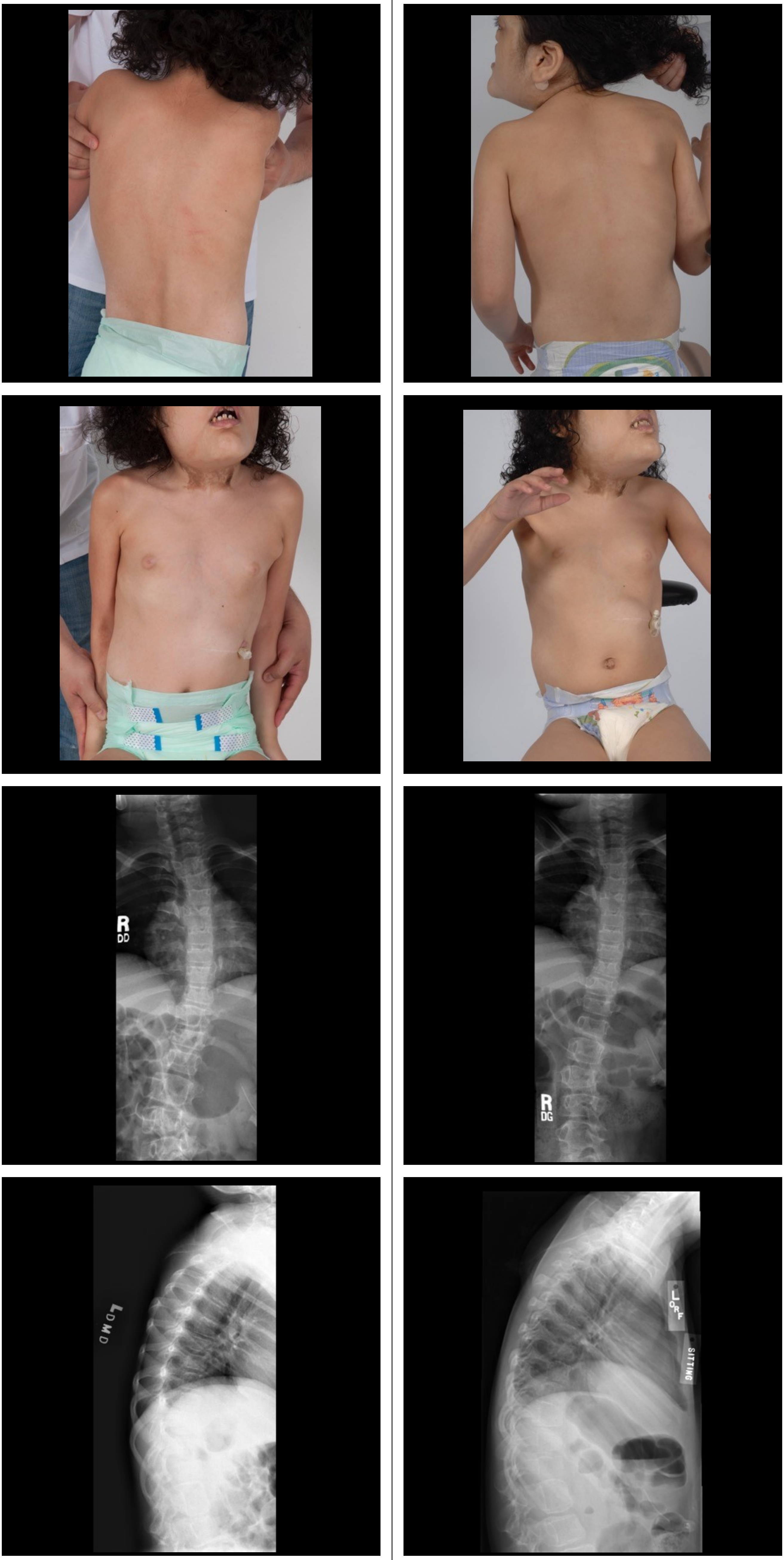

Case 3

Circle

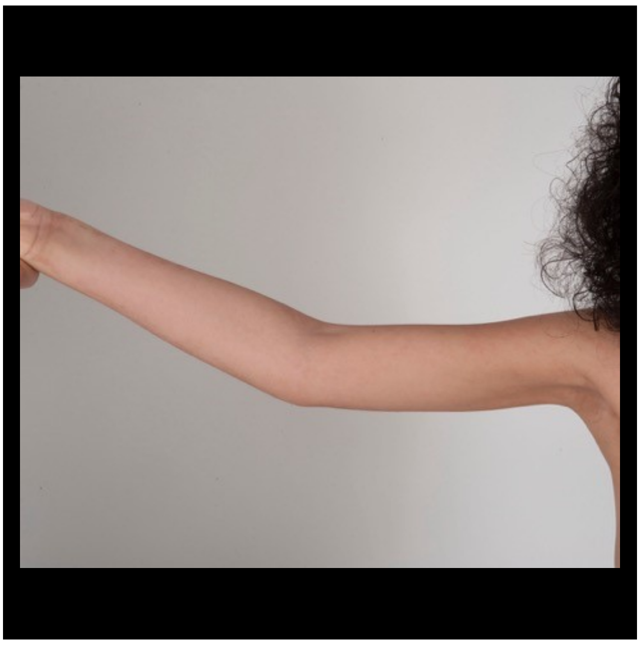

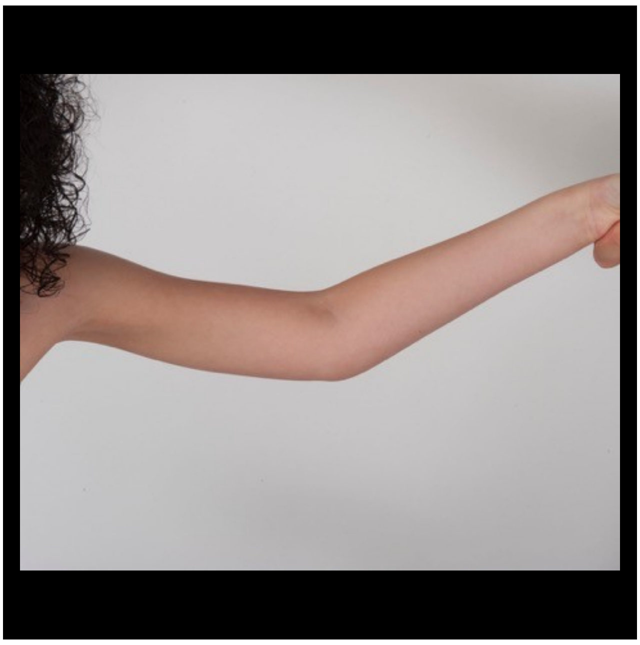

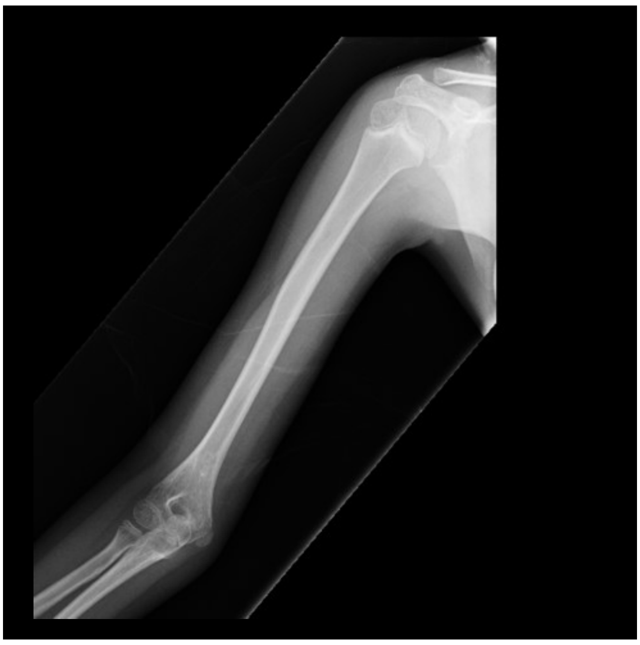

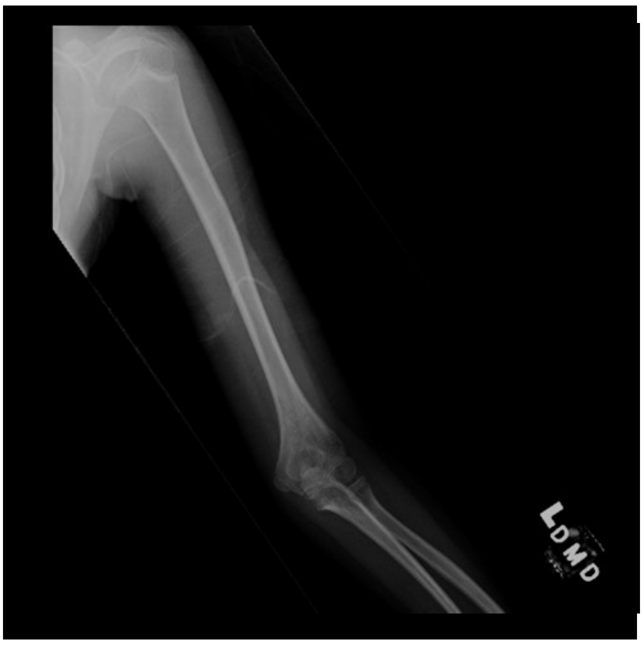

Square

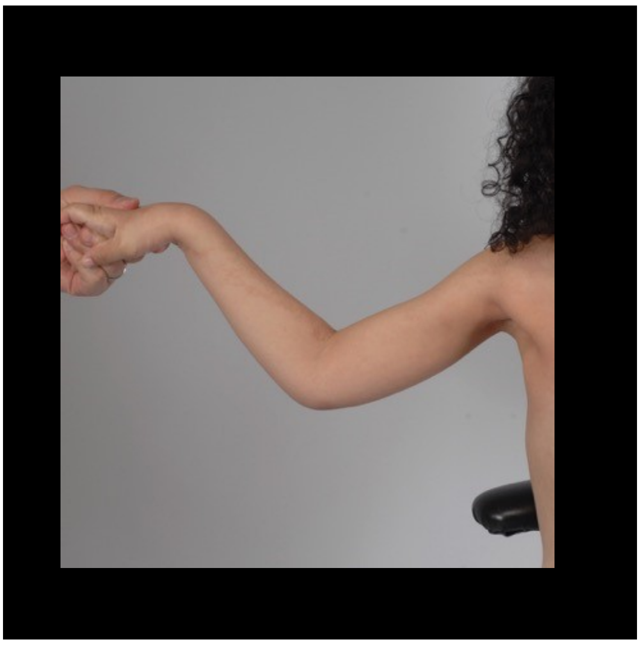

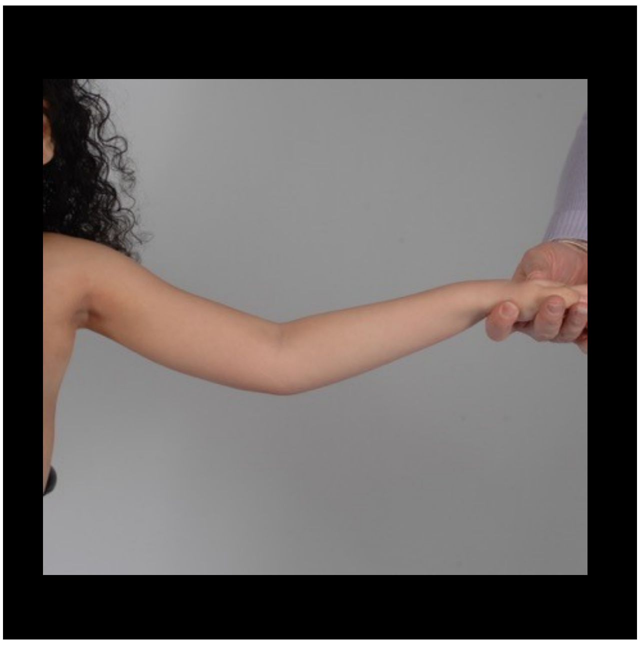

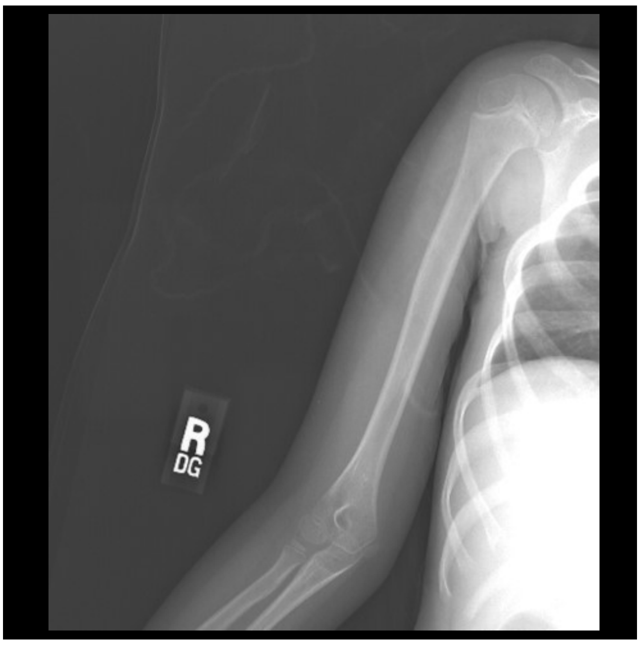

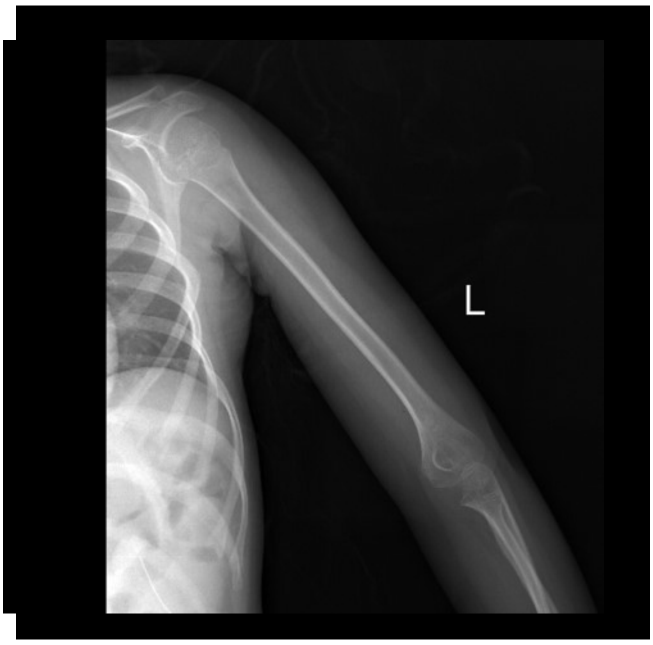

Case 3

Circle

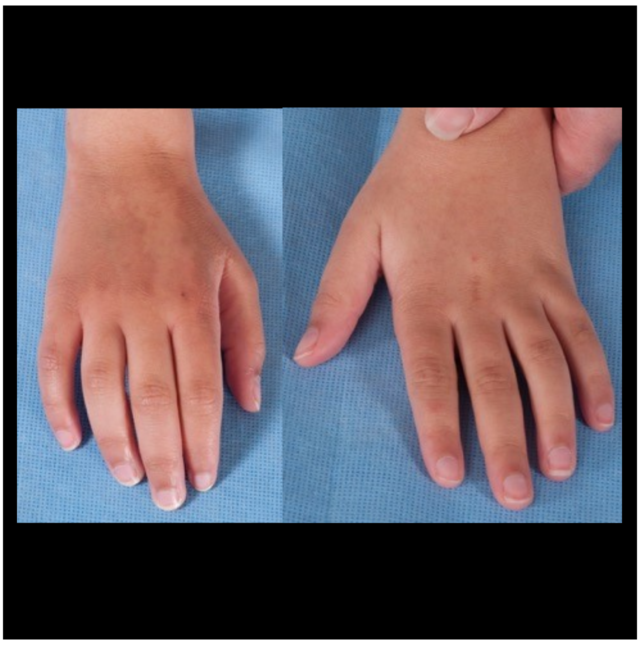

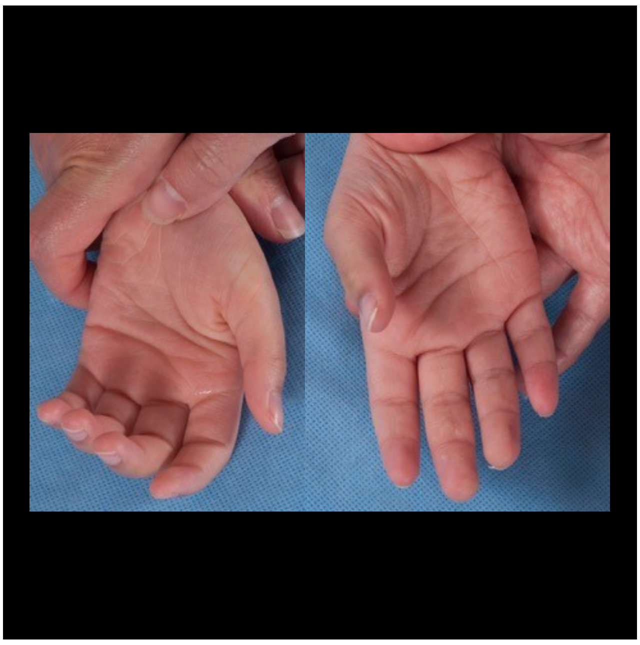

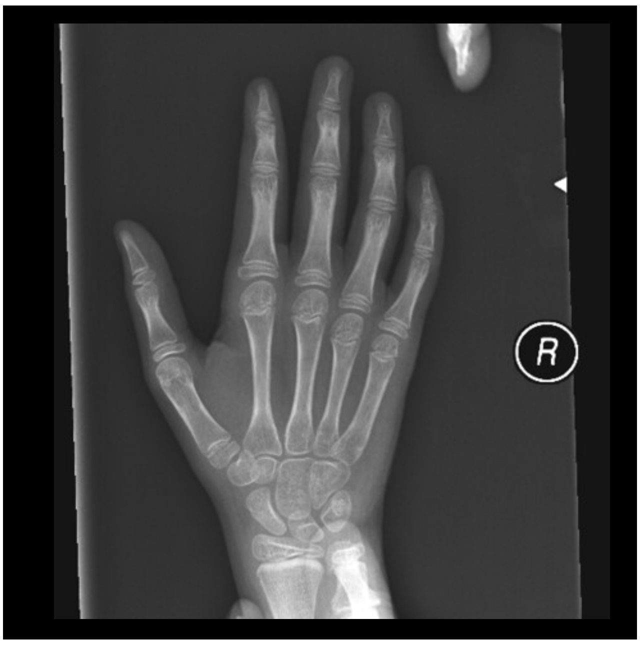

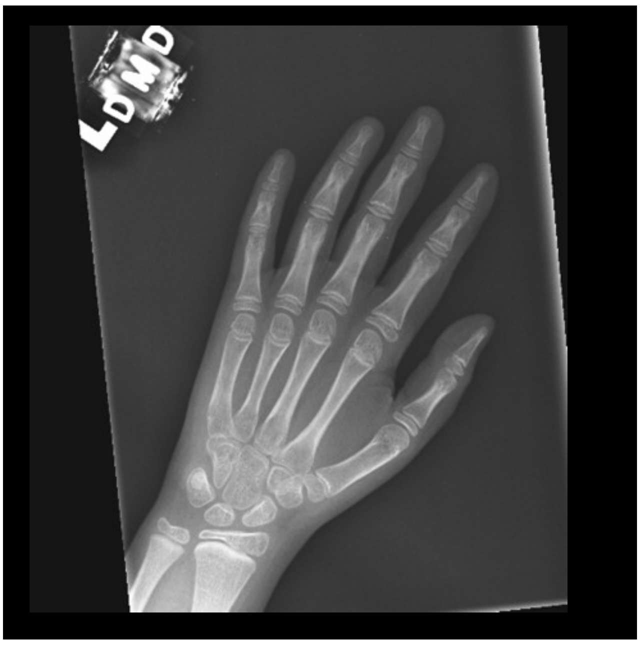

Square

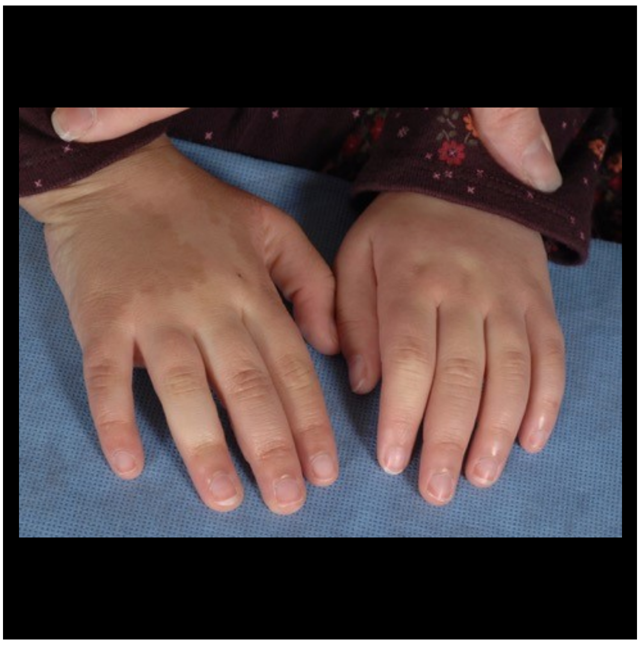

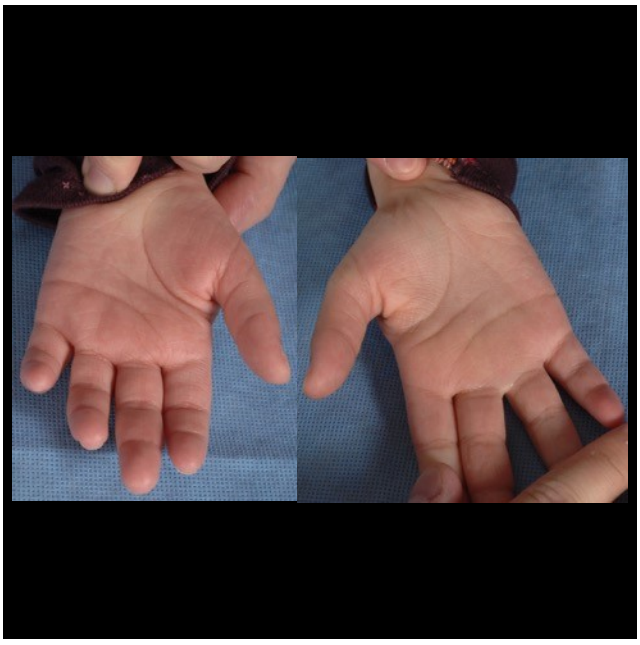

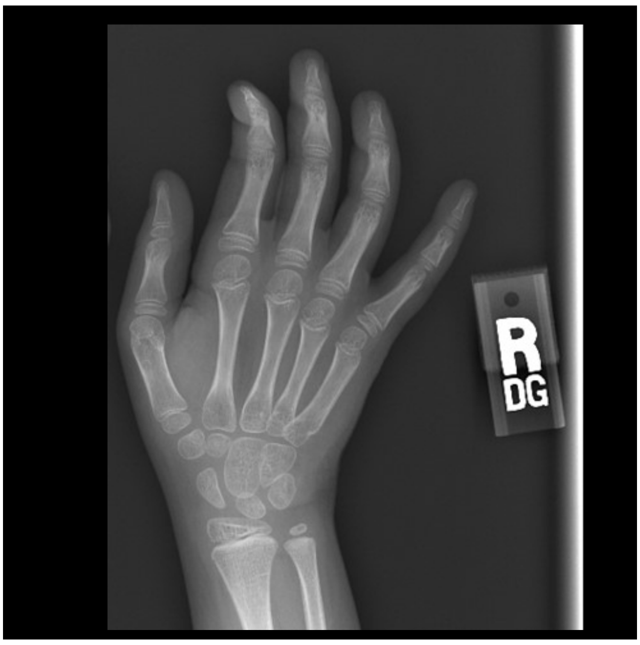

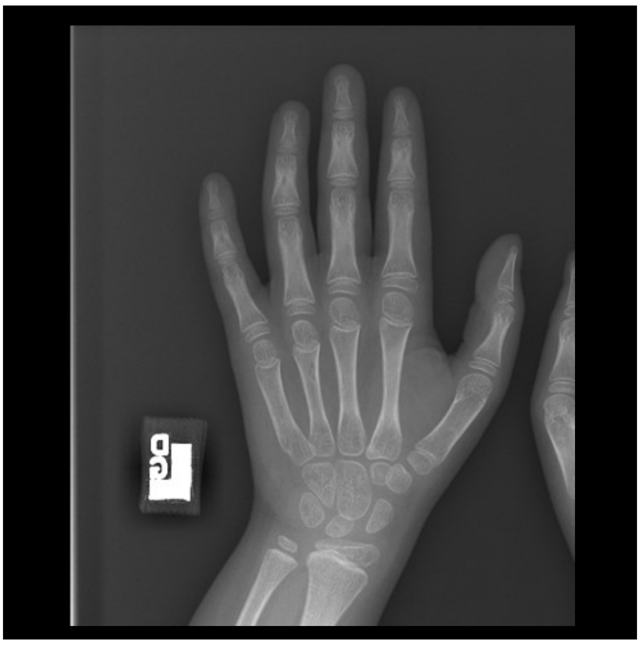

Case 3

Circle

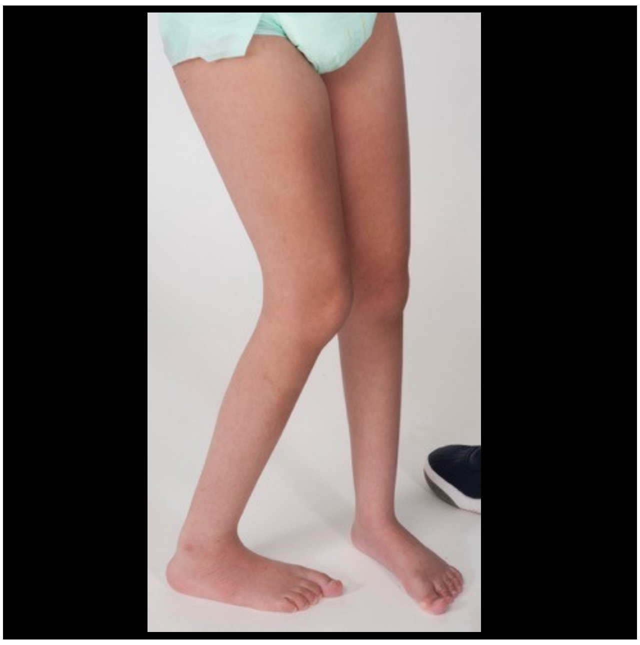

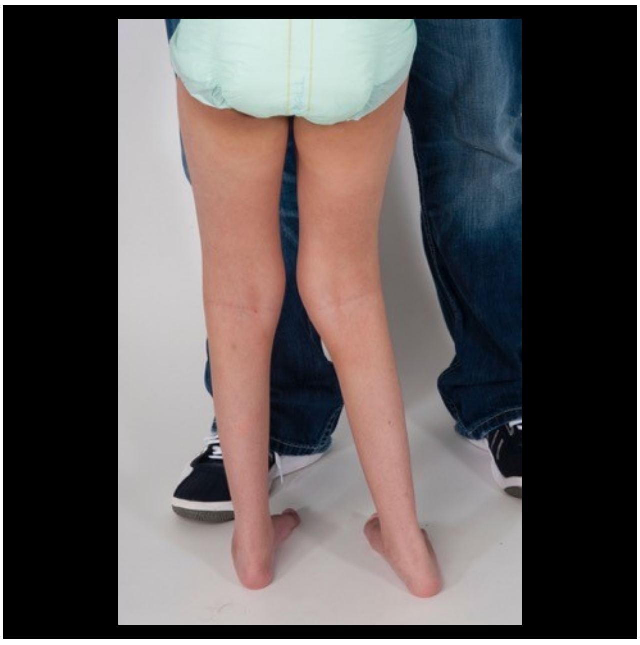

Square

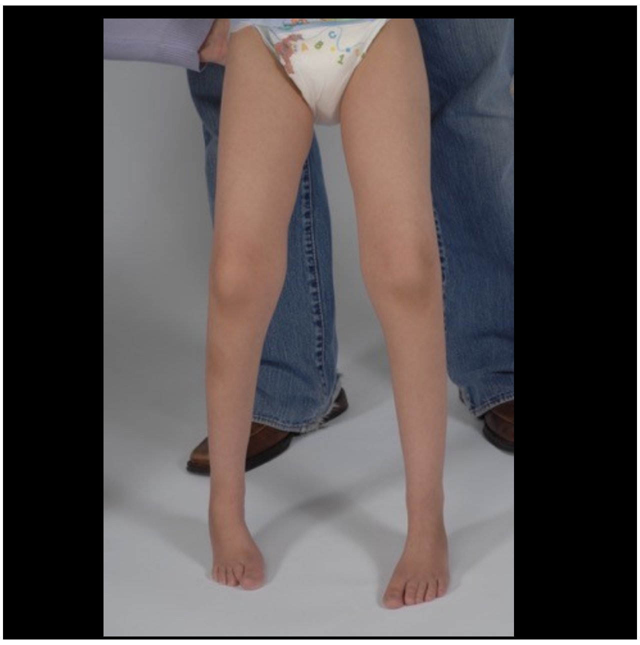

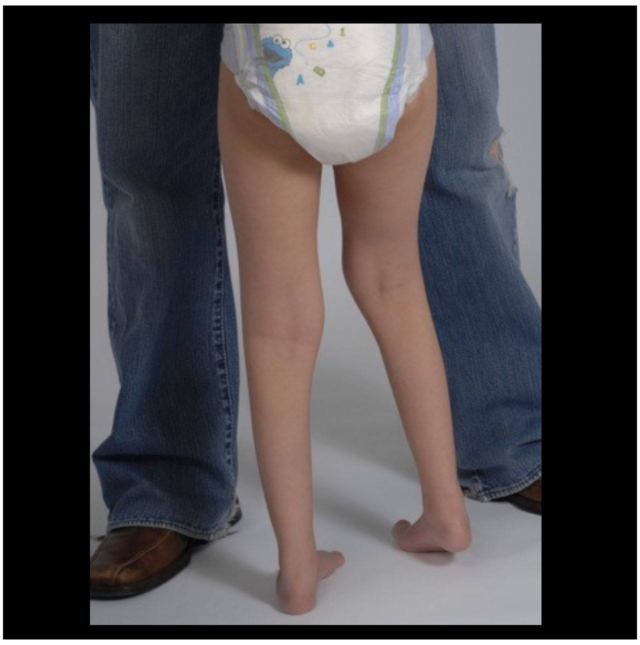

Case 3

Circle

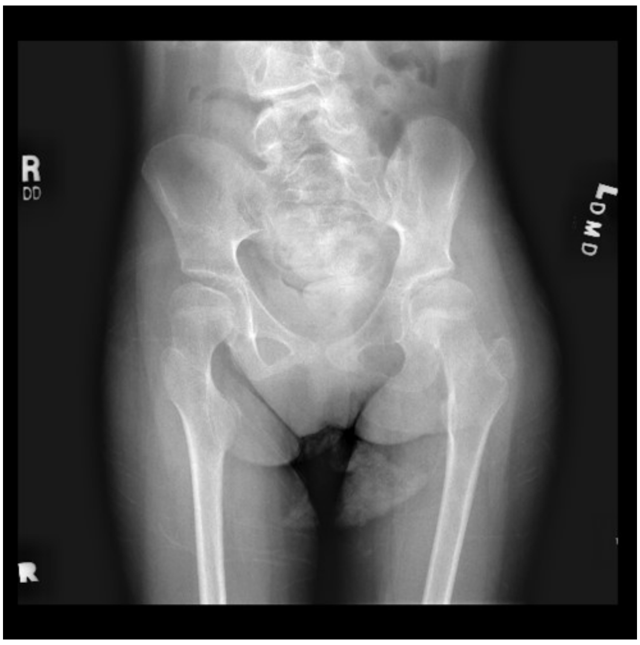

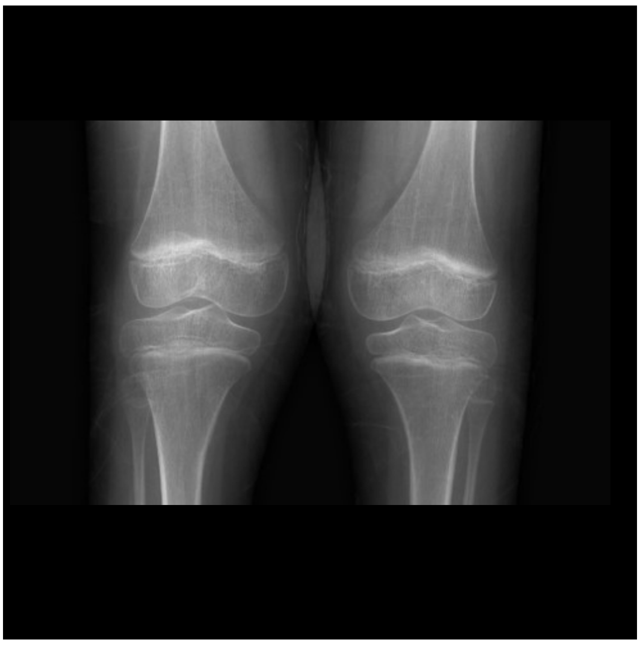

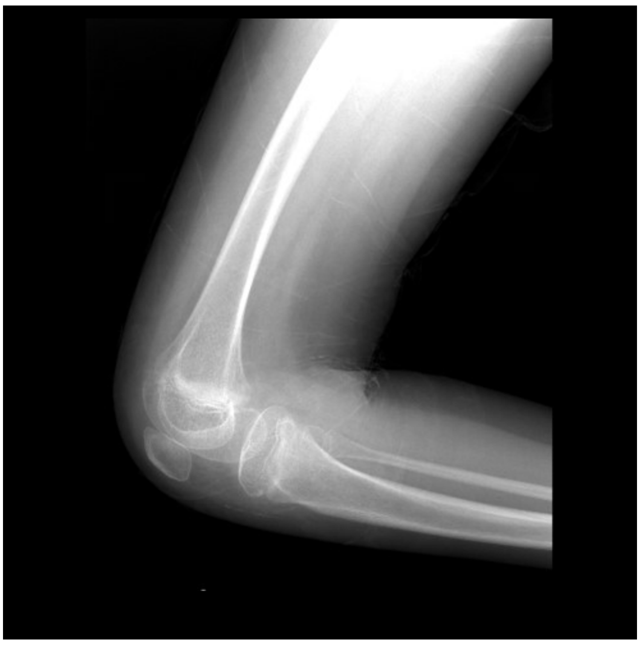

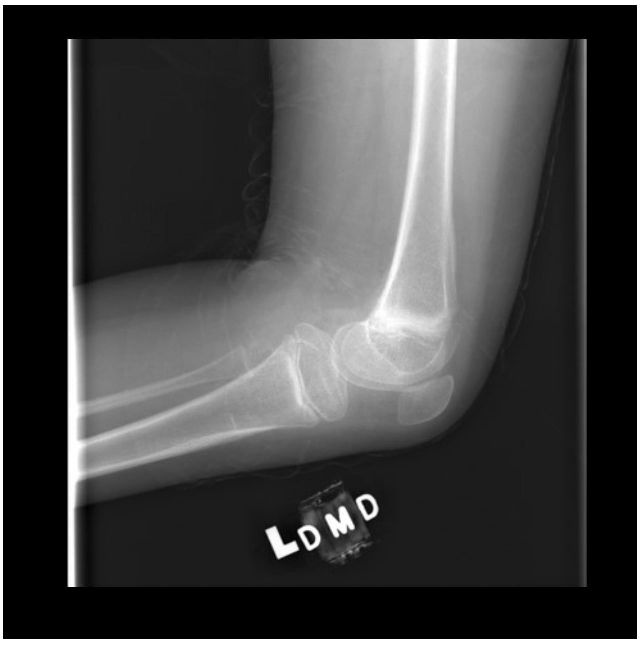

Square

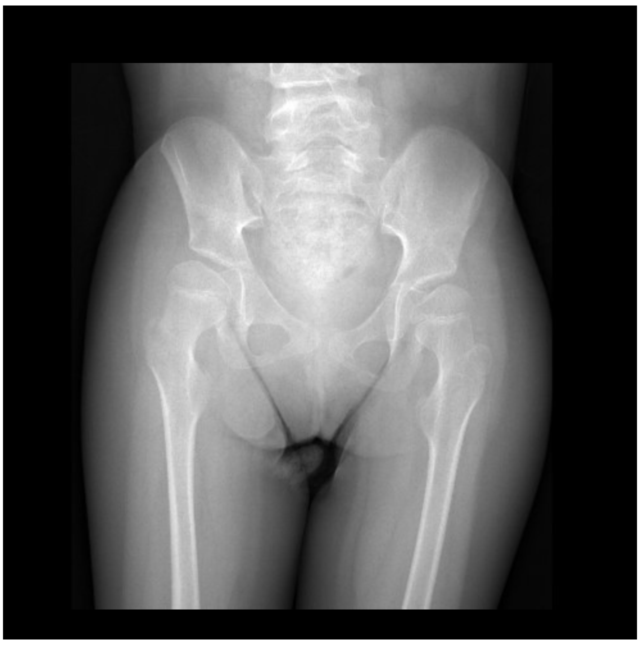

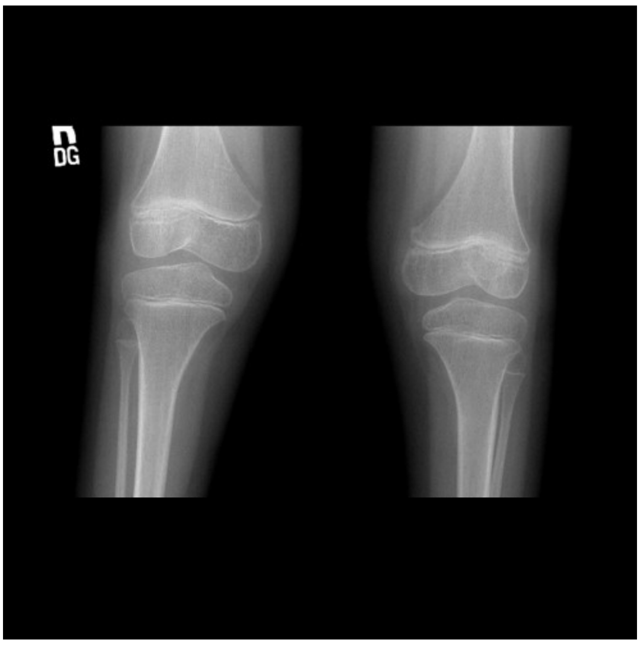

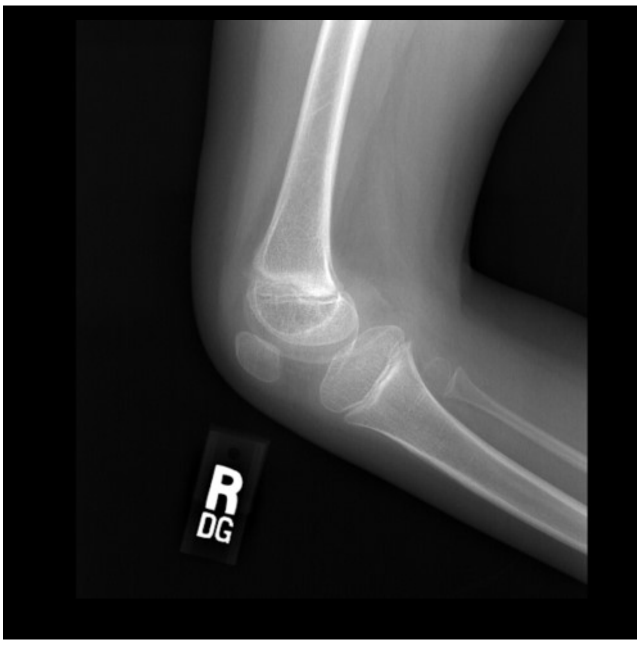

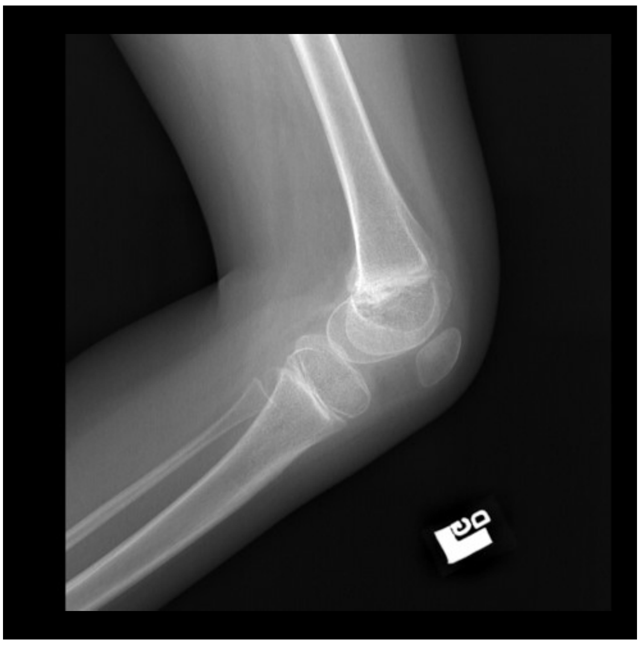

Case 3

Circle

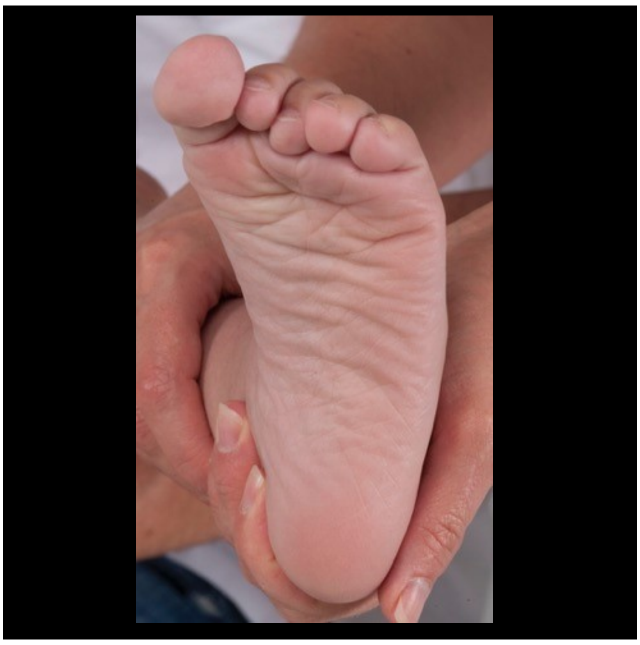

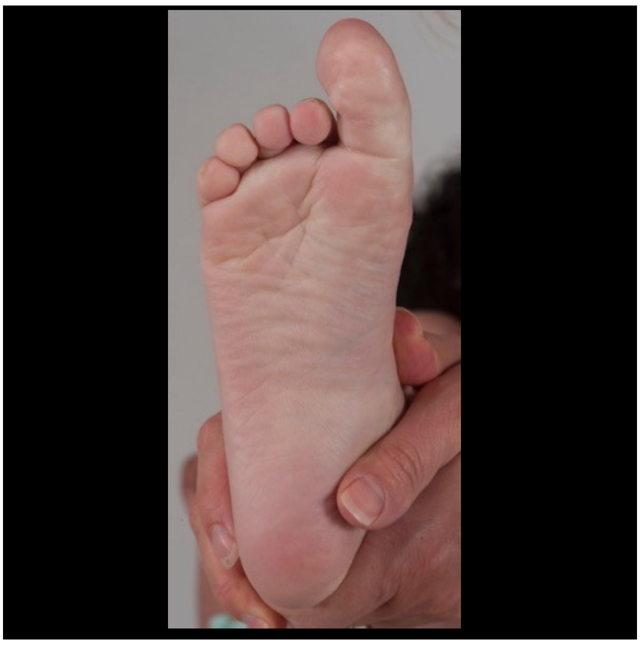

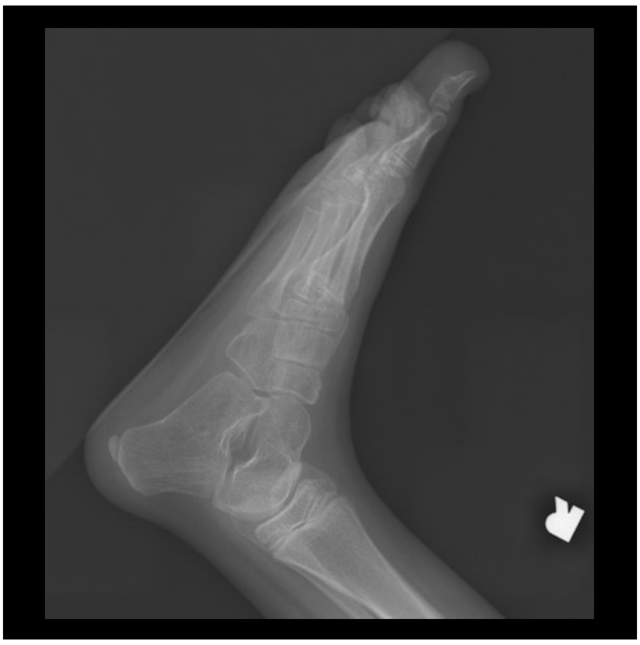

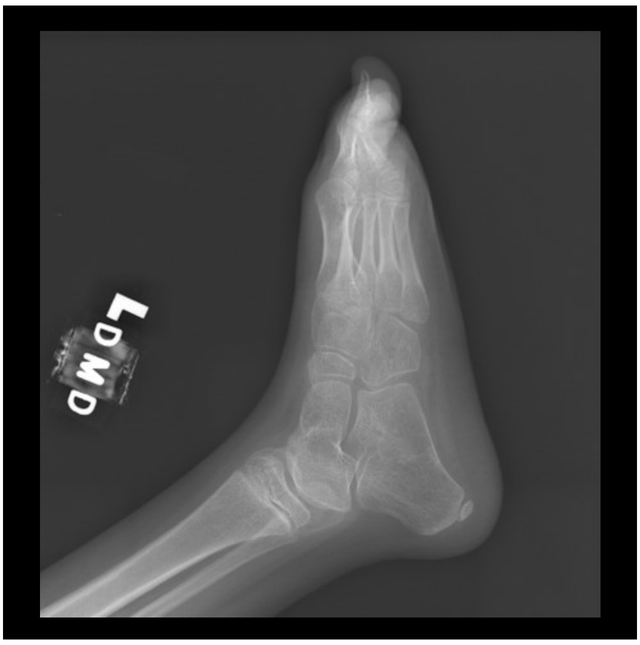

Square

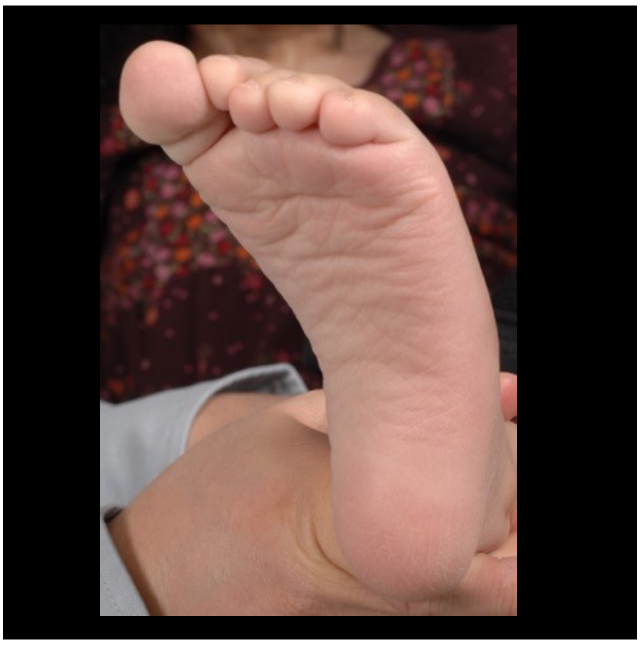

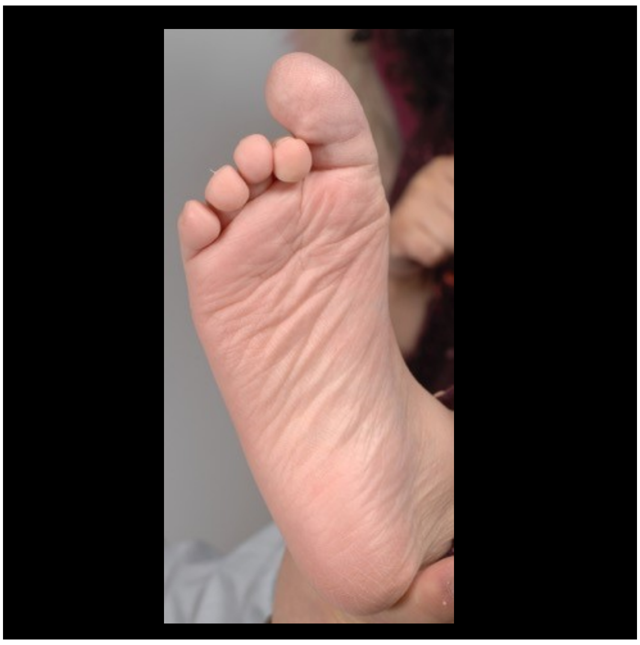

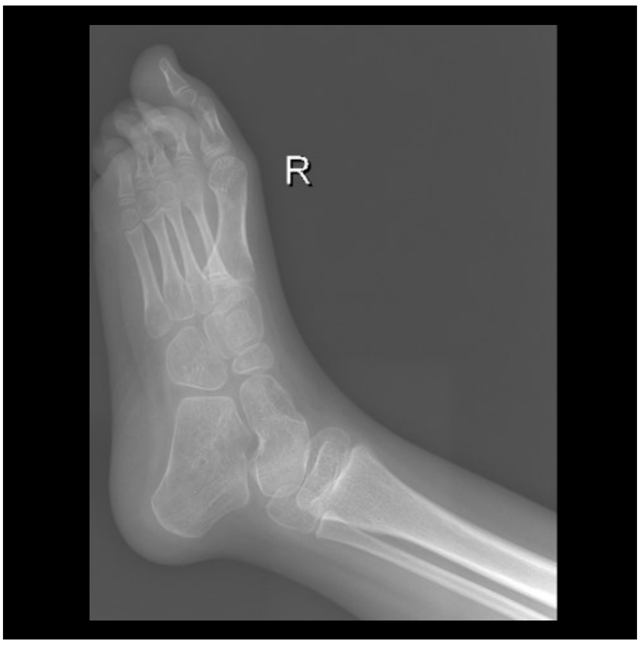

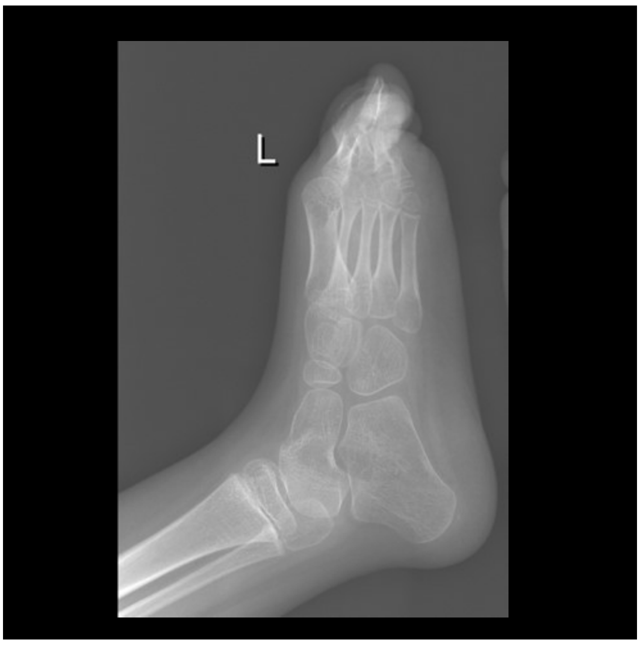

Case 4

Case 4

Circle

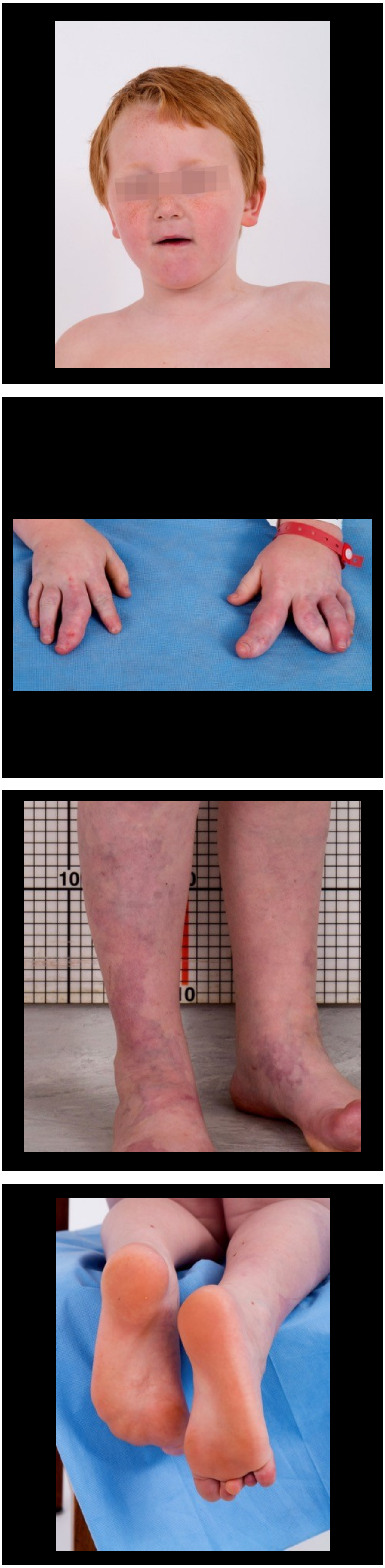

Square

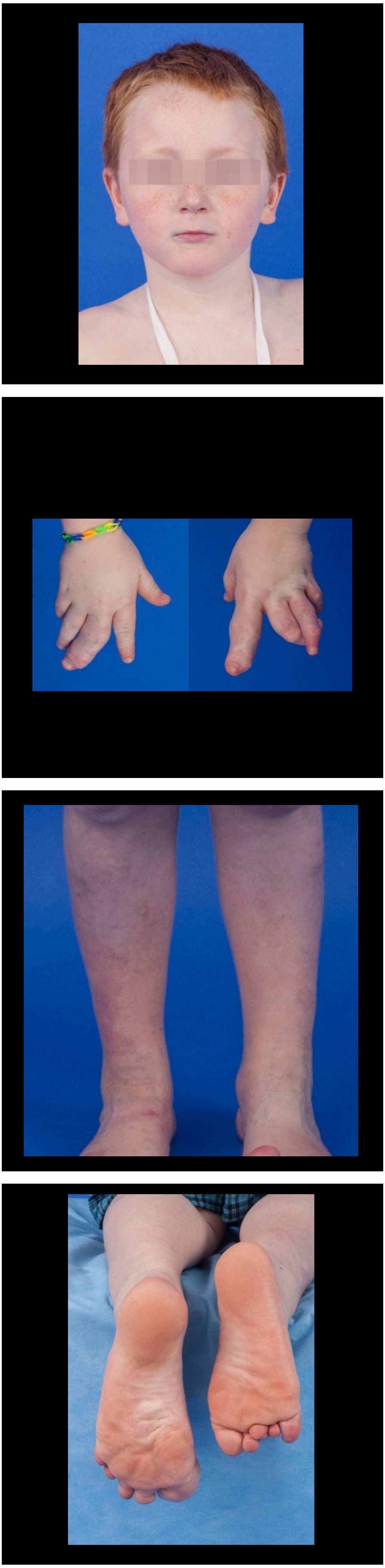

Case 4

Circle

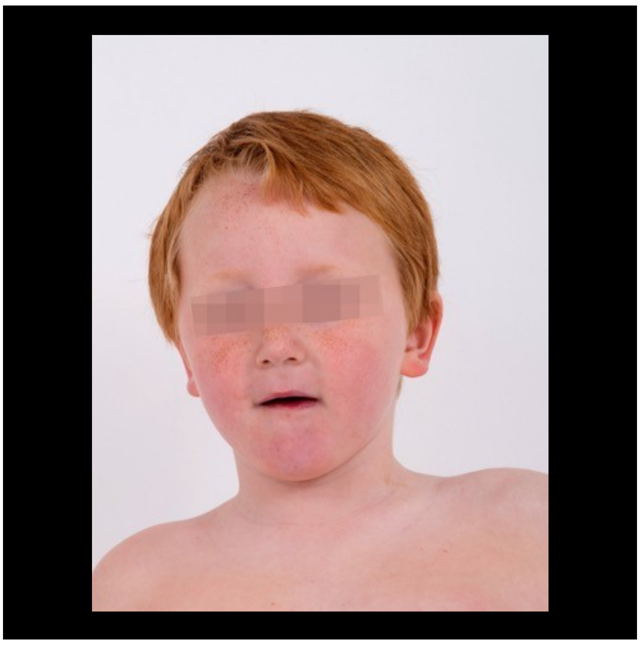

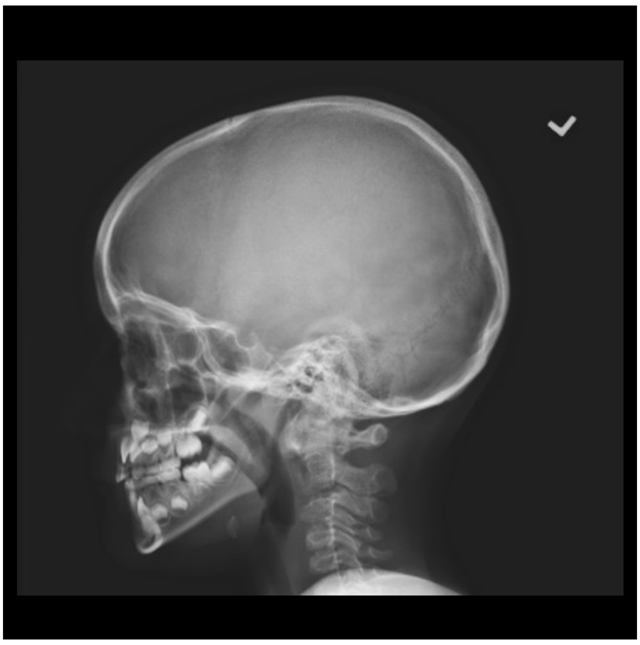

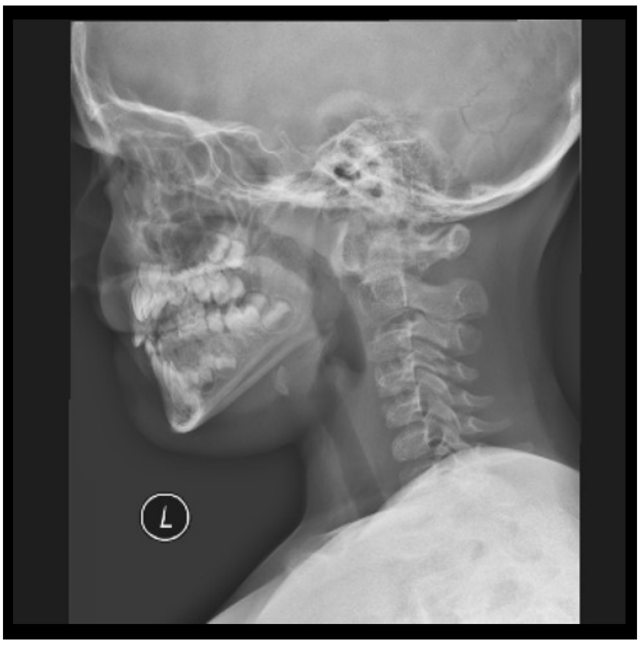

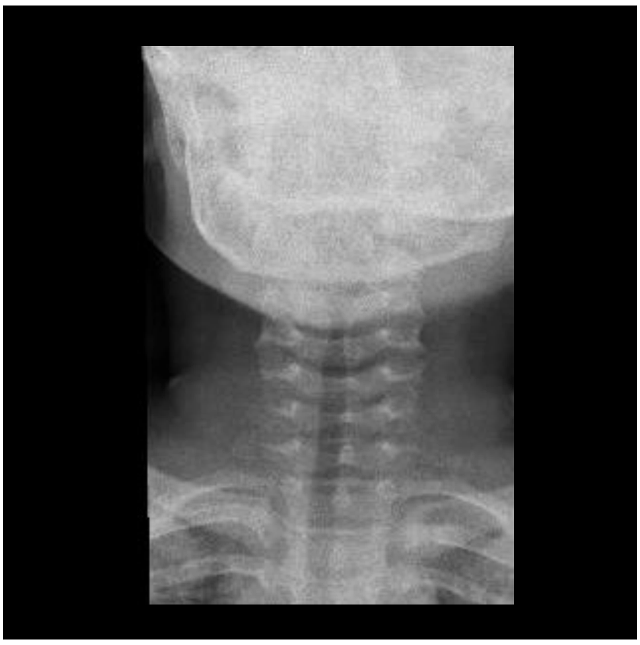

Square

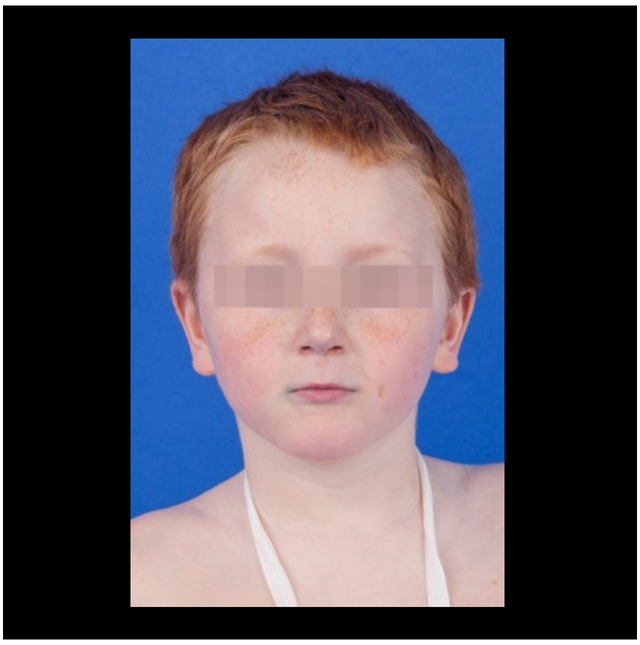

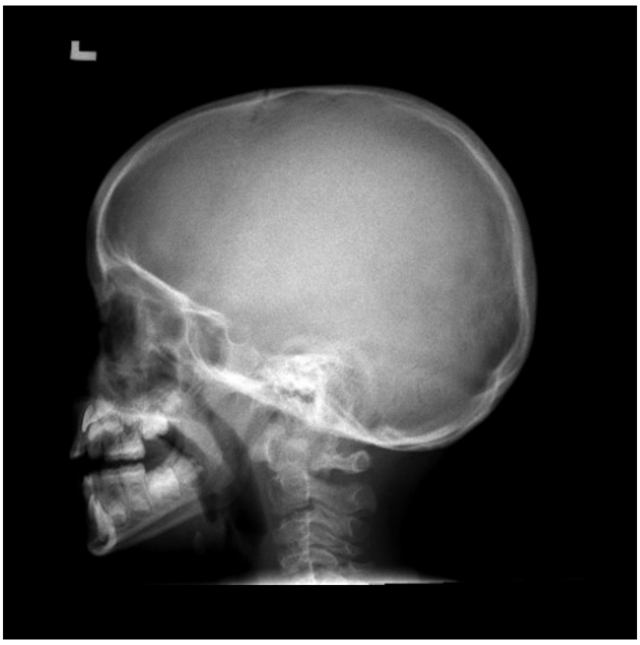

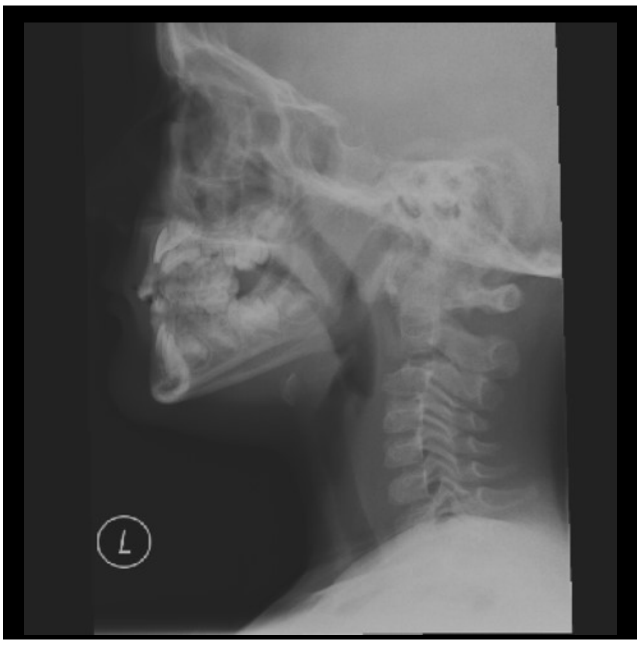

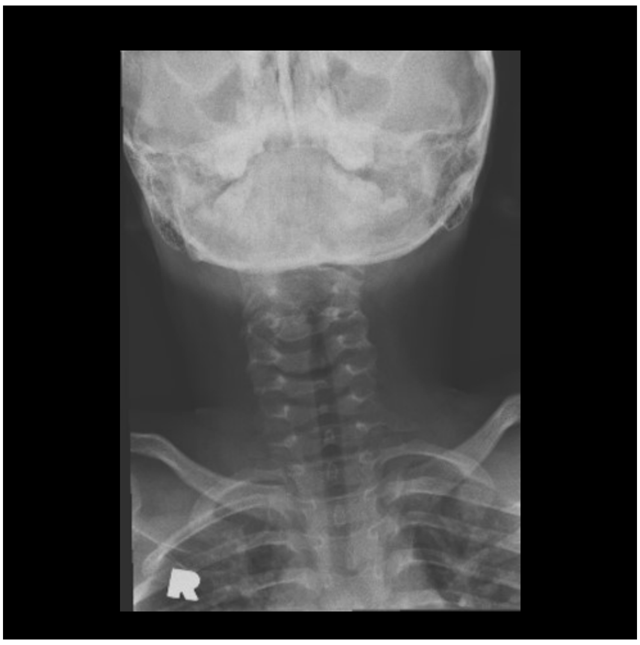

Case 4

Circle

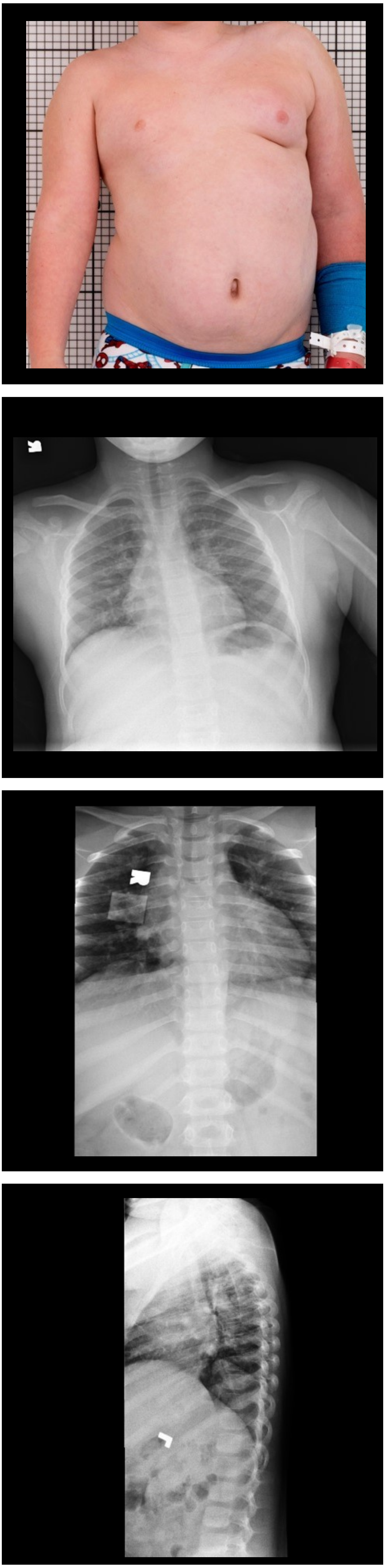

Square

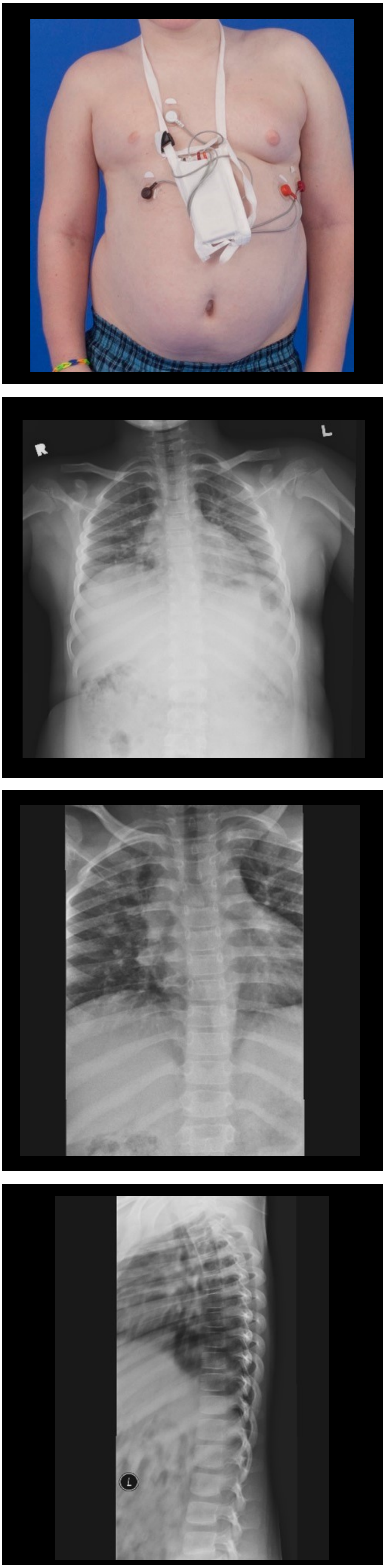

Case 4

Circle

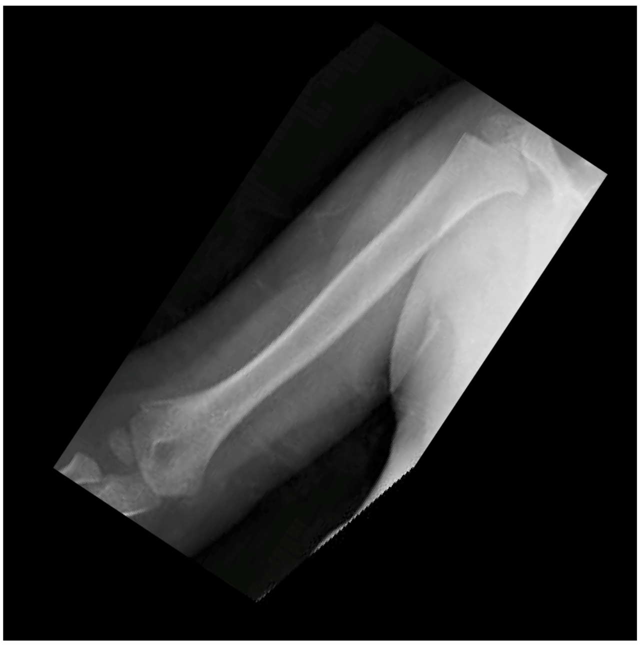

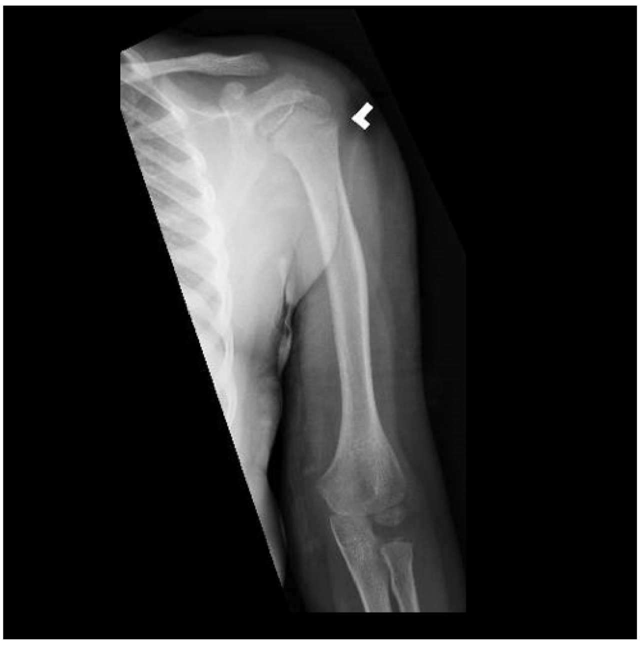

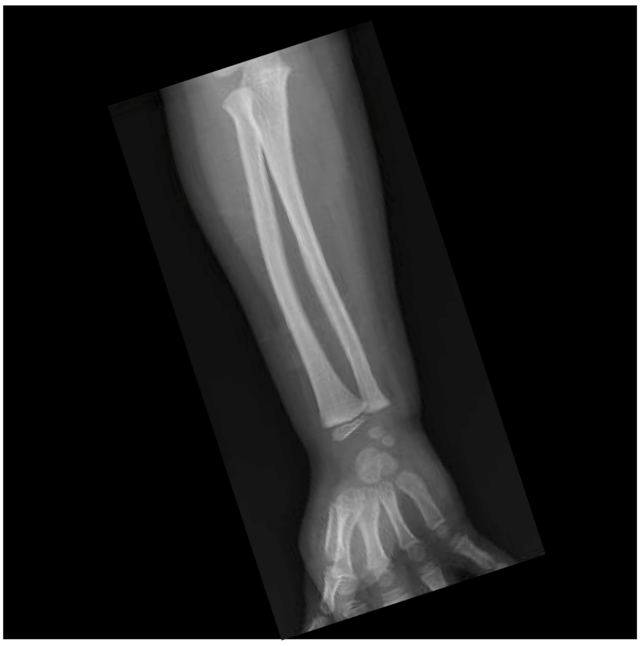

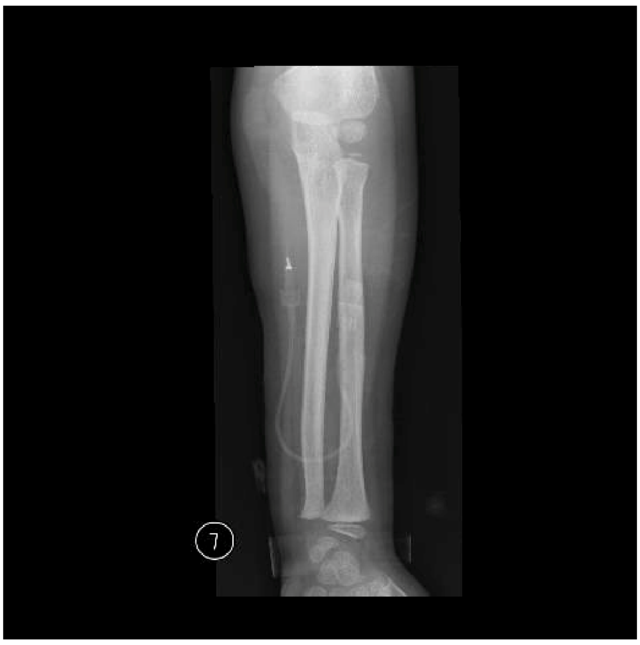

Square

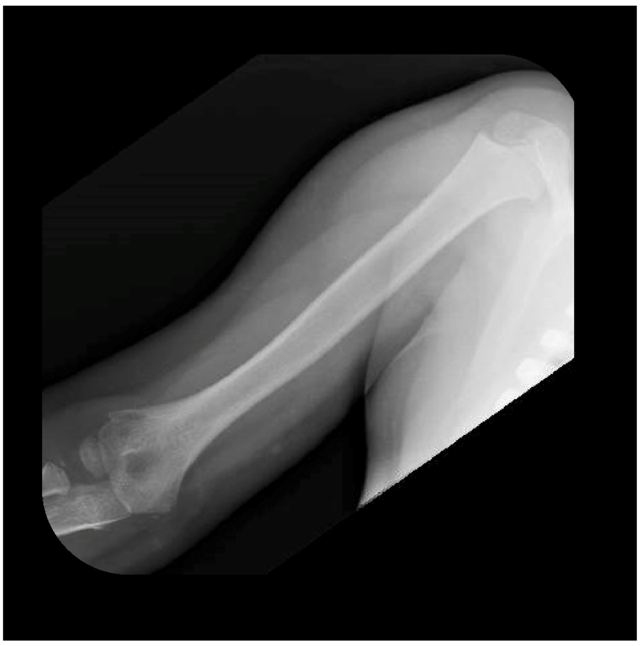

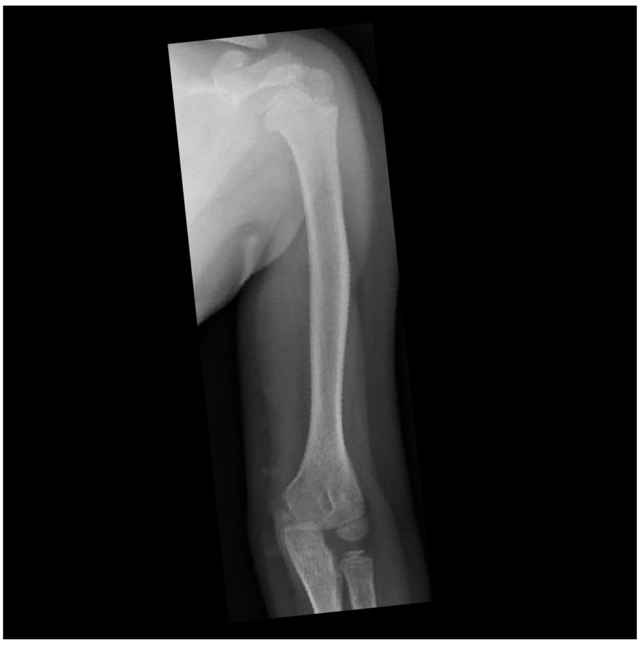

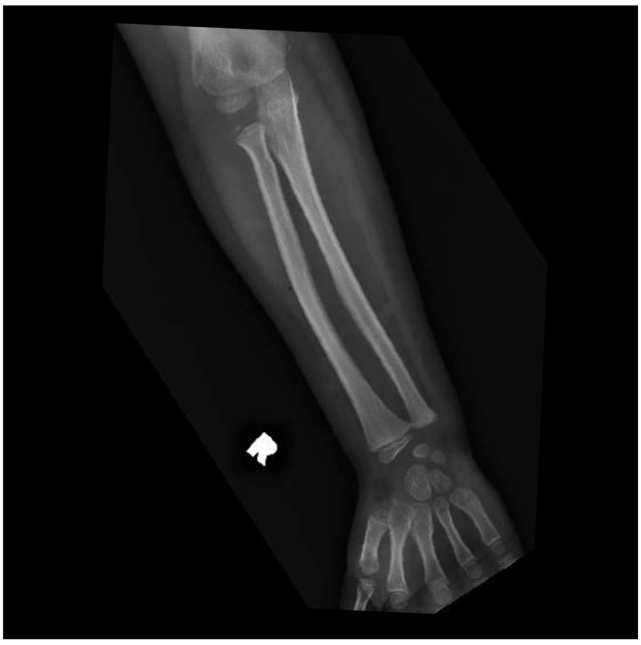

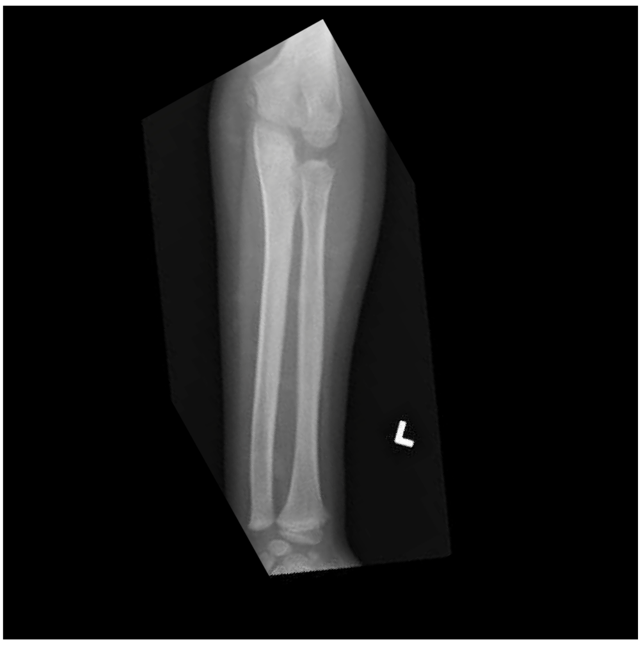

Case 4

Circle | Square

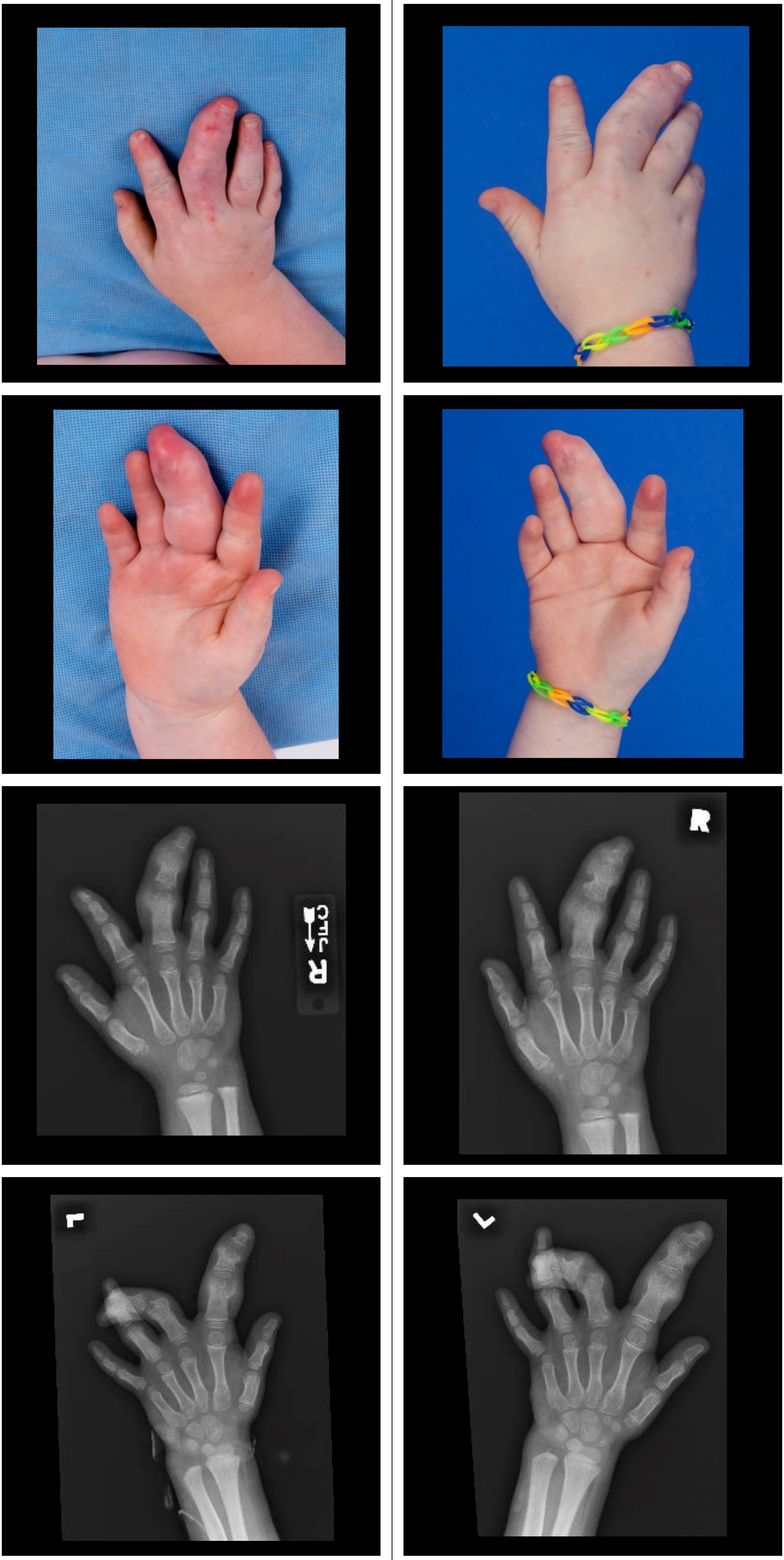

Case 4

Circle | Square

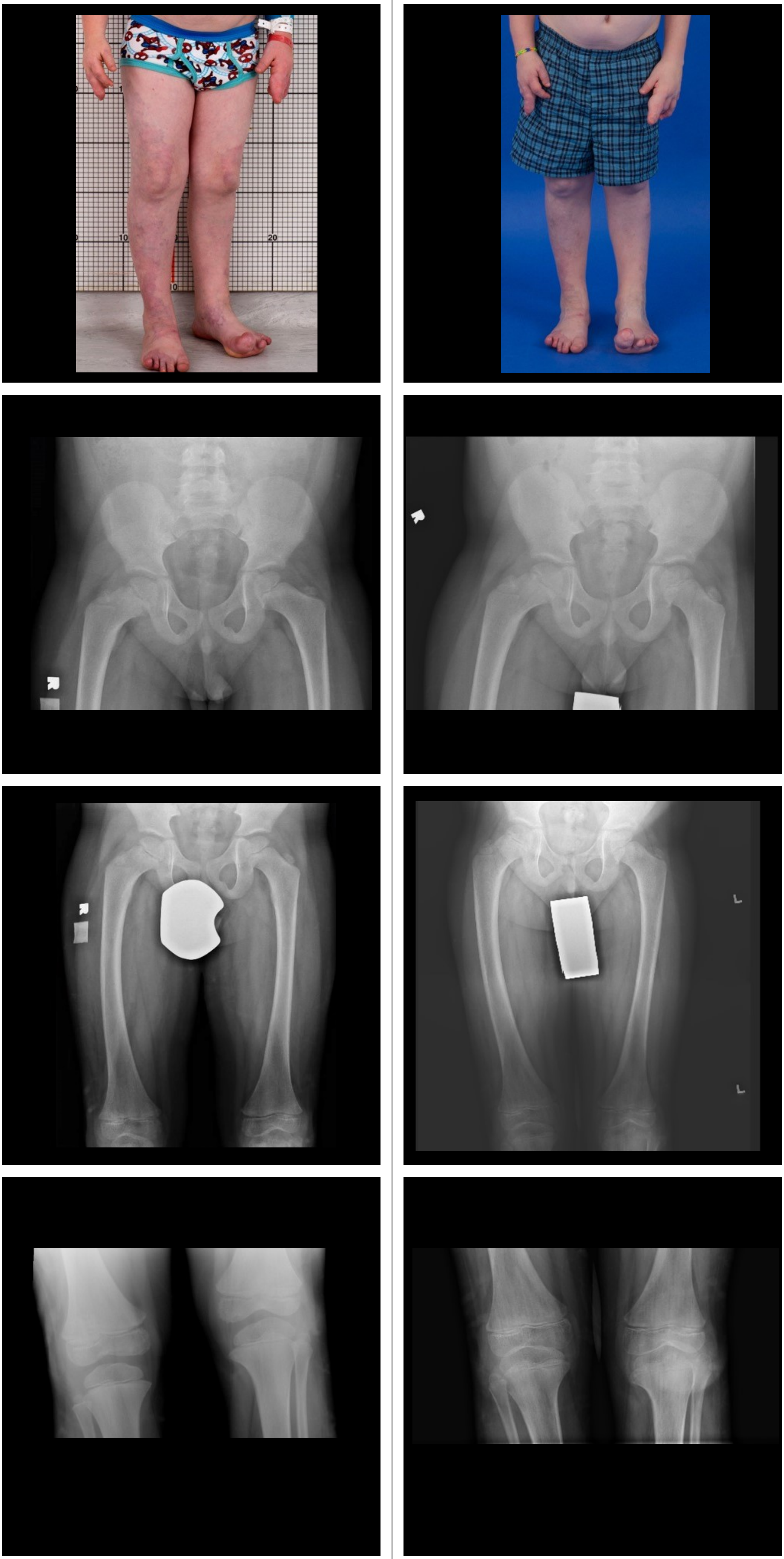

Case 4

Circle

Square

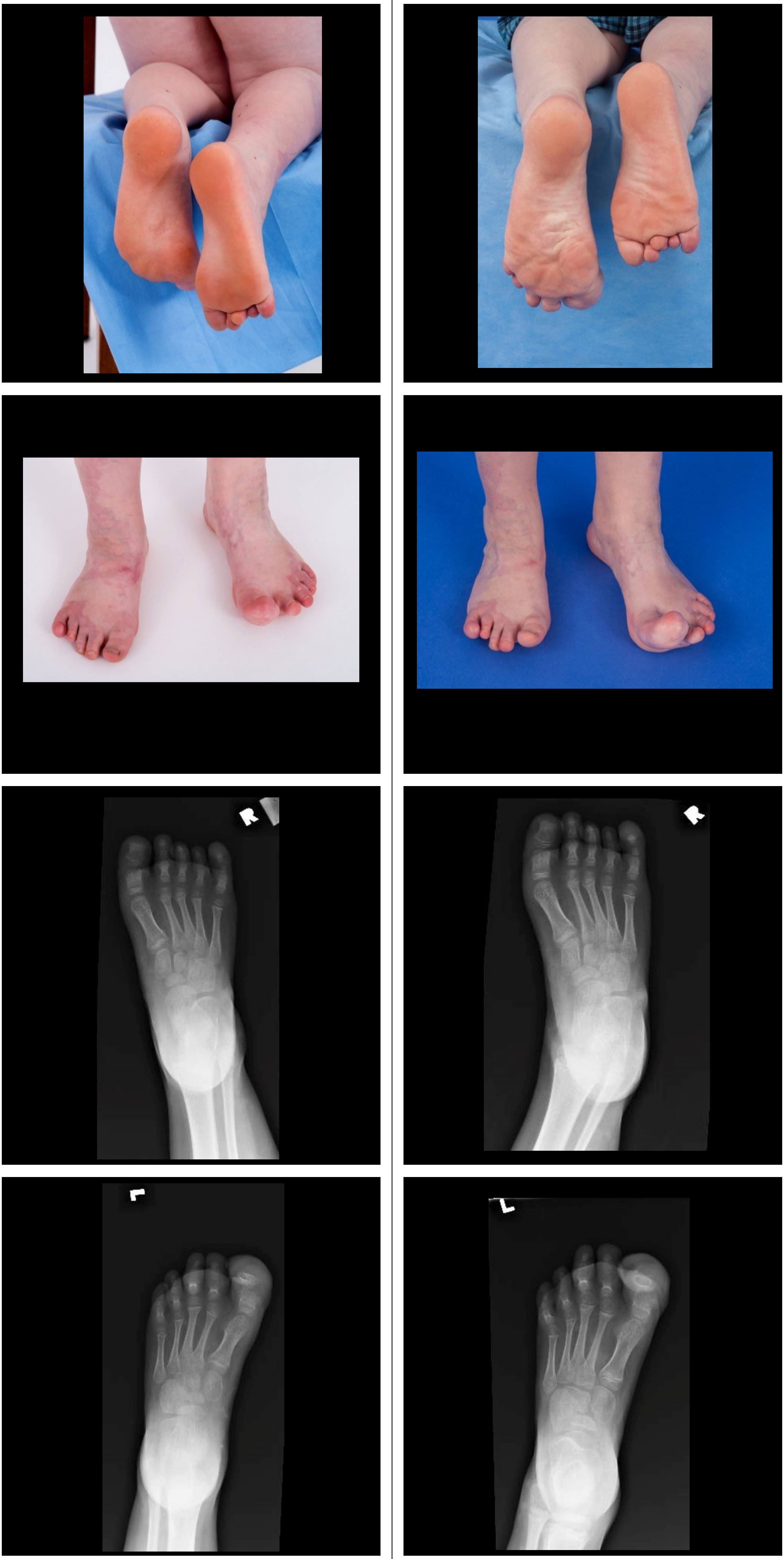

Case 5

Case 5

Circle | Square

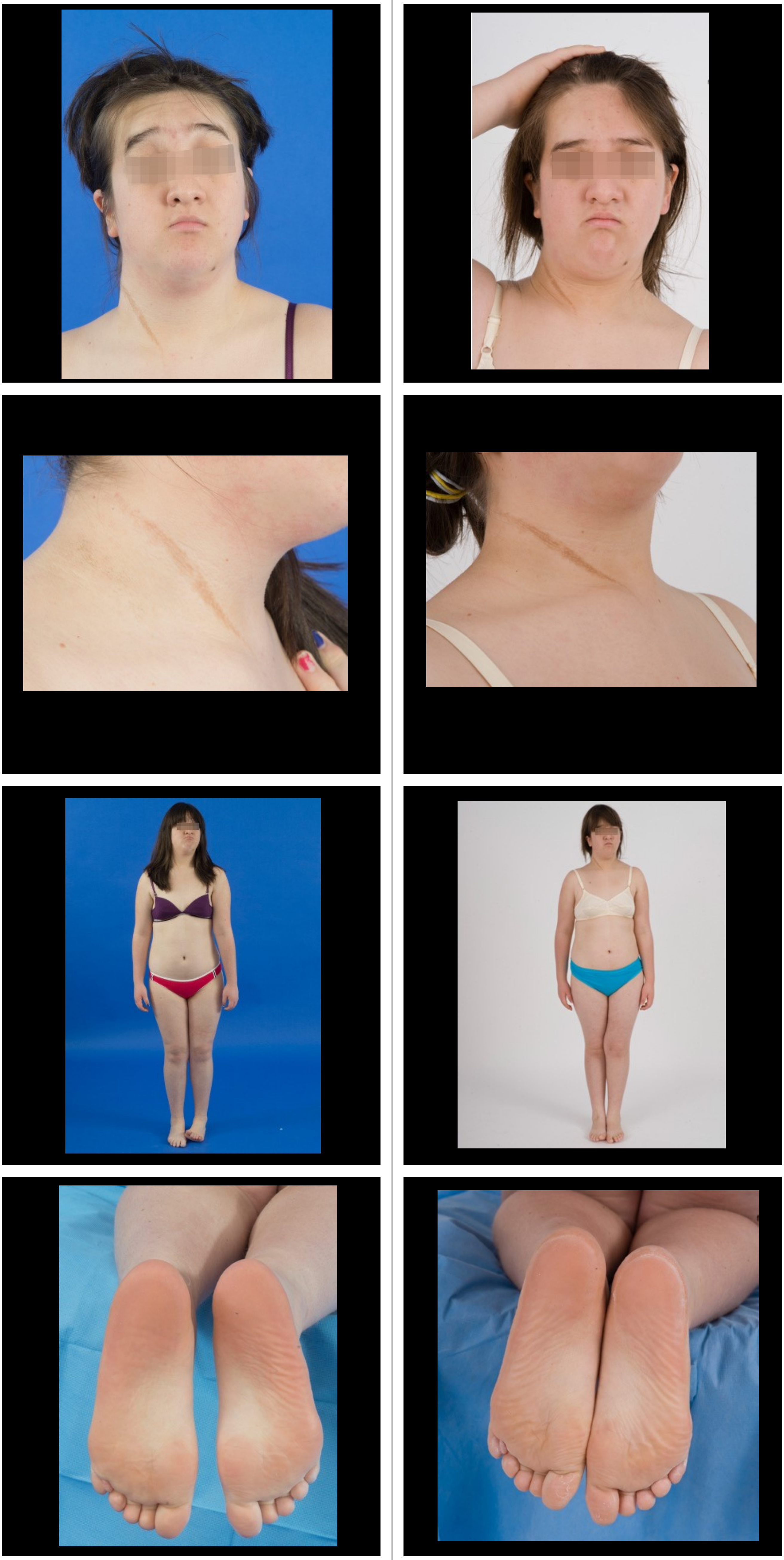

Case 5

Circle | Square

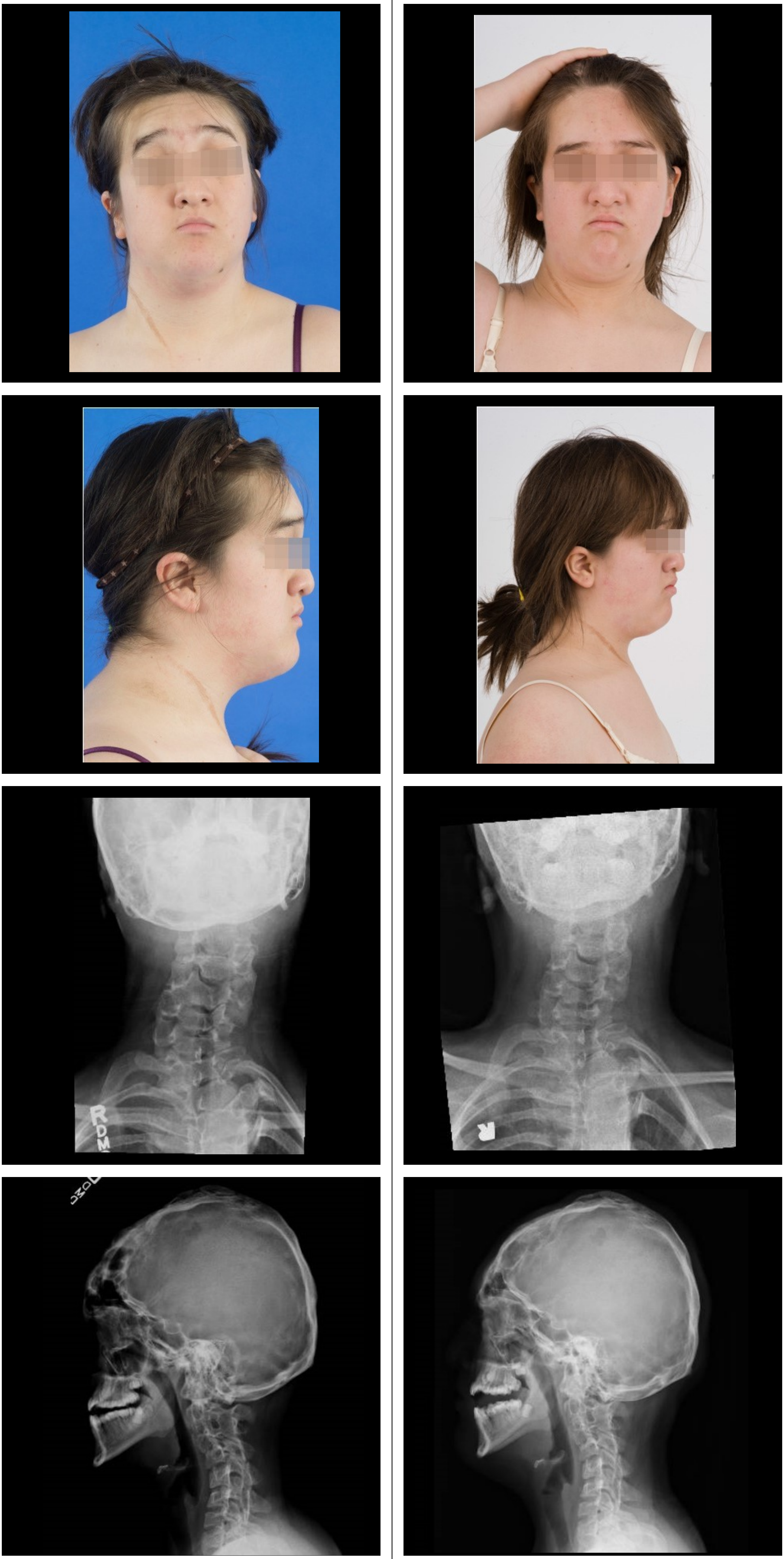

Case 5

Circle

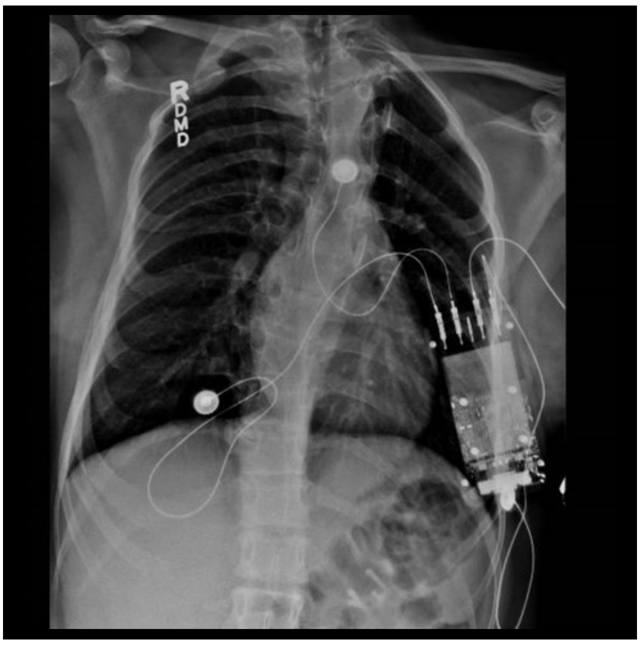

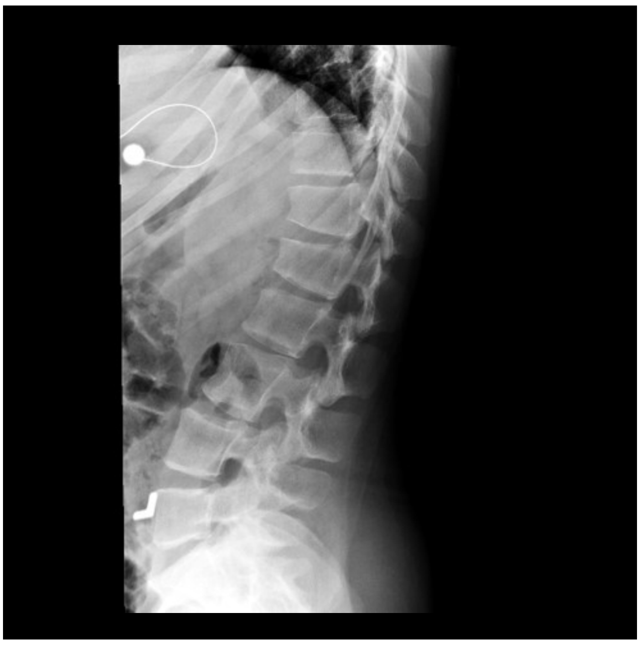

Square

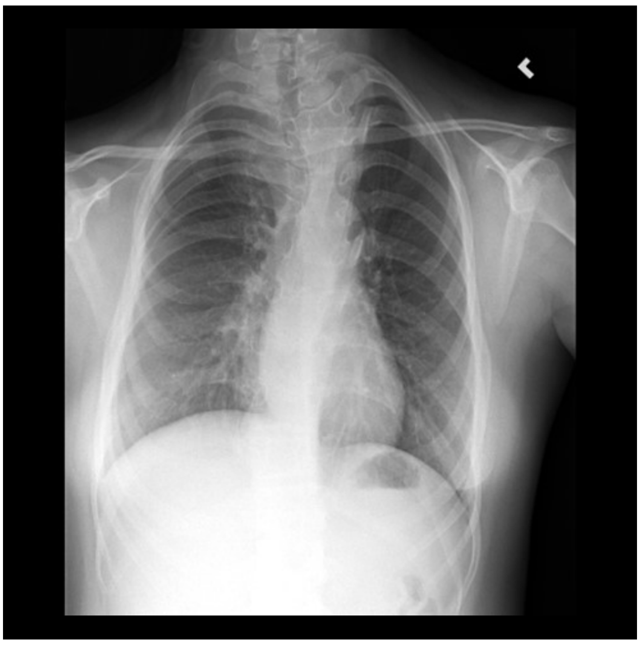

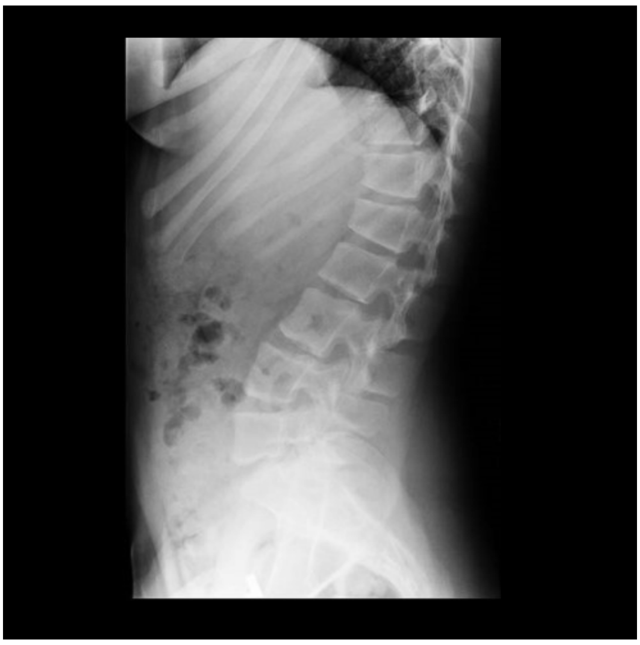

Case 5

Circle

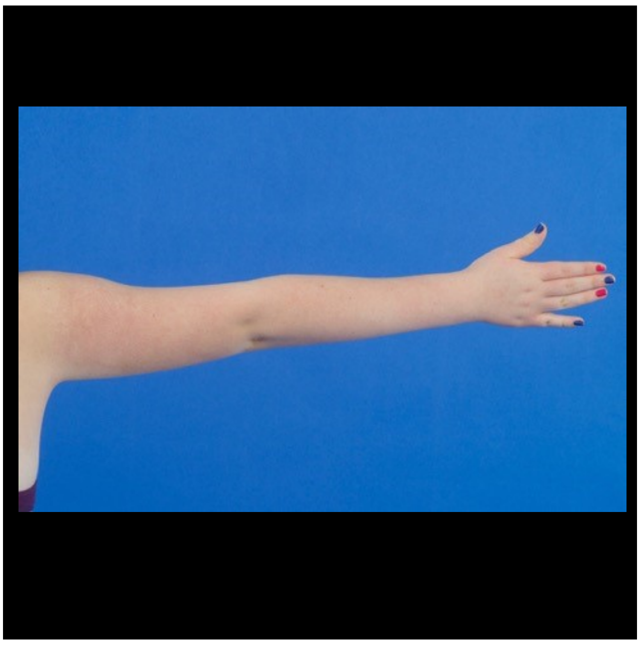

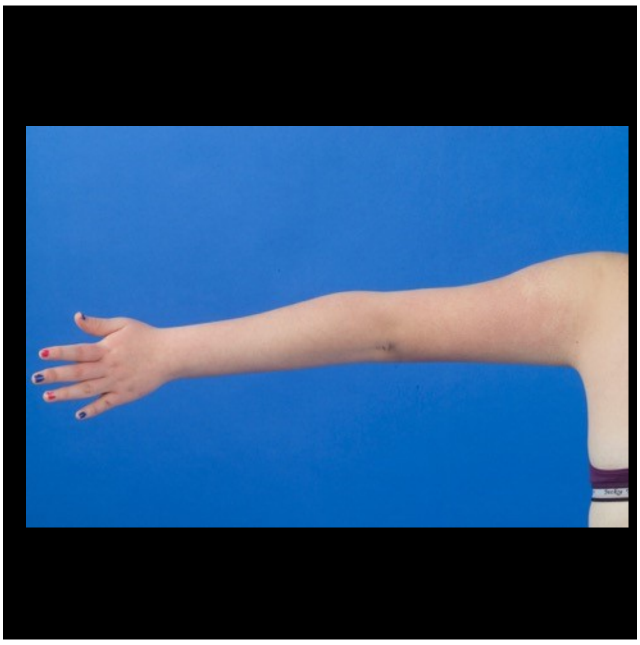

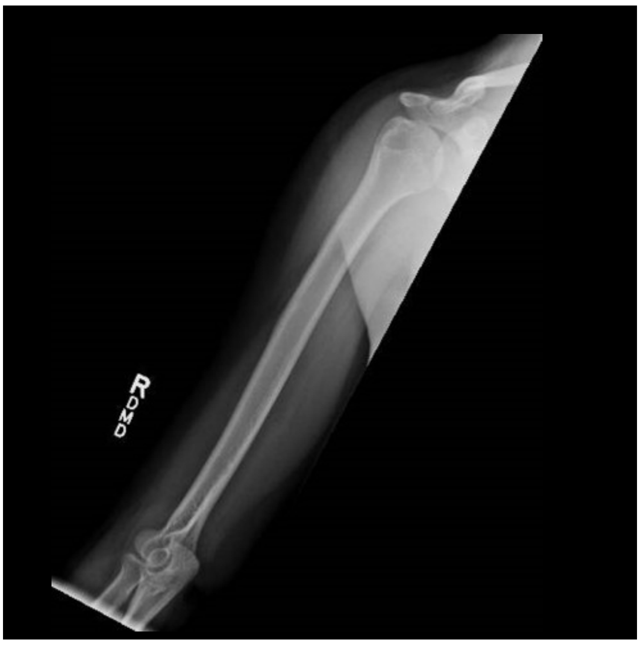

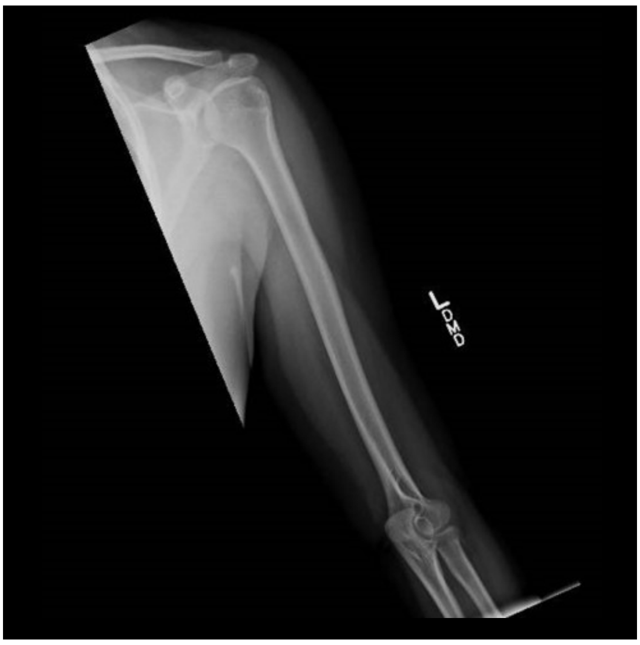

Square

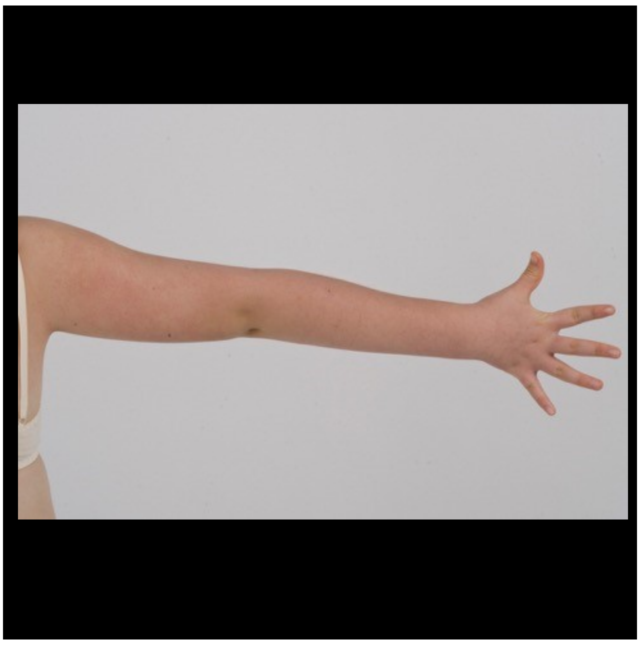

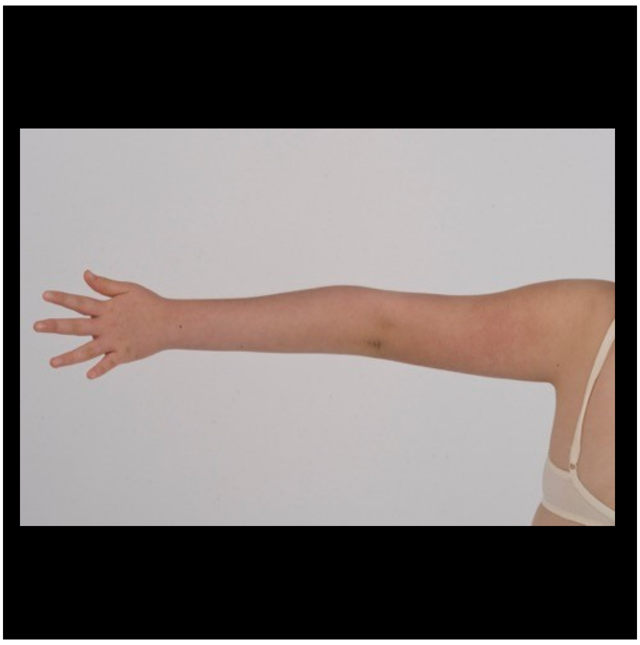

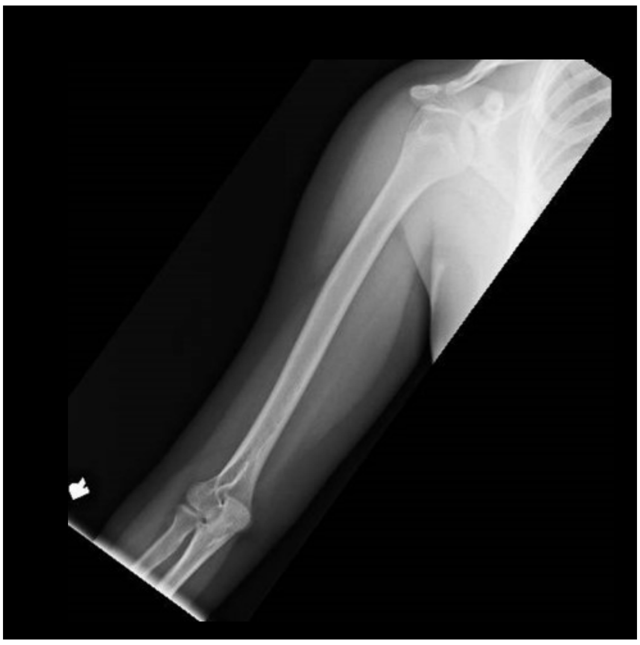

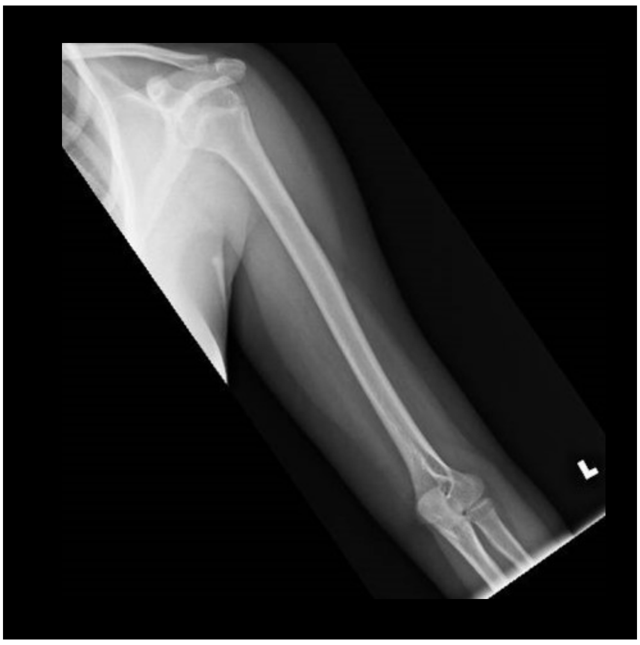

Case 5

Circle	Square

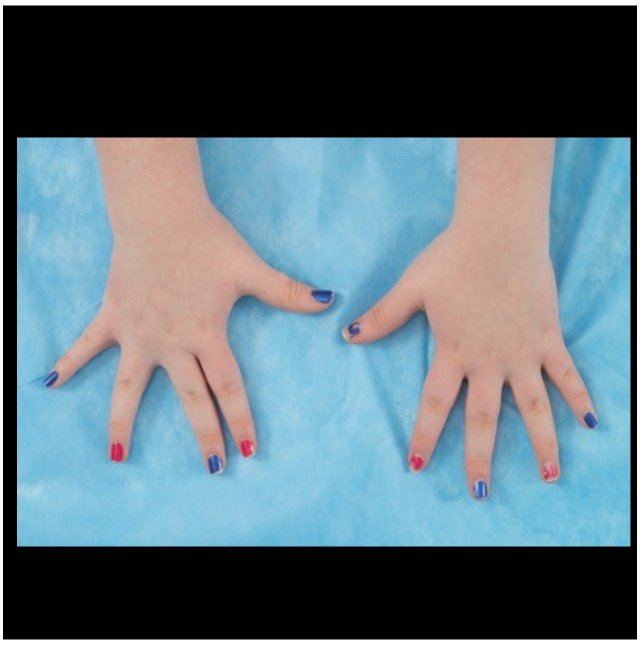

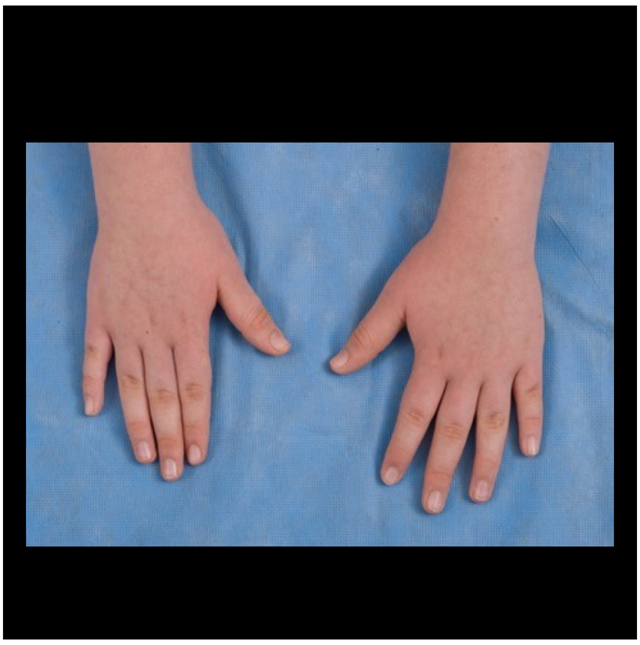

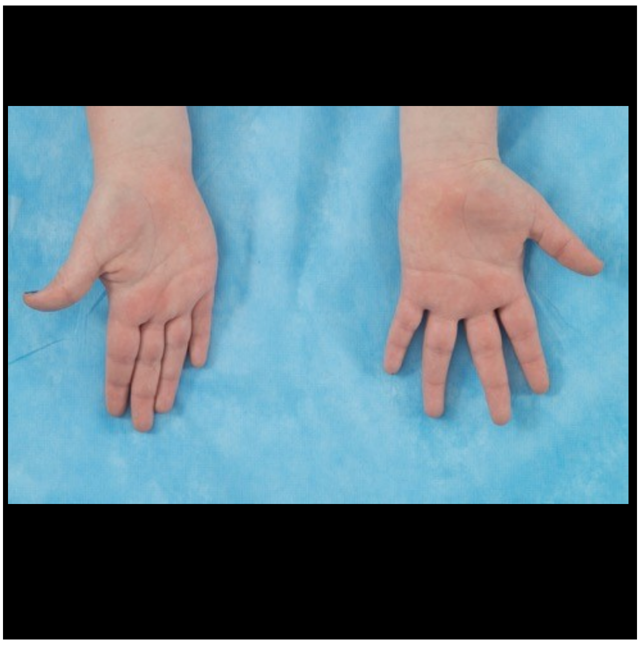

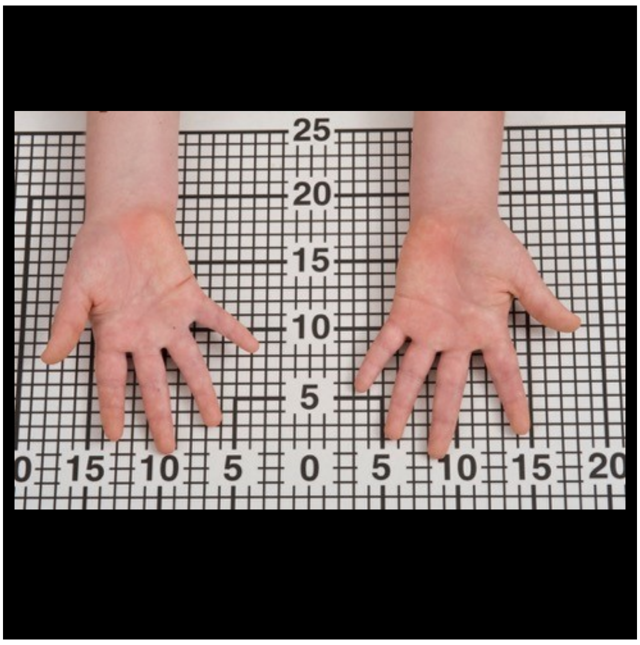

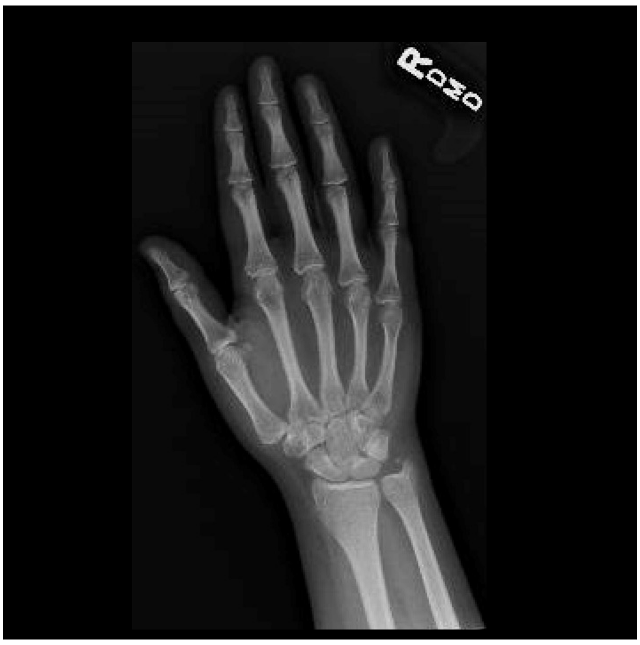

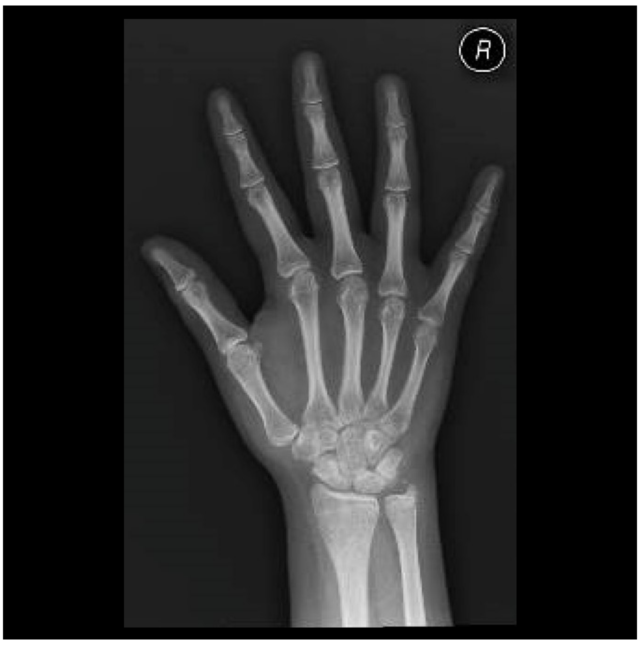

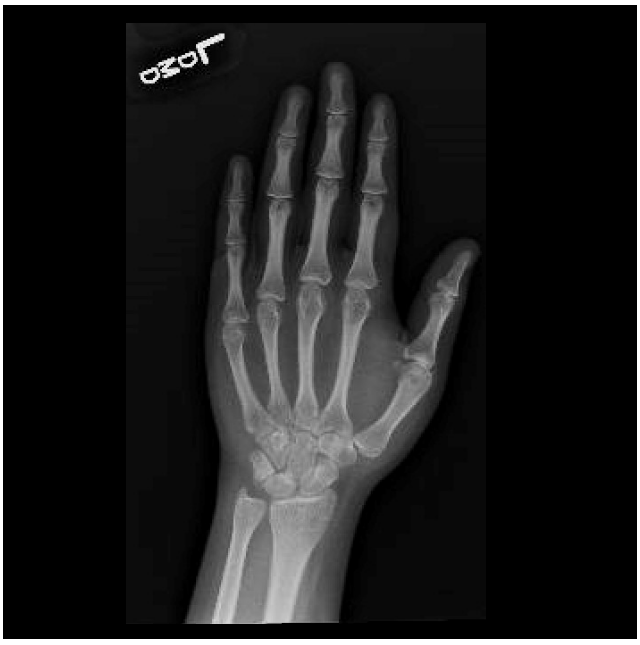

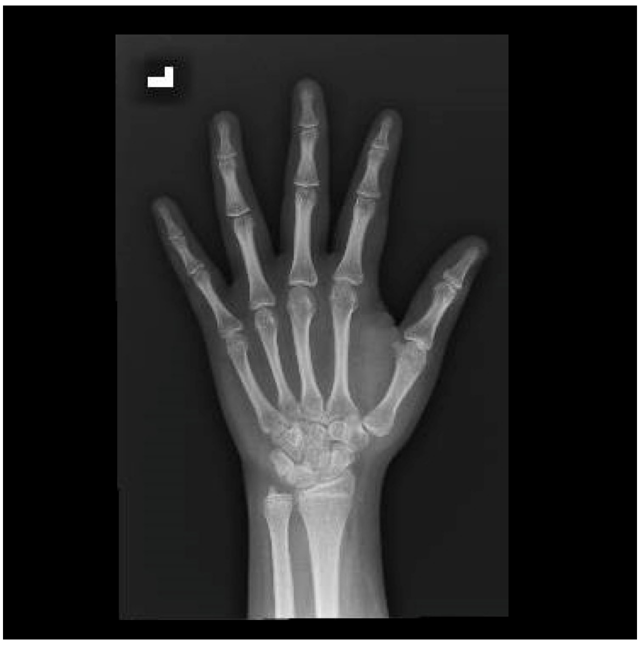

Case 5

Circle

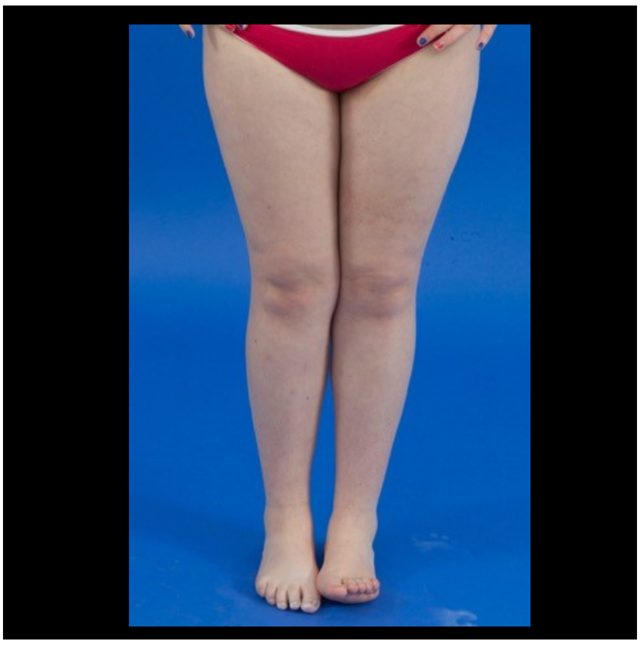

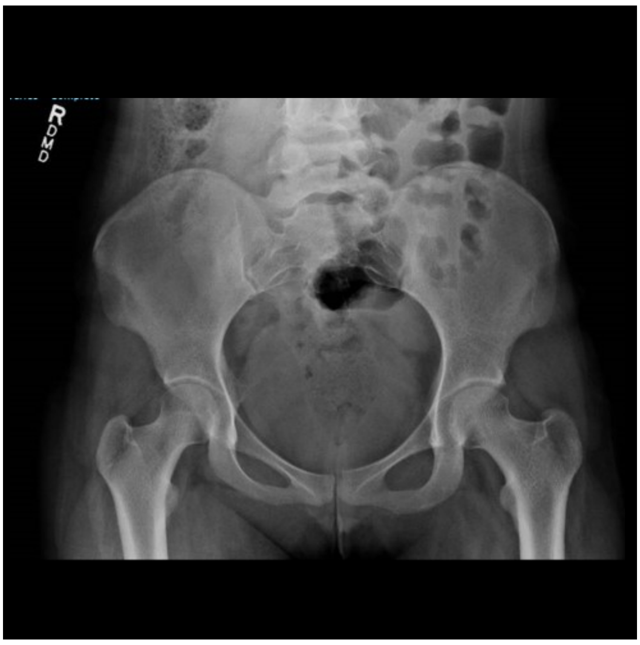

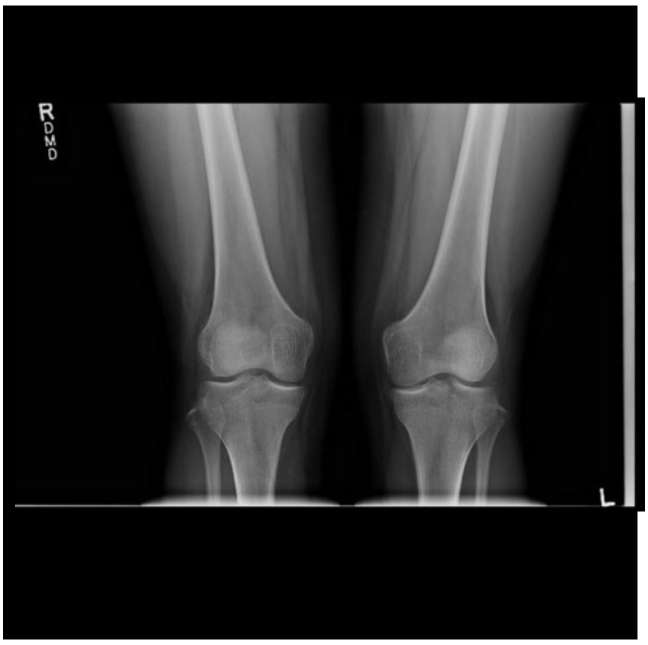

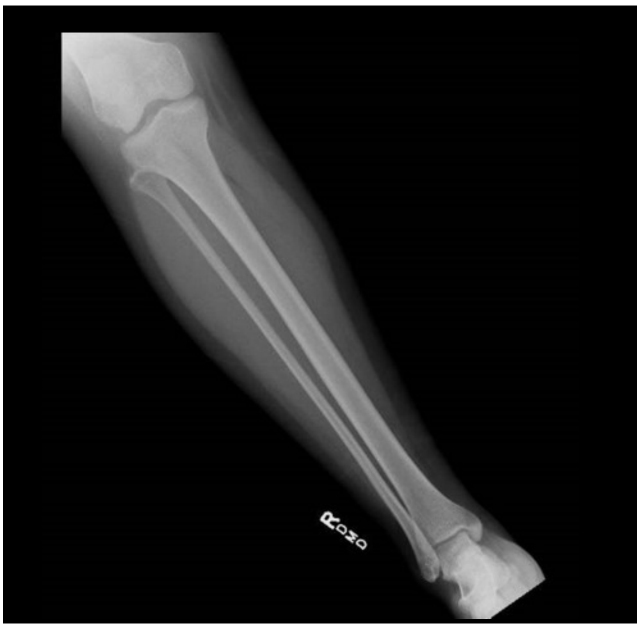

Square

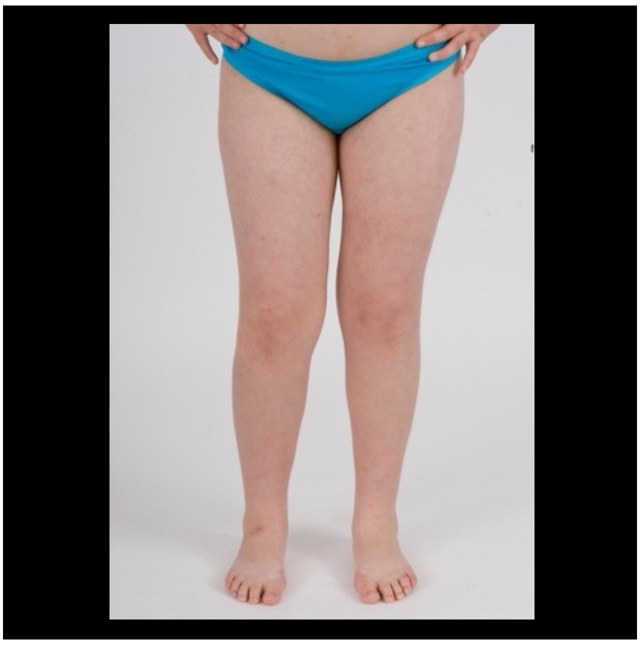

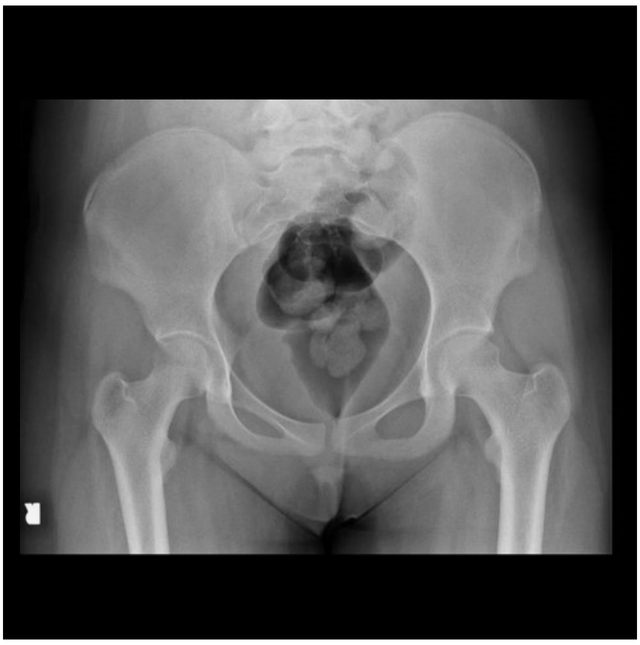

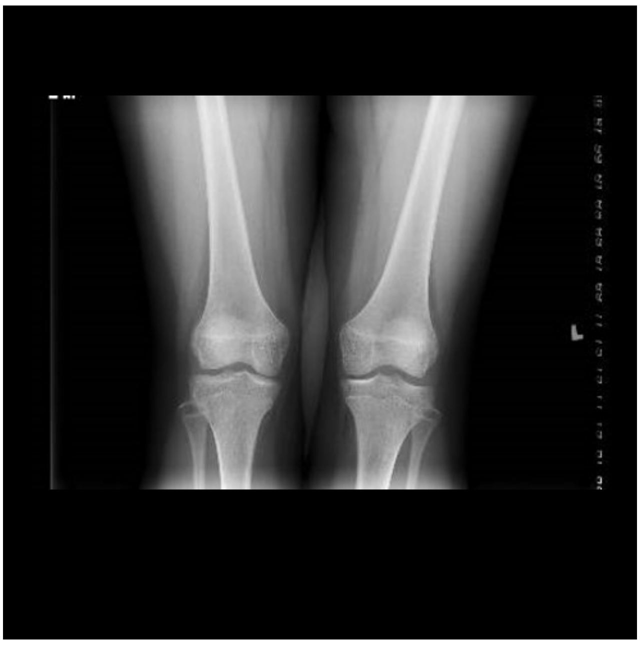

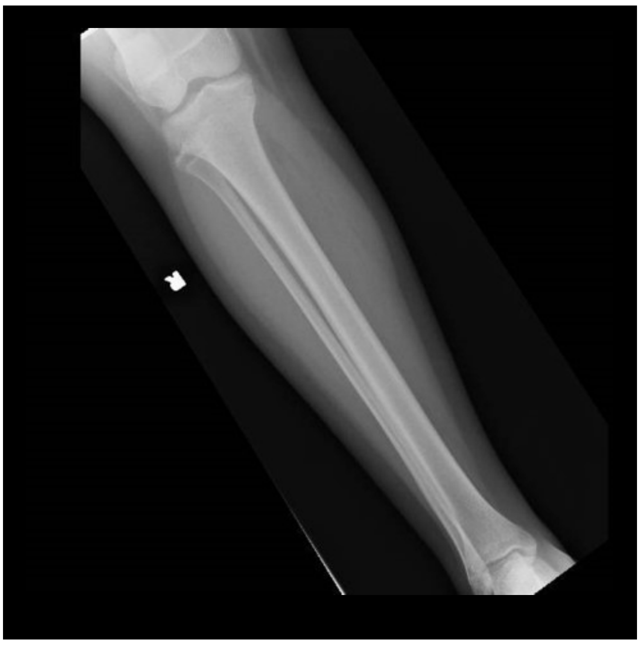

Case 5

Circle

Square

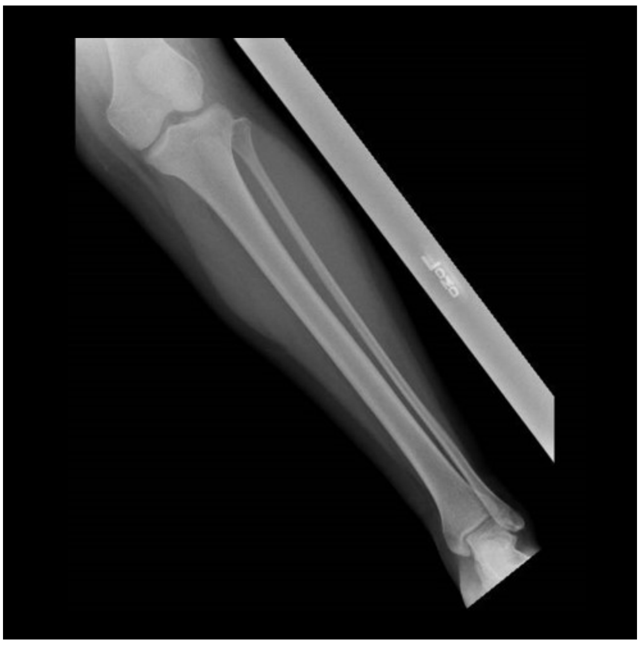

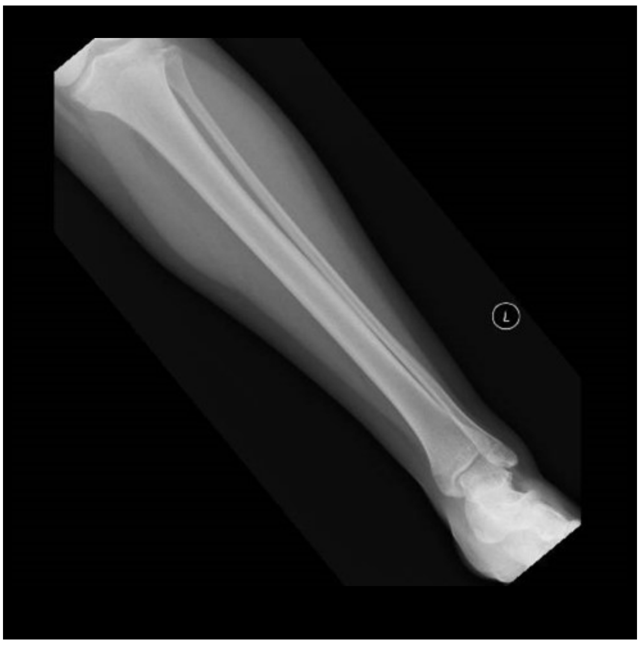

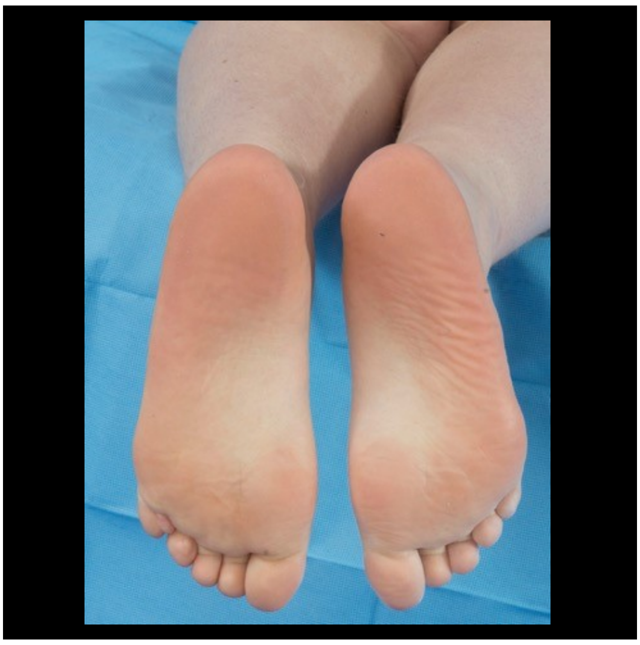

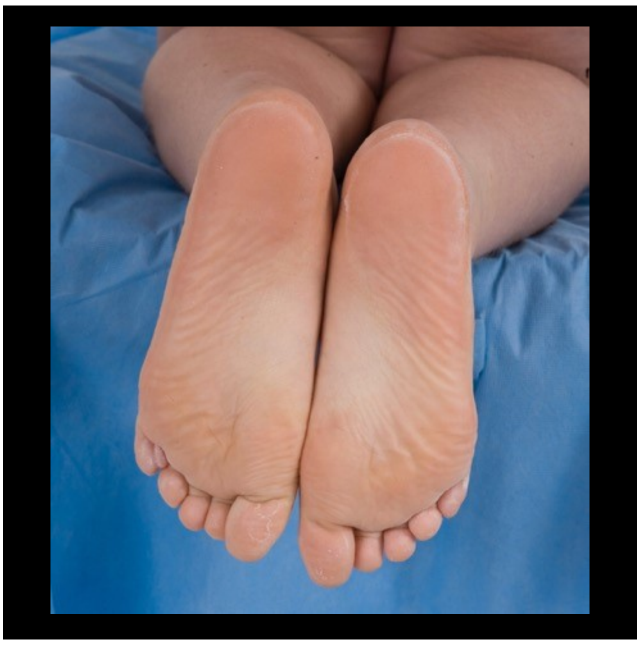

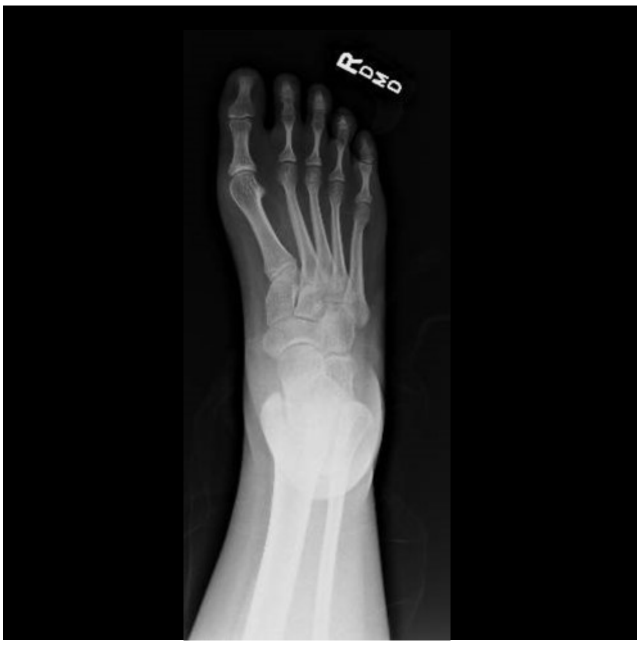

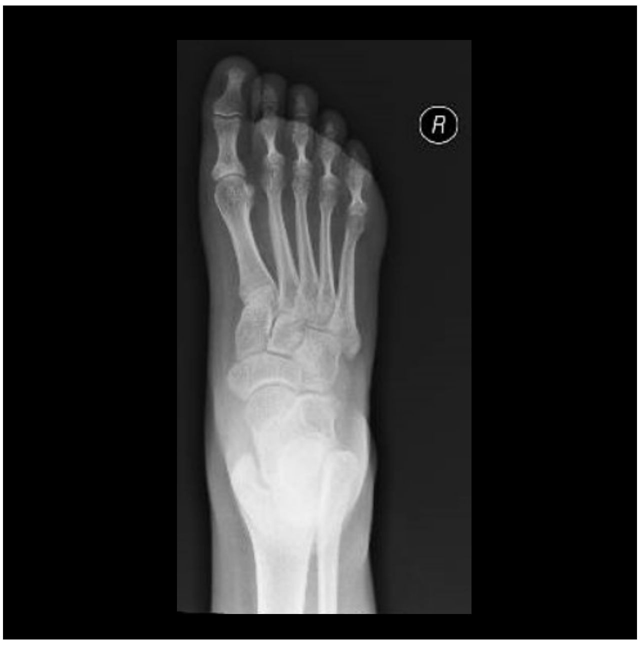

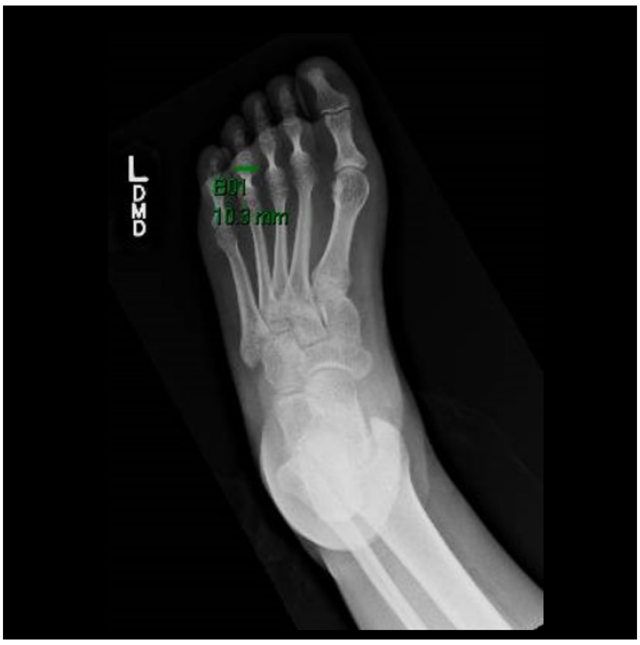

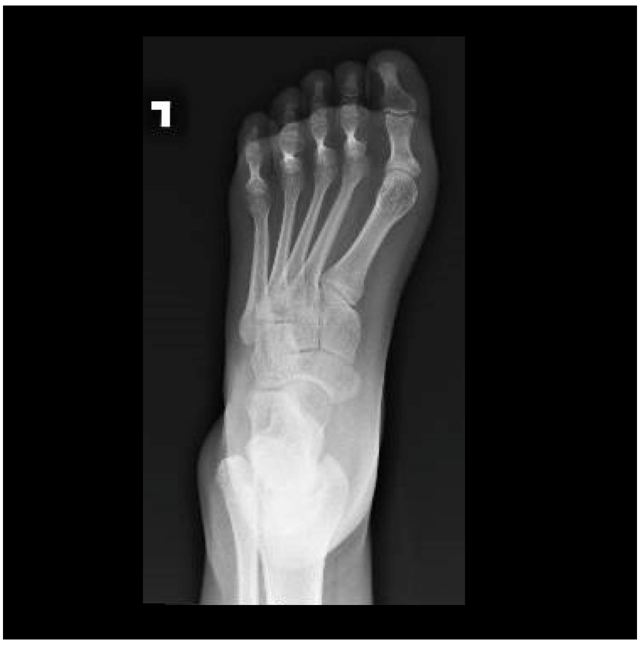

Case 6

Case 6

Circle

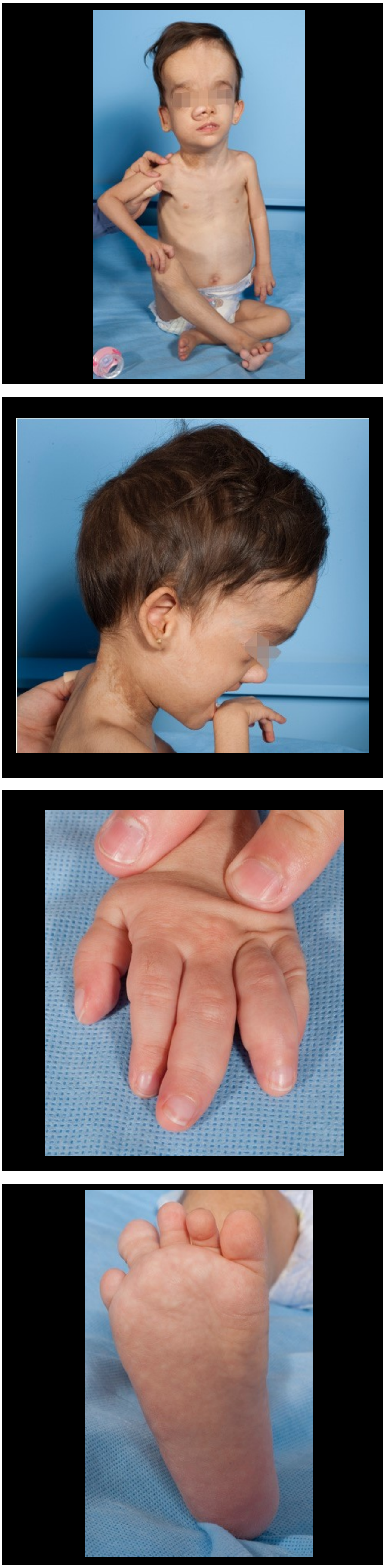

Square

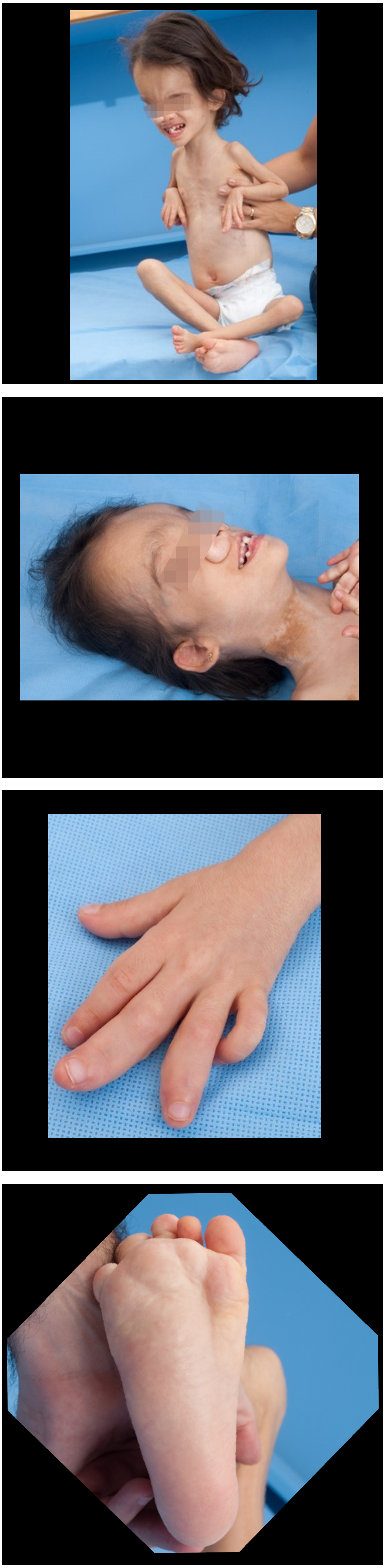

Case 6

Circle

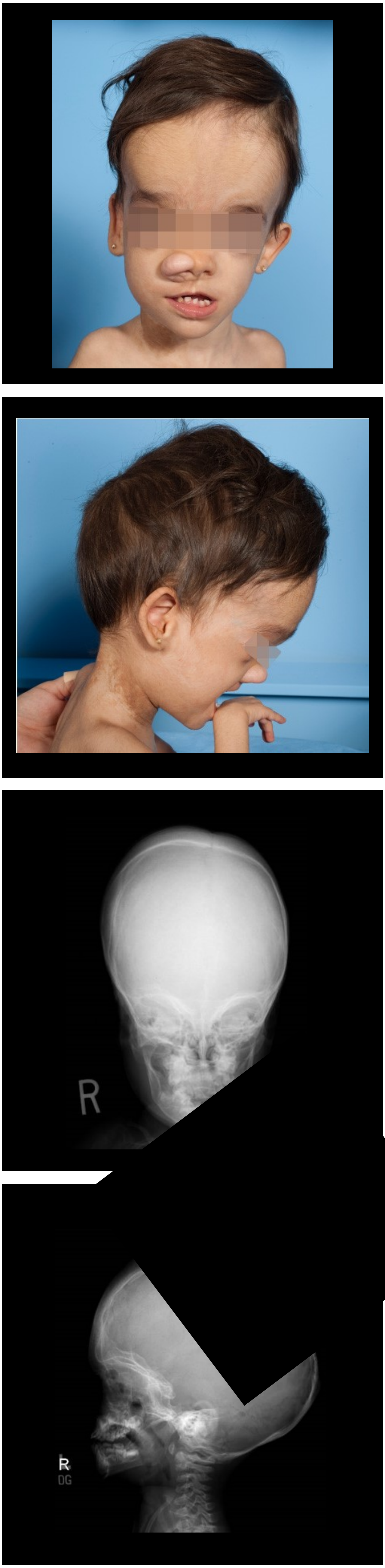

Square

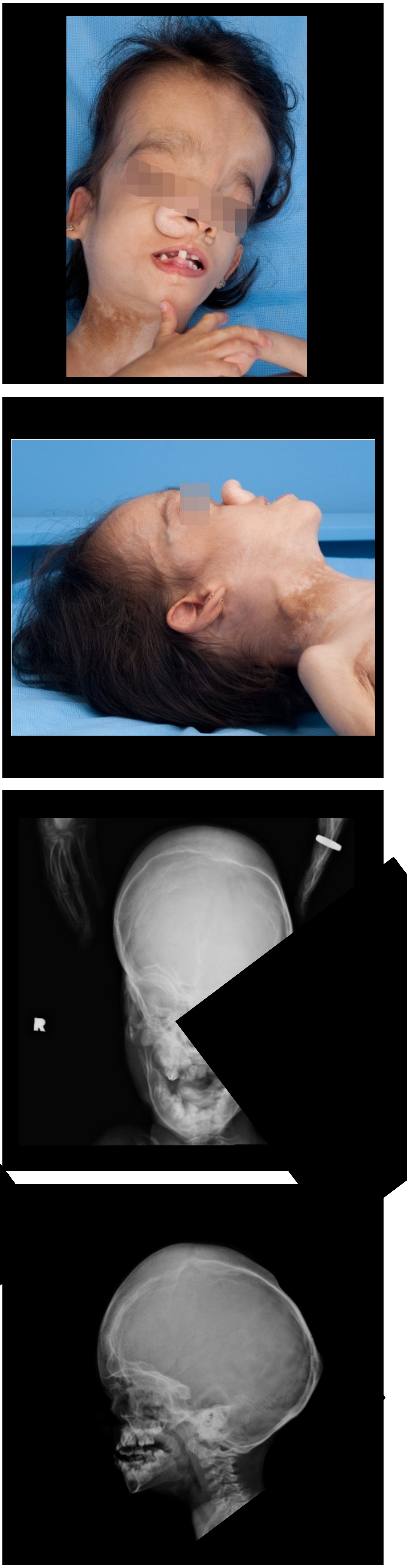

Case 6

Circle

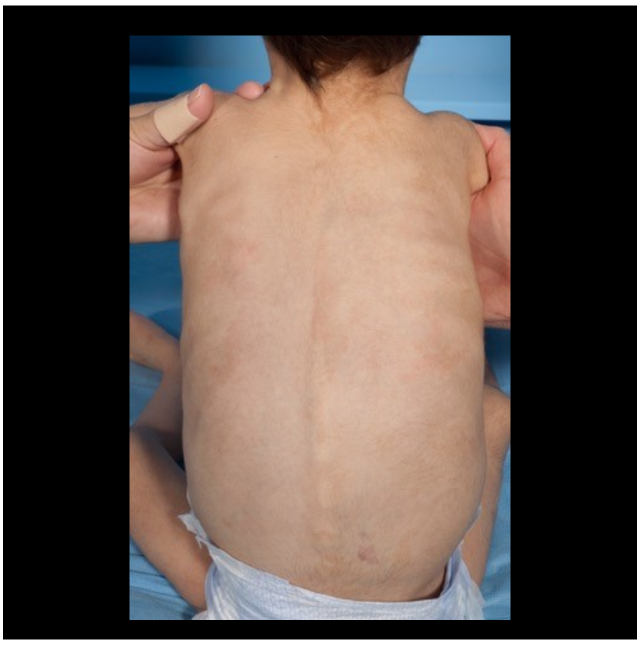

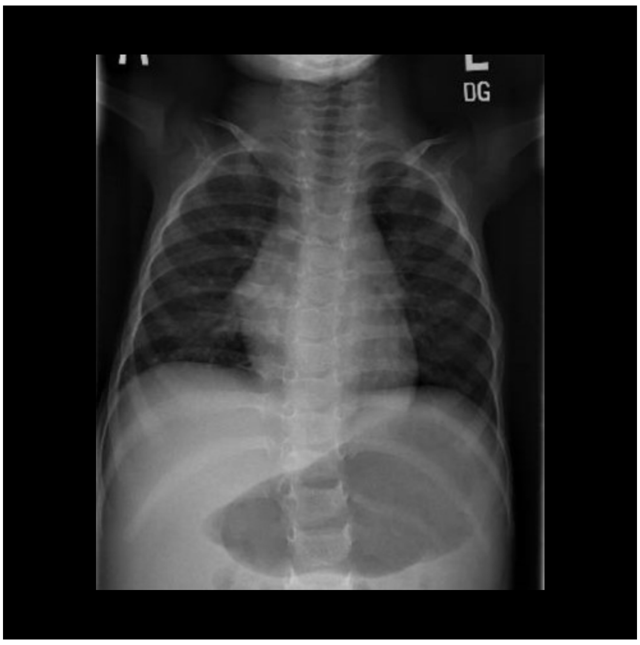

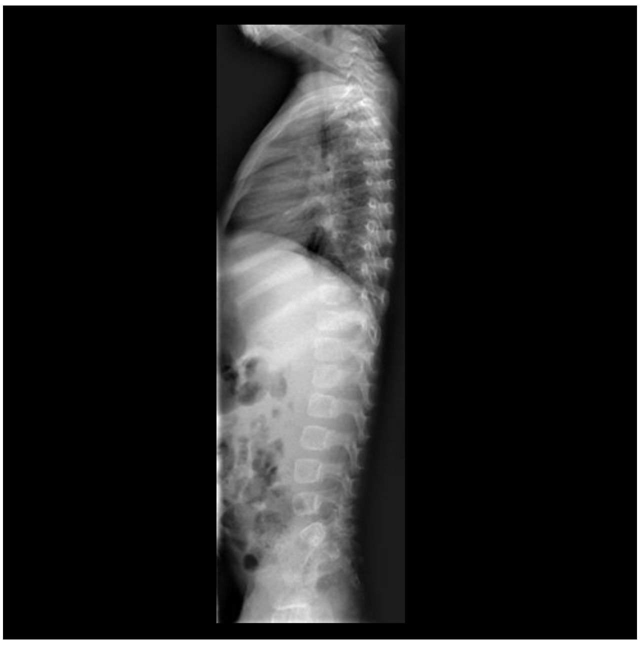

Square

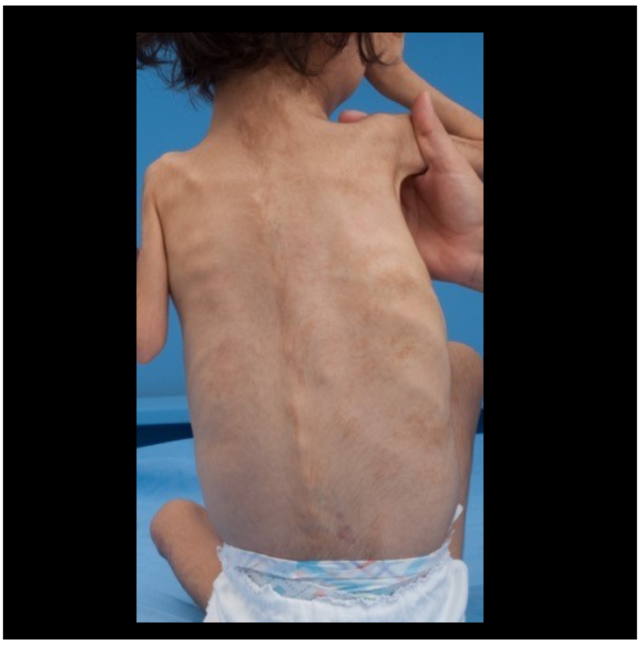

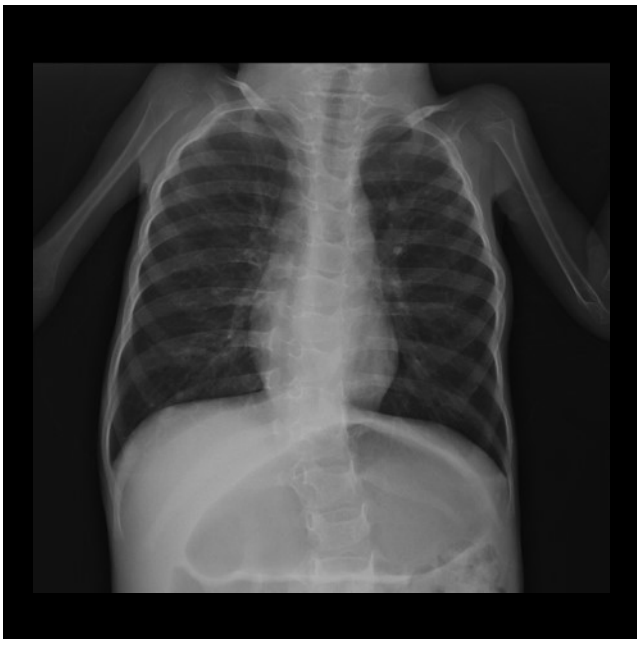

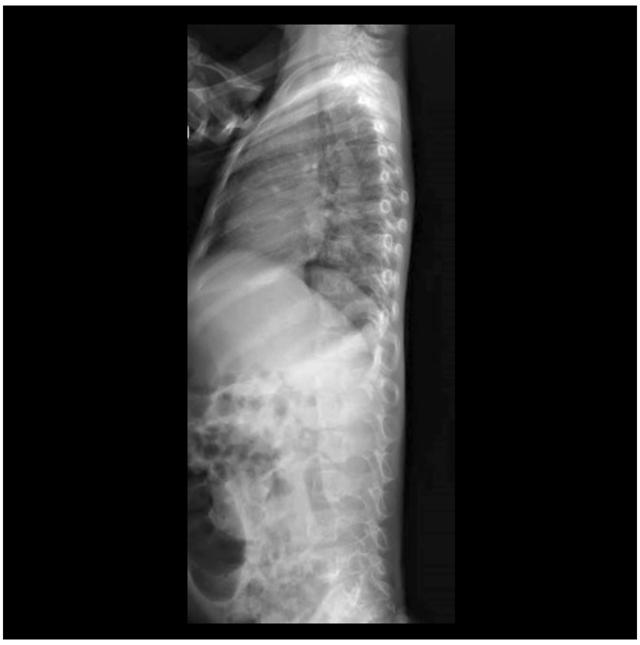

Case 6

Circle

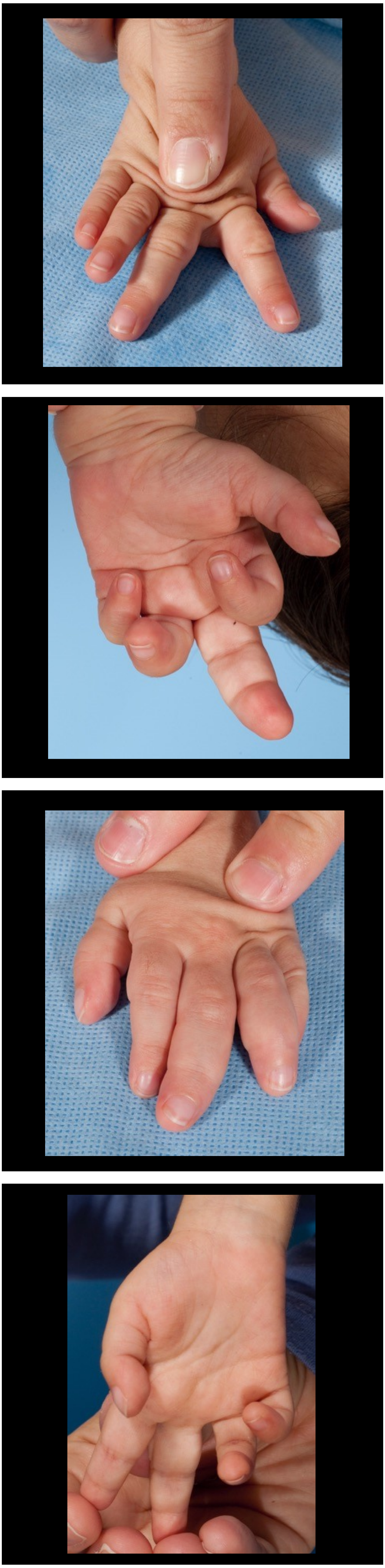

Square

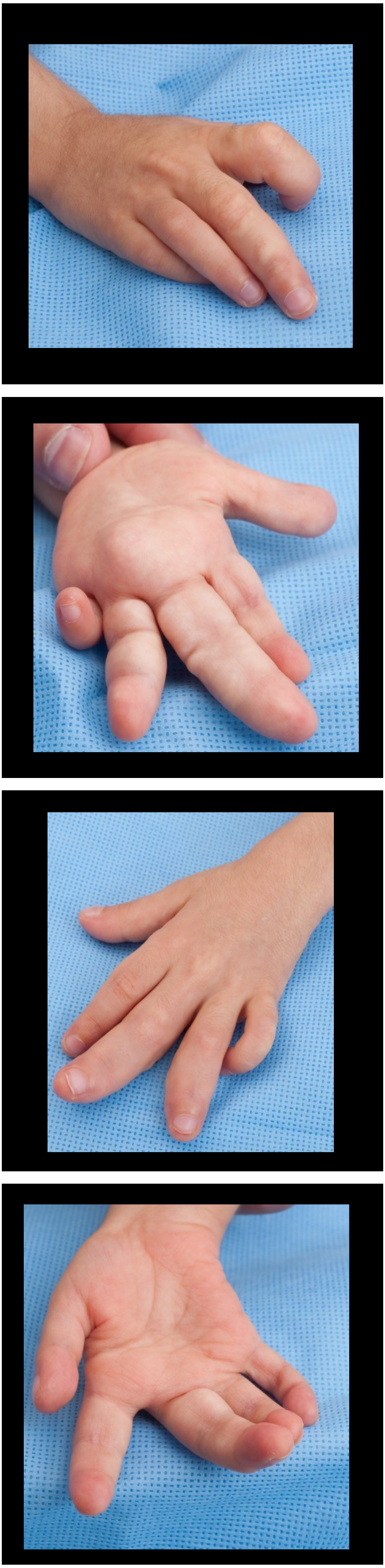

Case 6

Circle

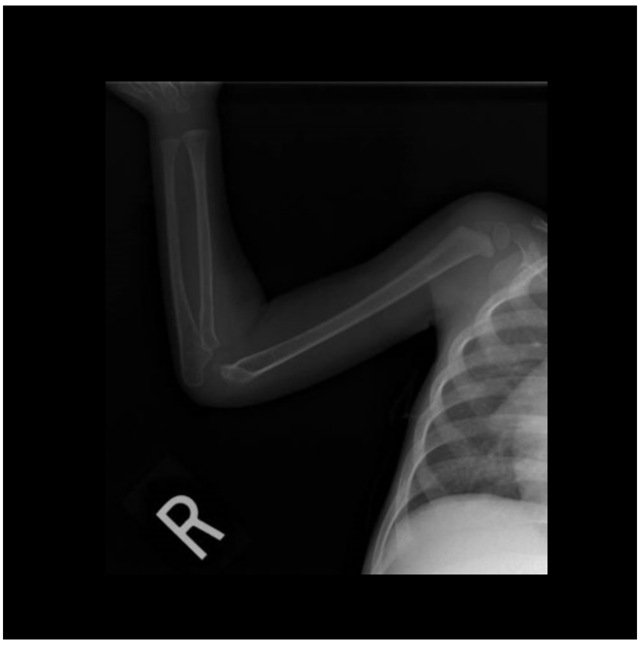

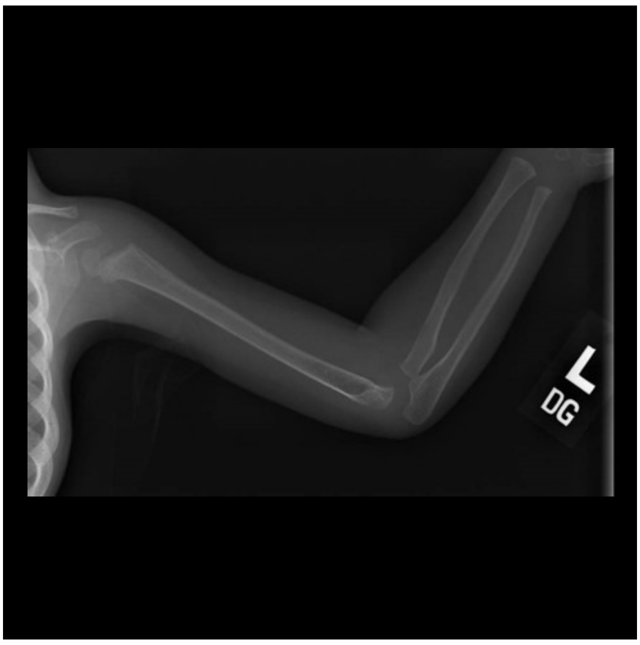

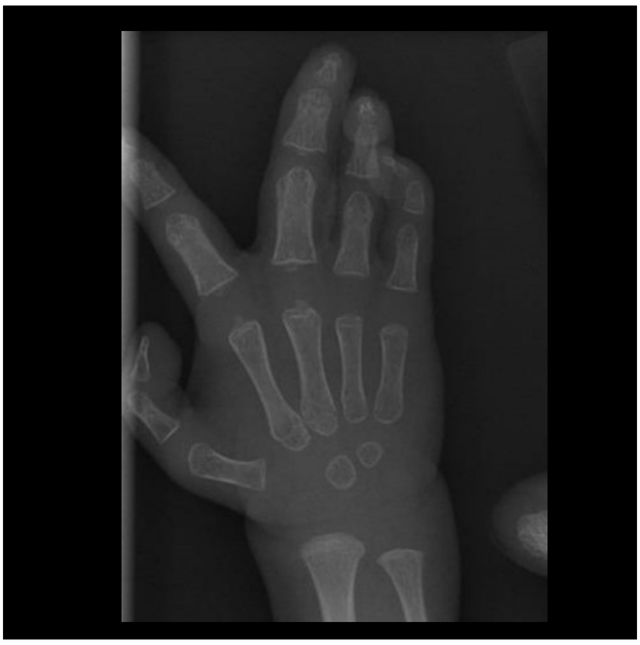

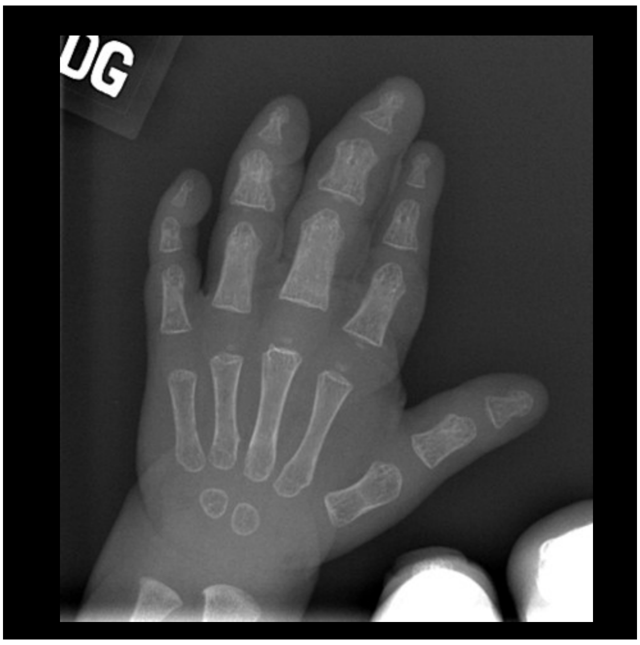

Square

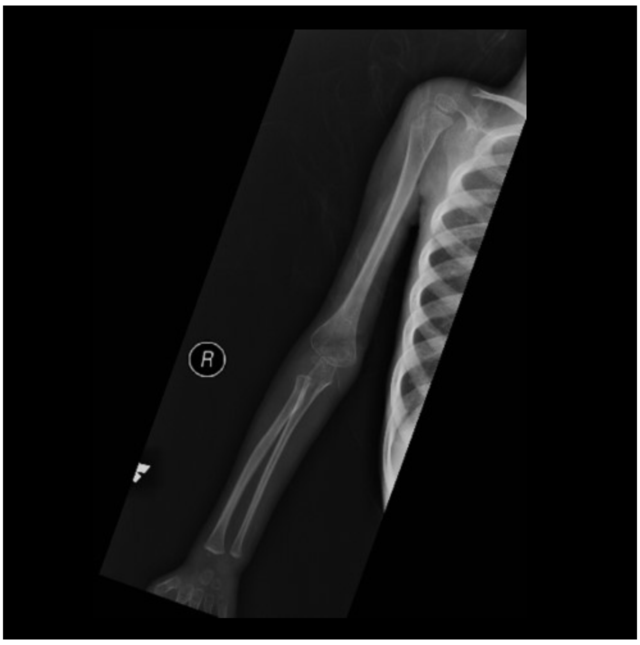

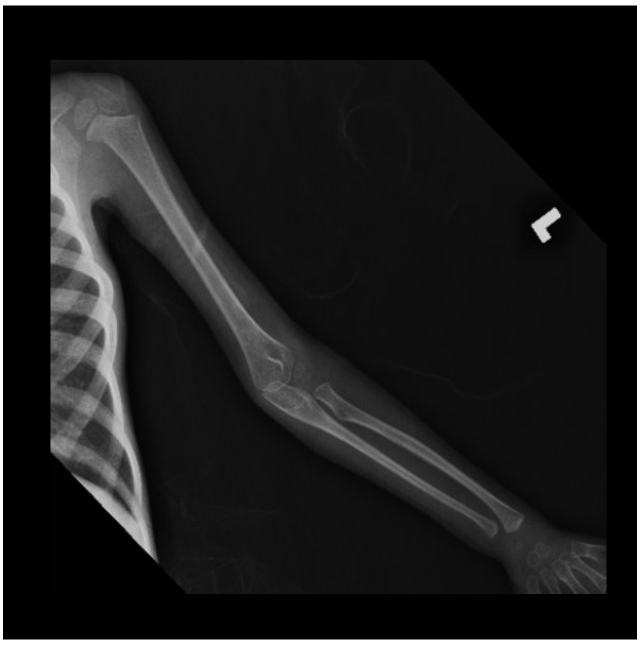

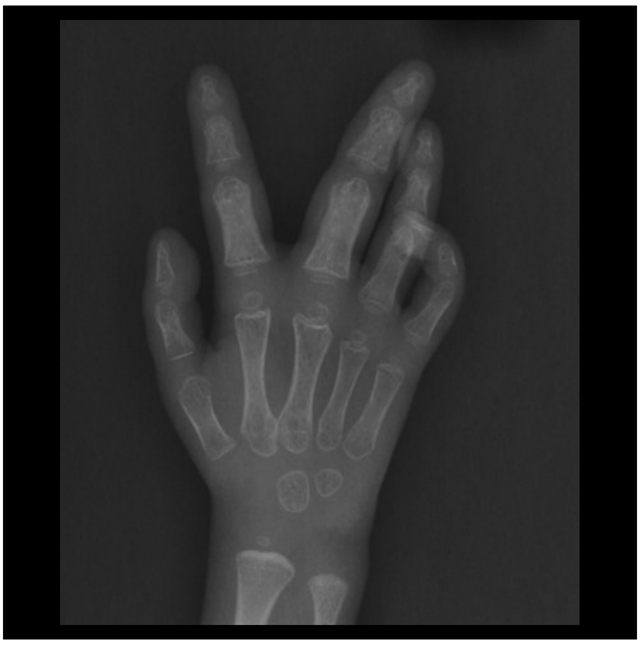

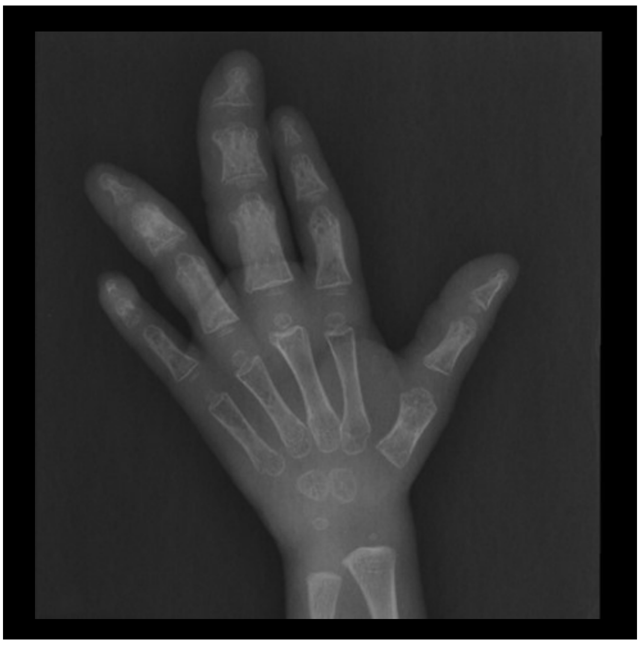

Case 6

Circle

Square

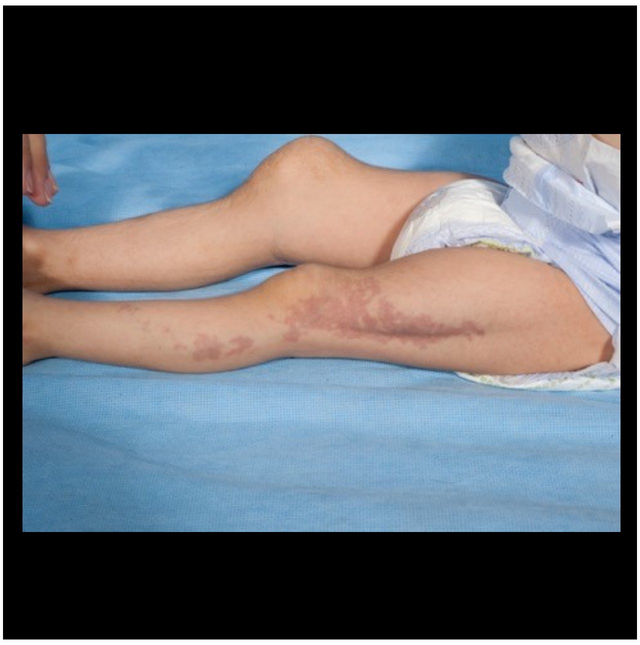

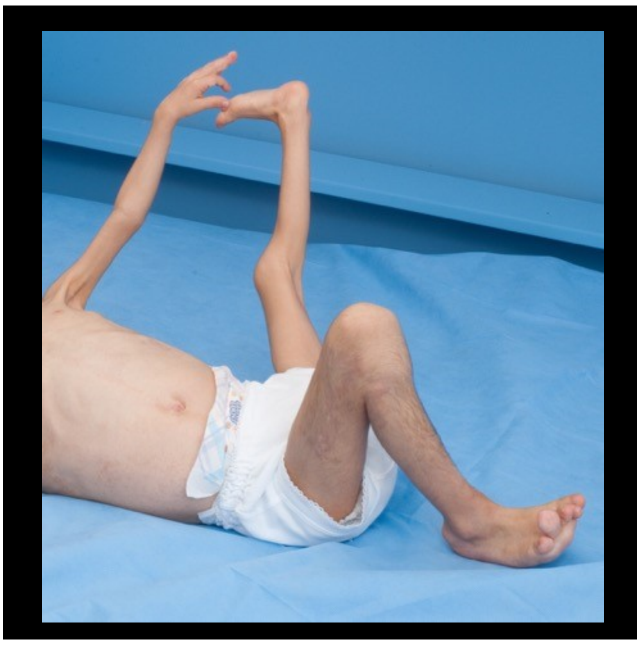

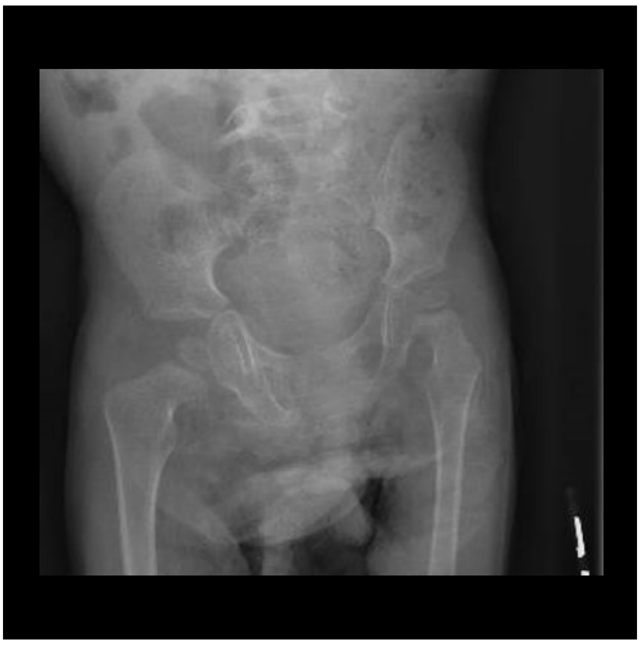

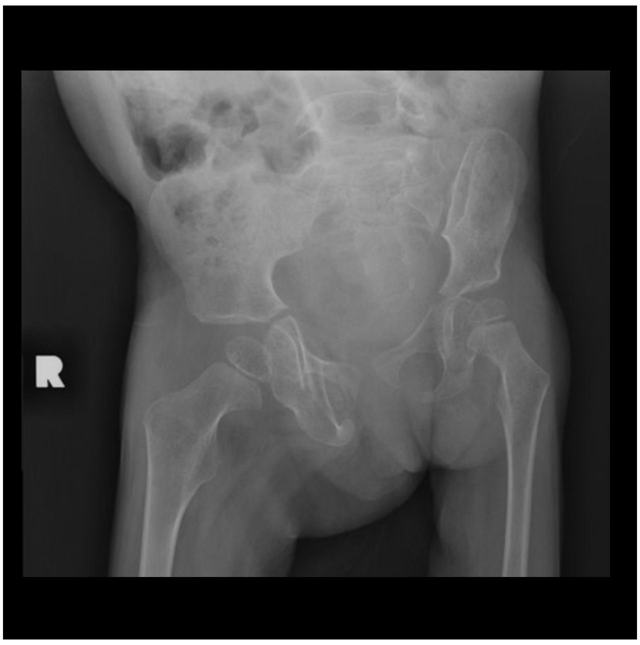

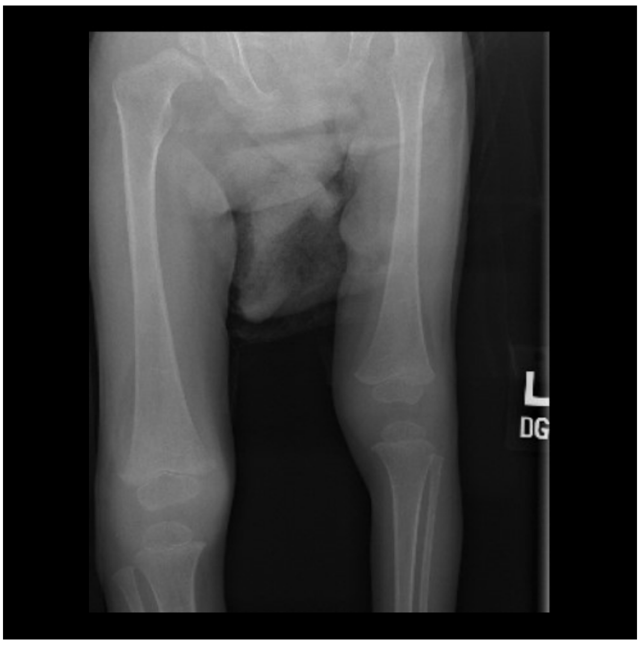

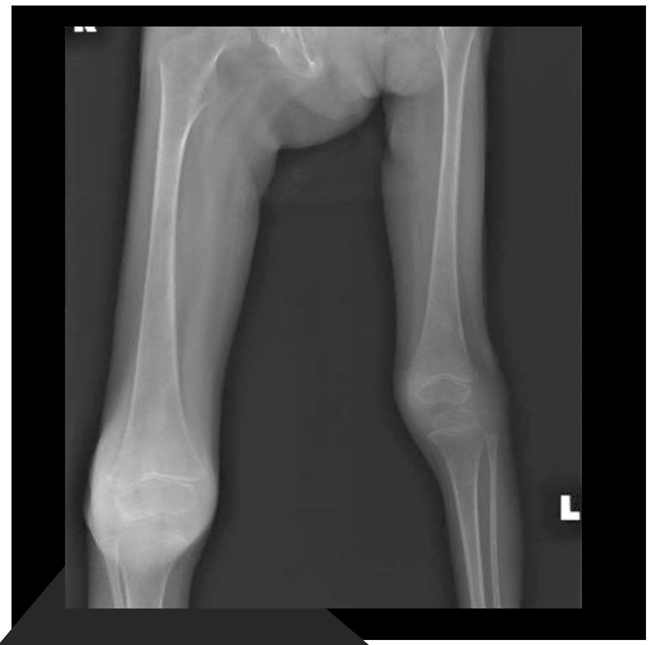

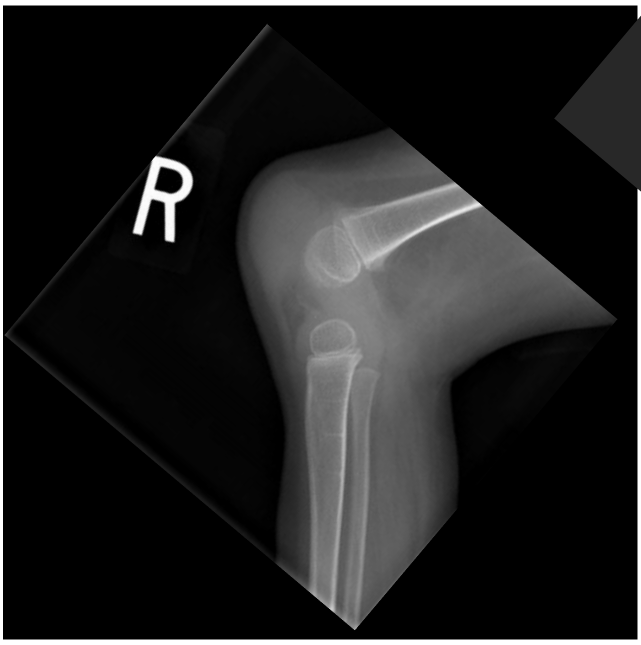

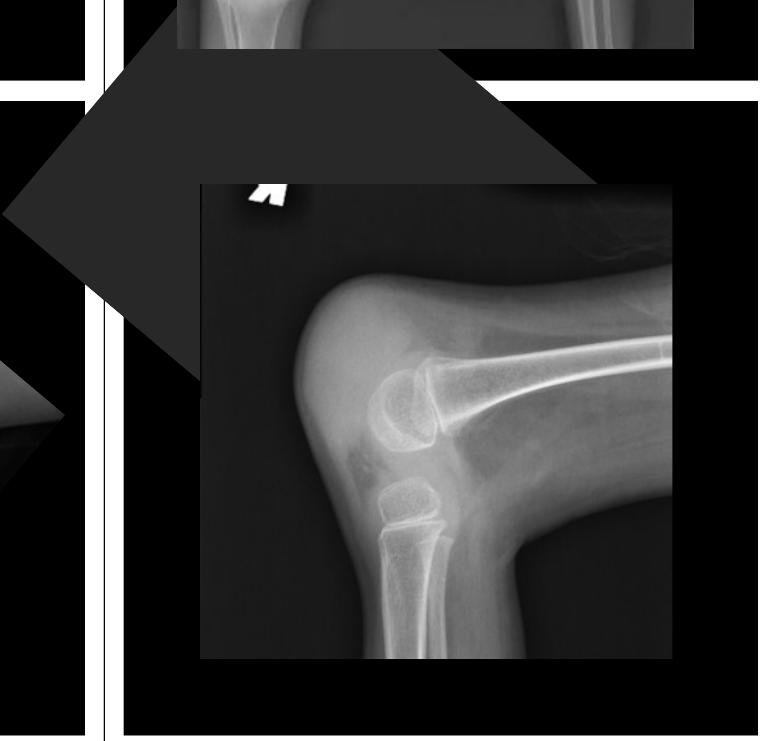

Case 6

Circle

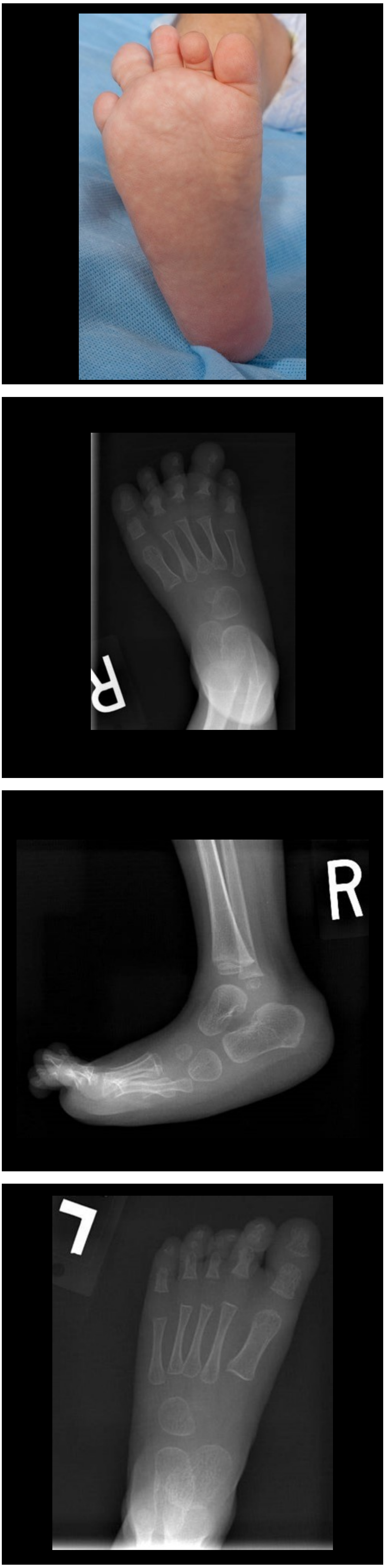

Square

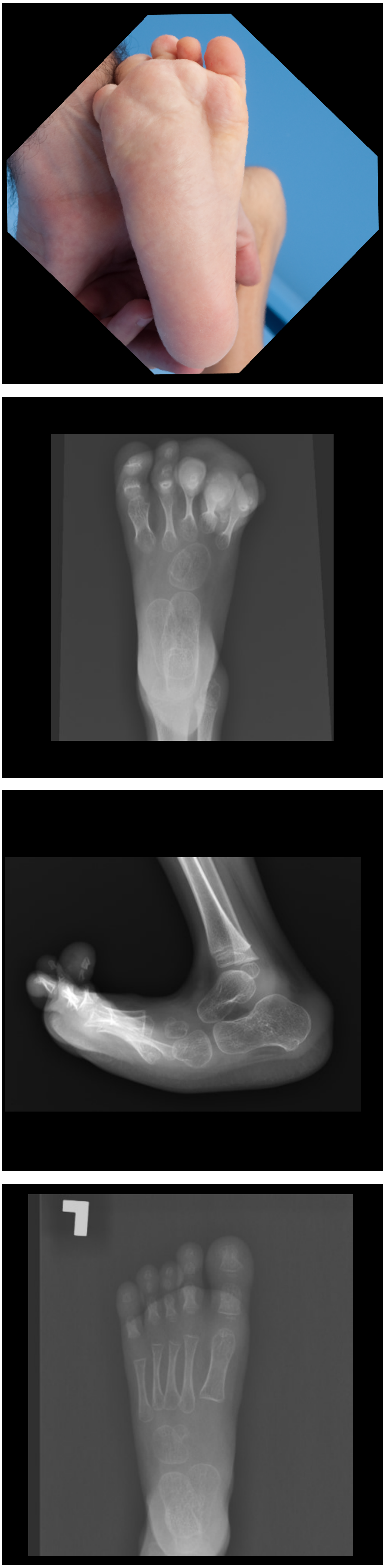

Case 7

Case 7

Circle

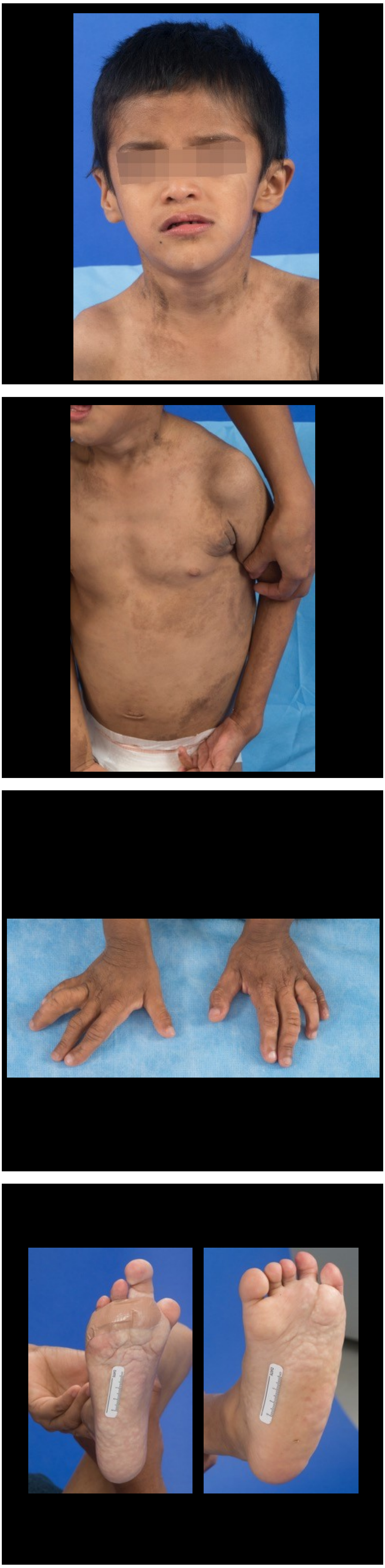

Square

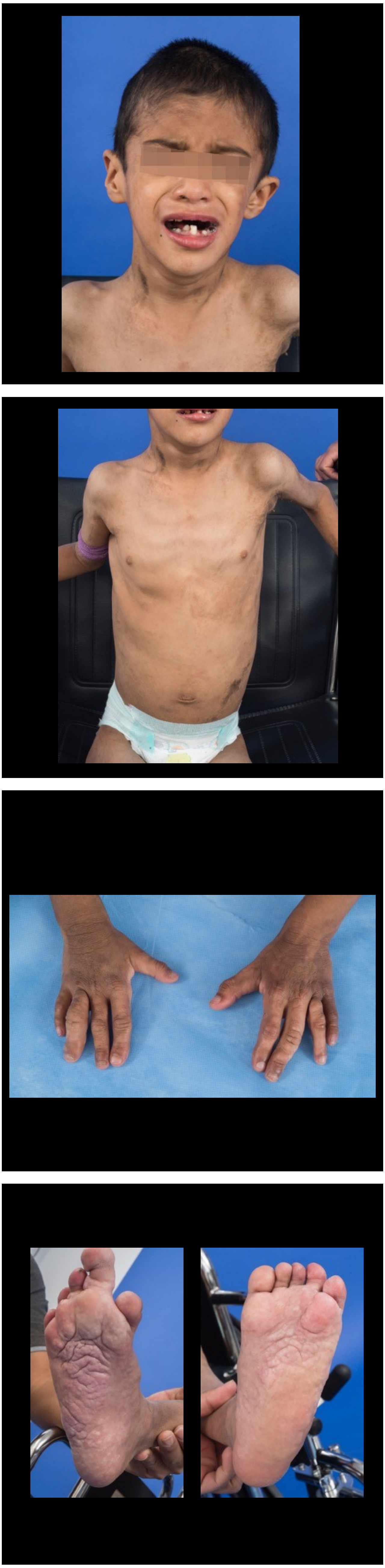

Case 7

Circle

Square

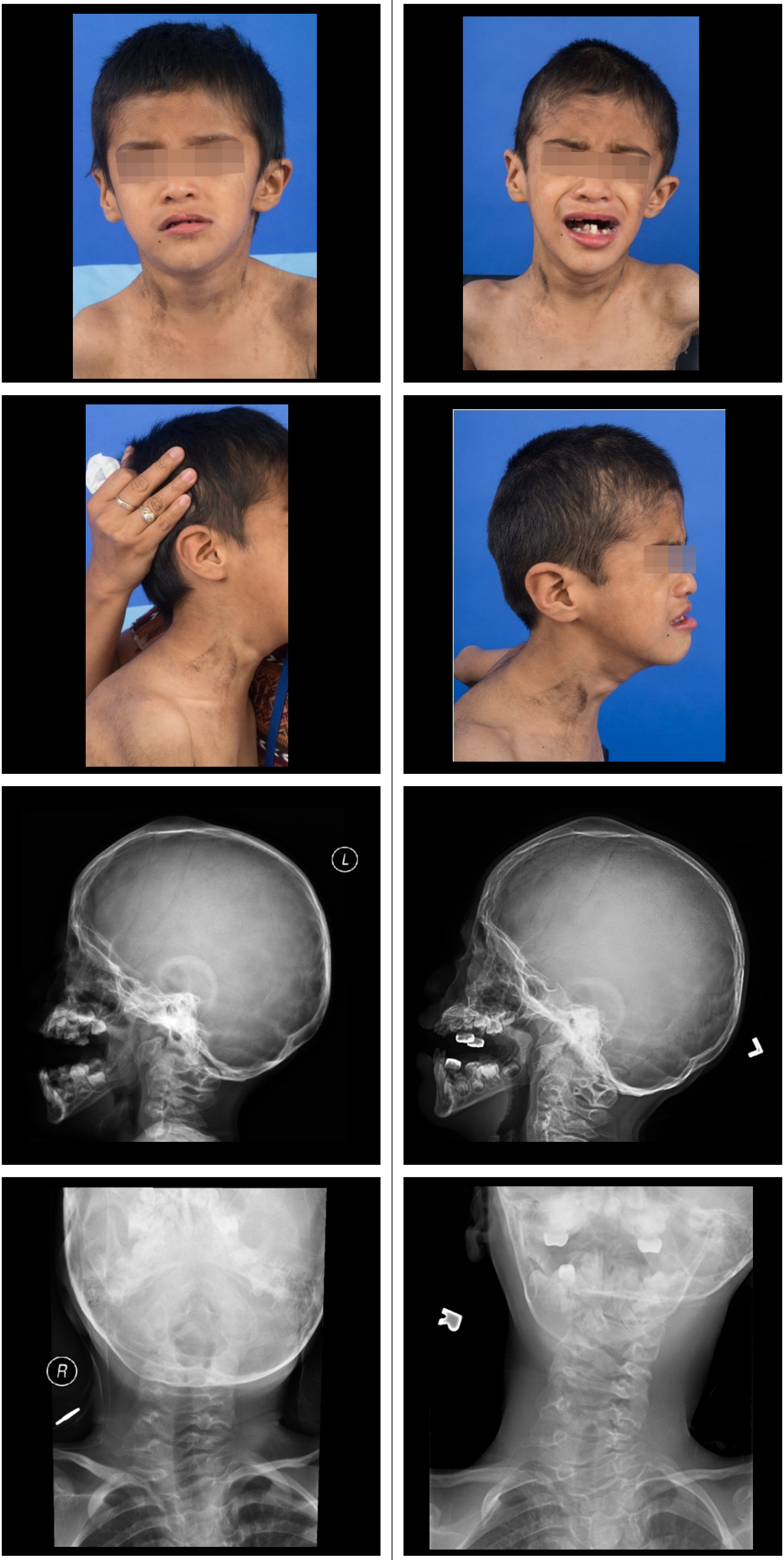

Case 7

Circle

Square

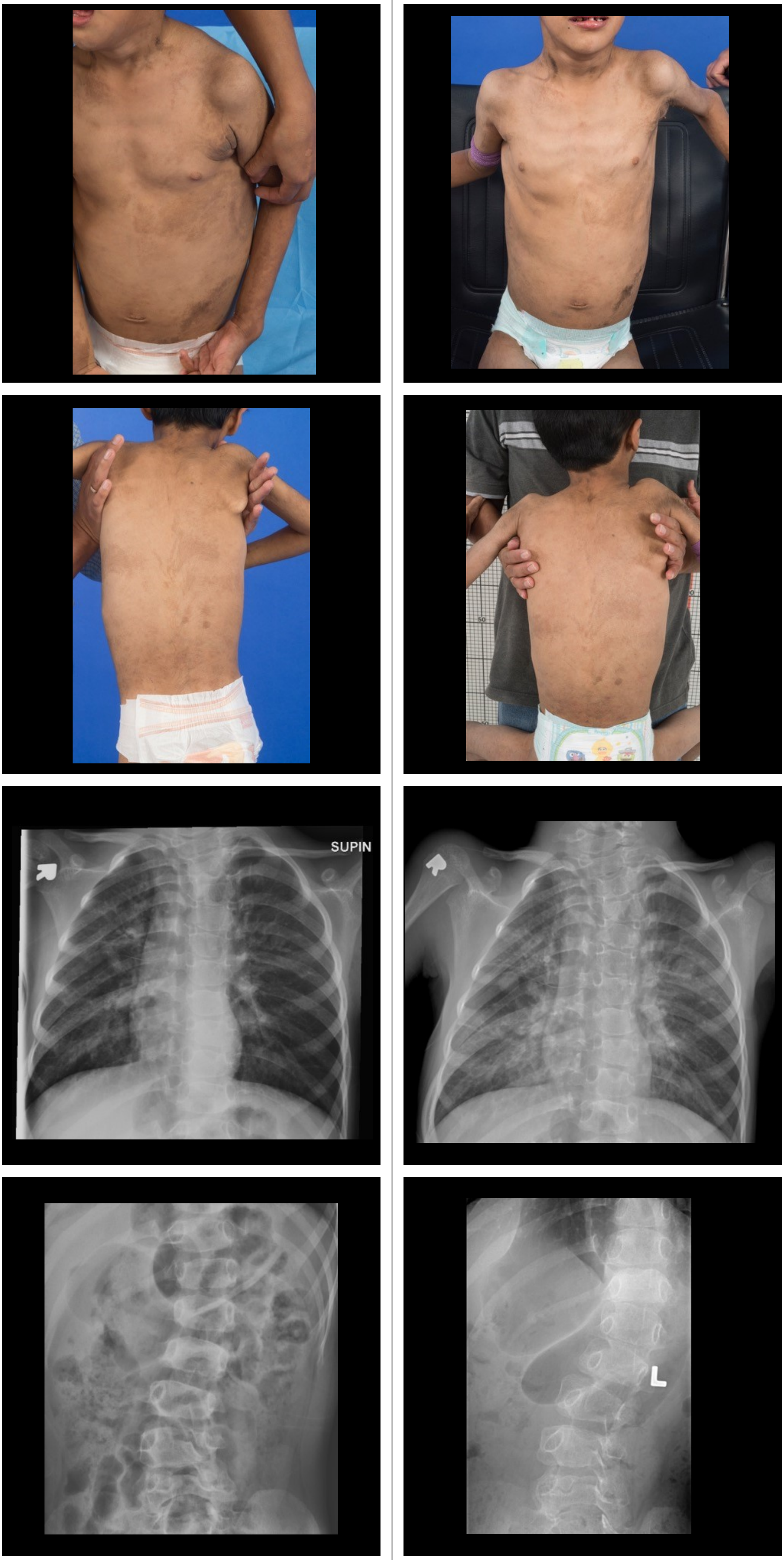

Case 7

Circle

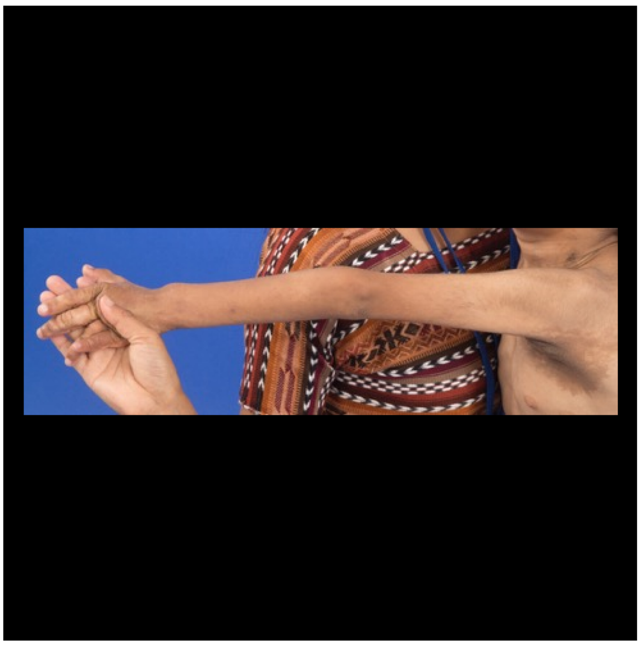

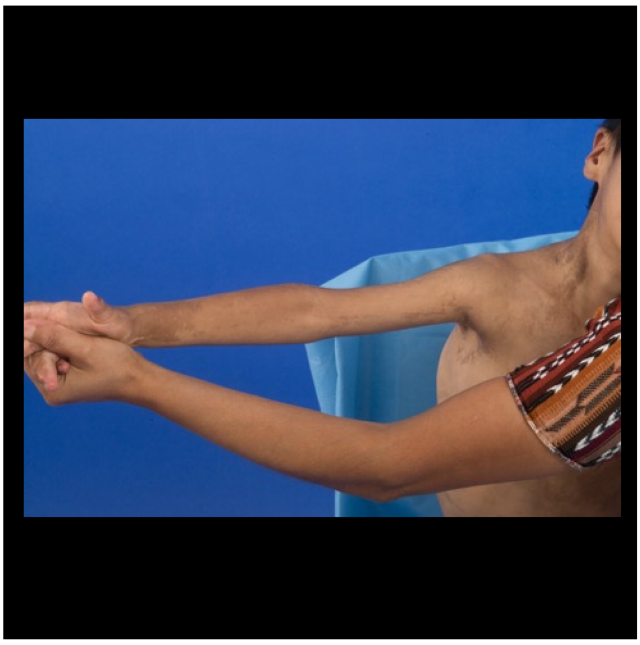

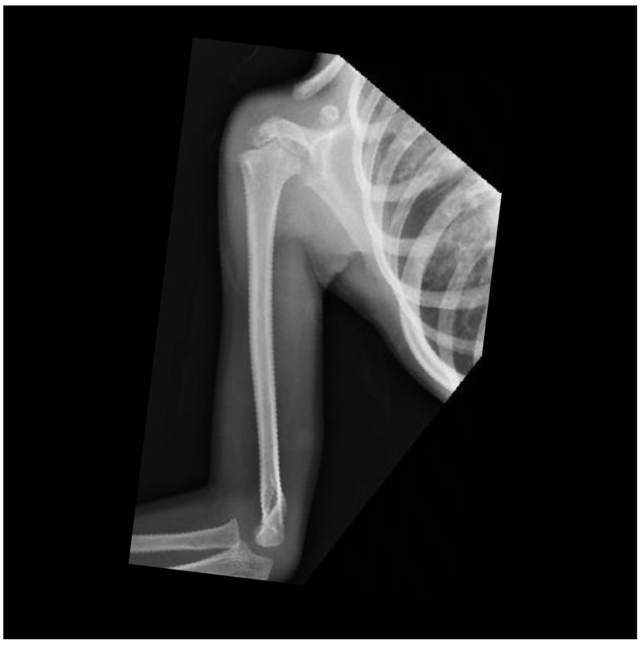

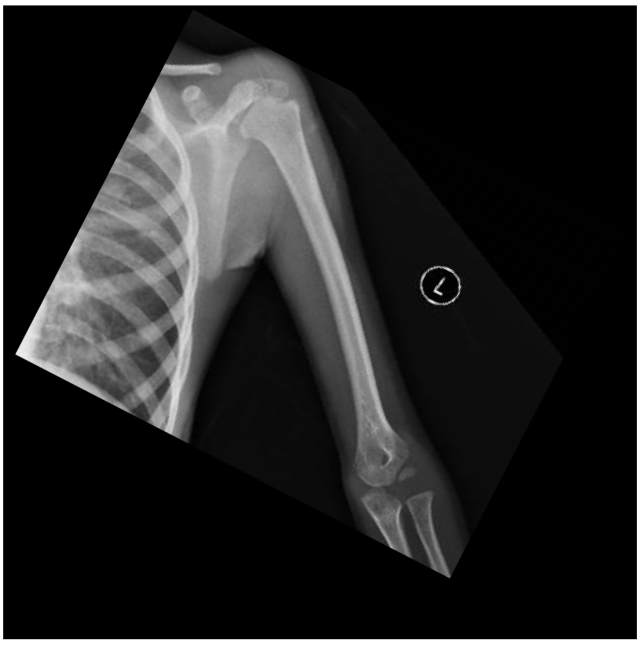

Square

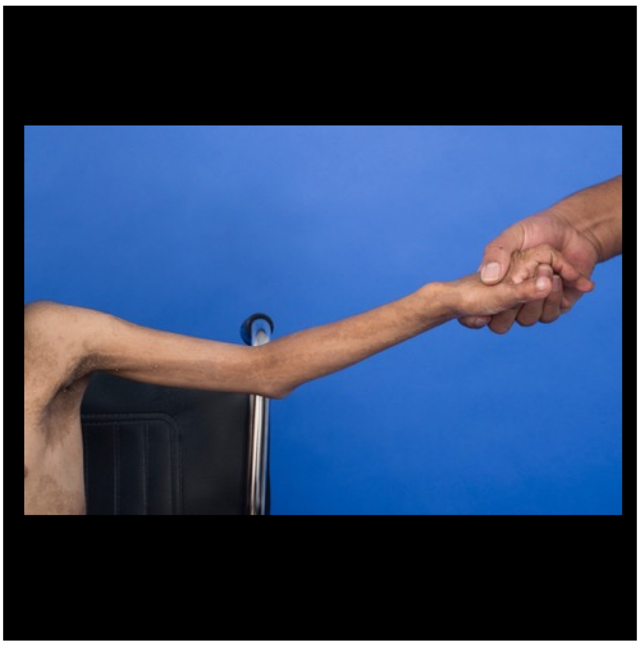

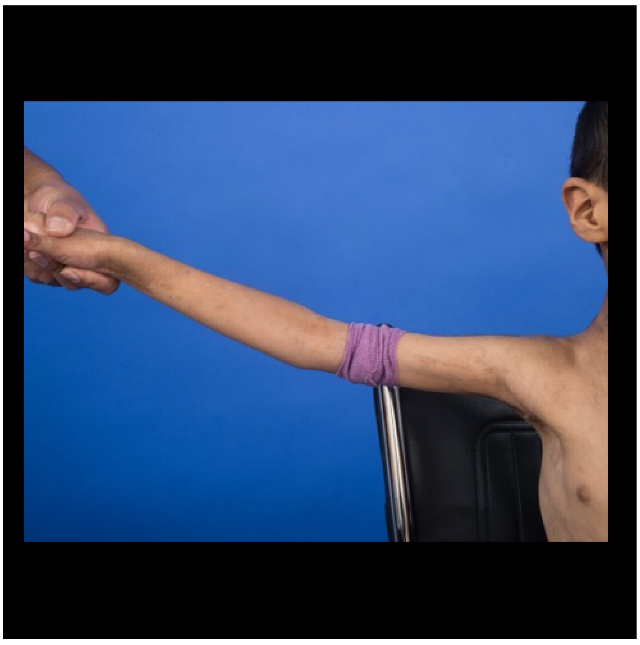

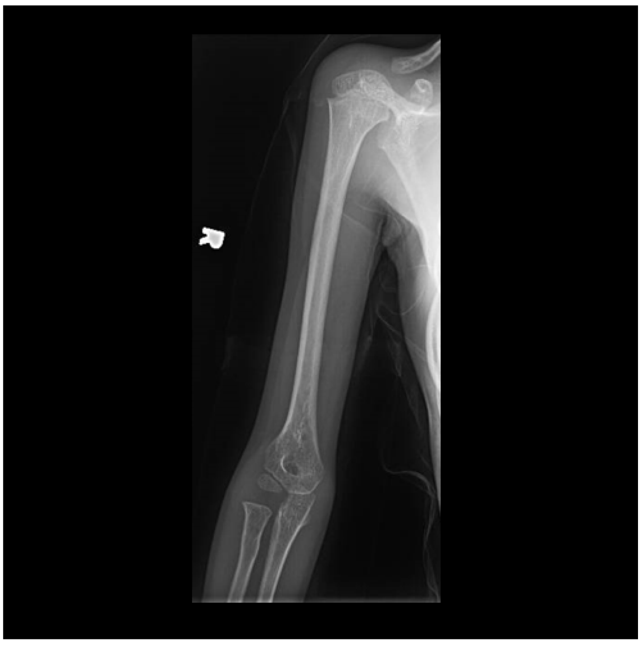

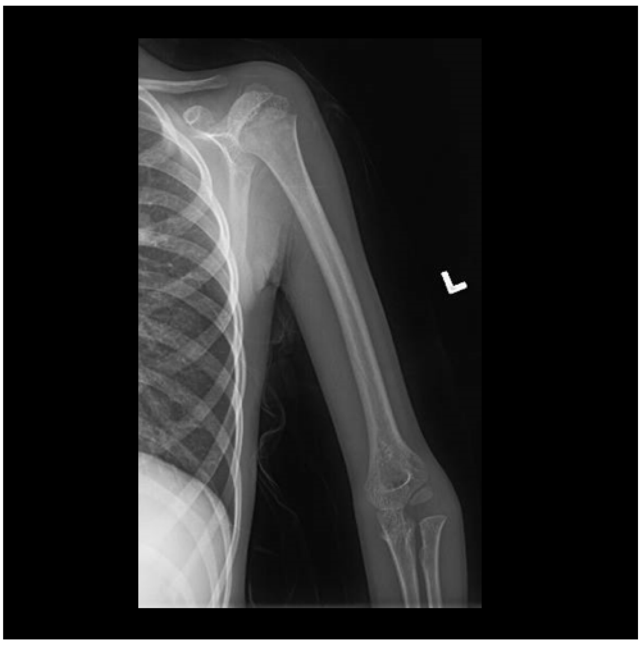

Case 7

Circle | Square

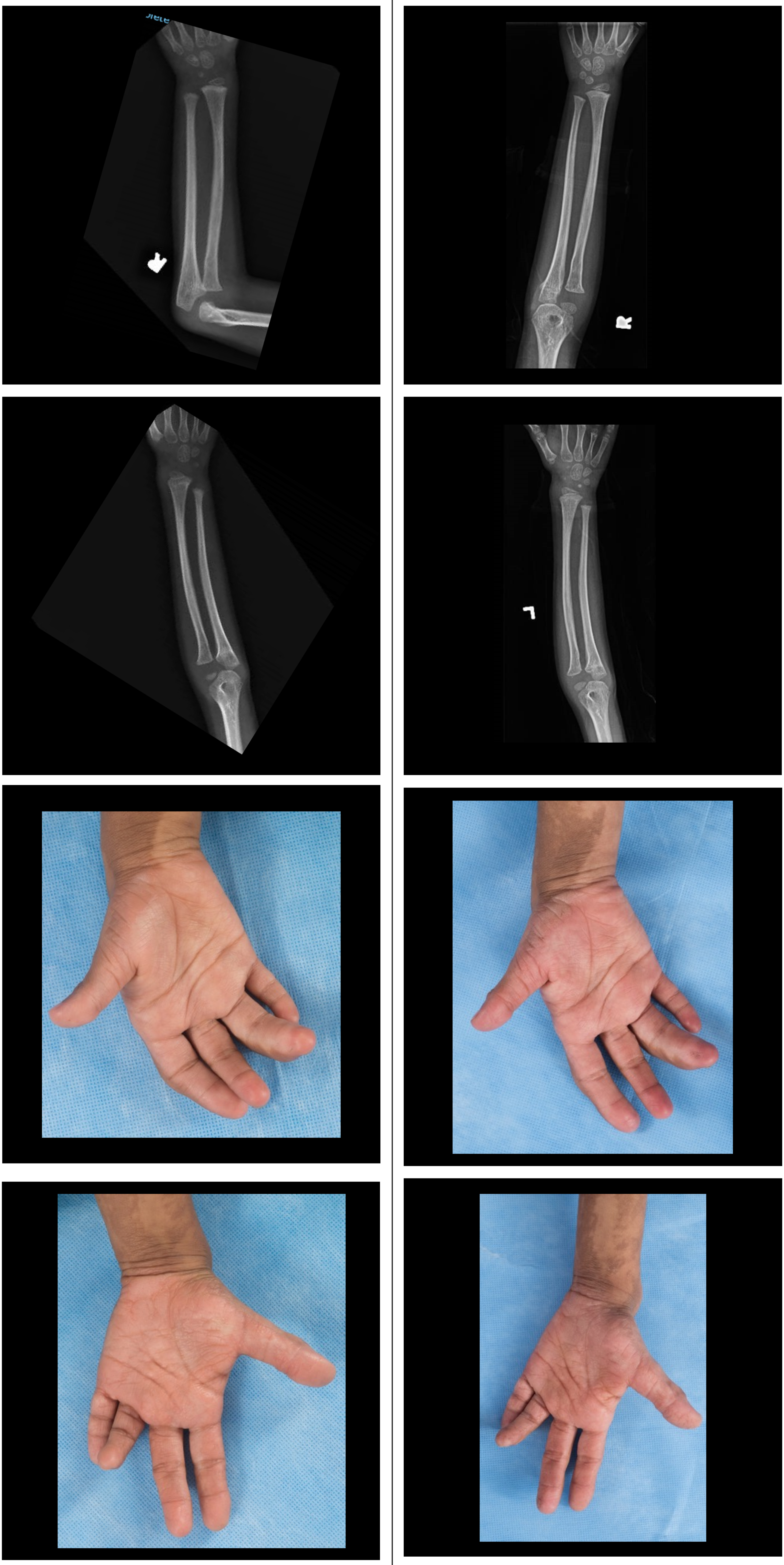

Case 7

Circle

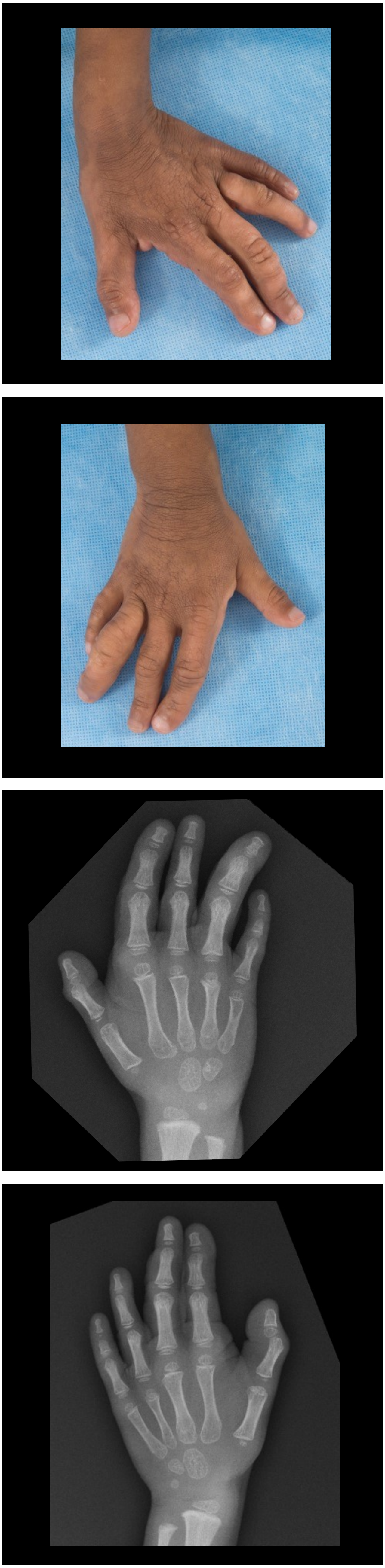

Square

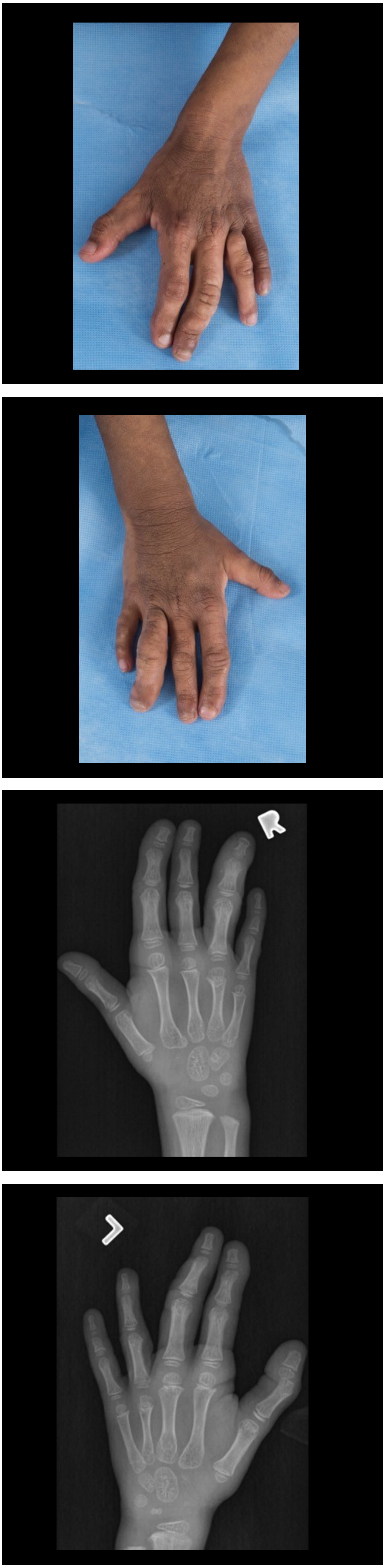

Case 7

Circle

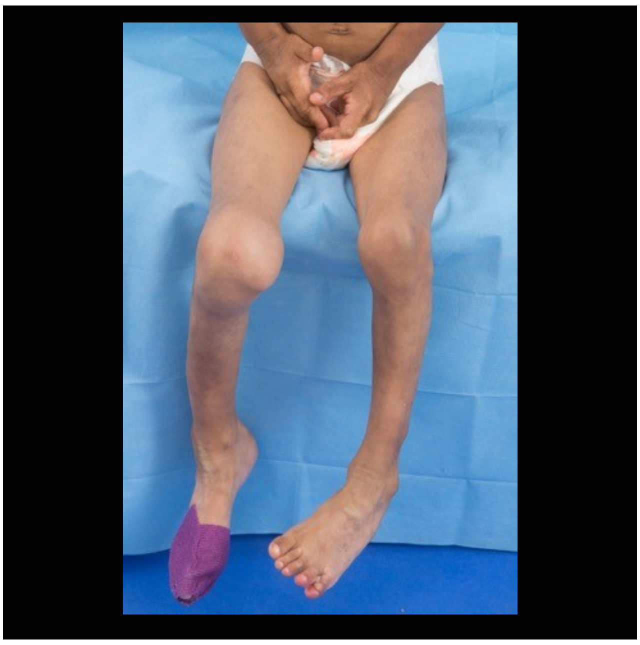

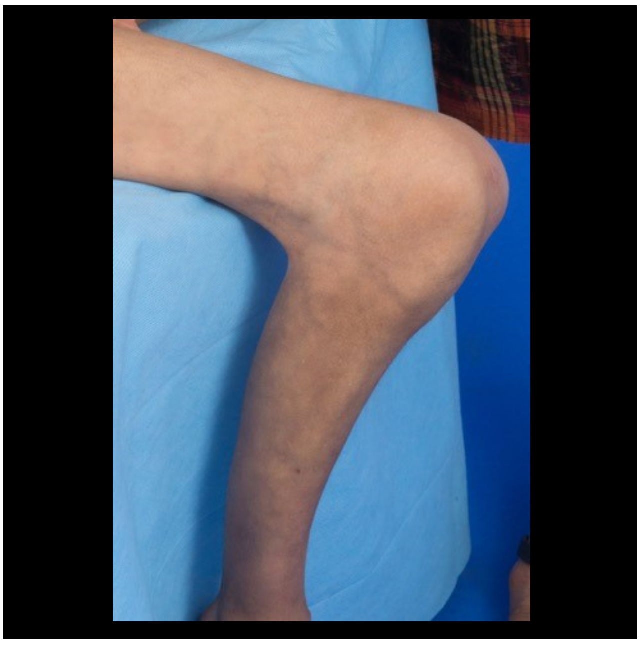

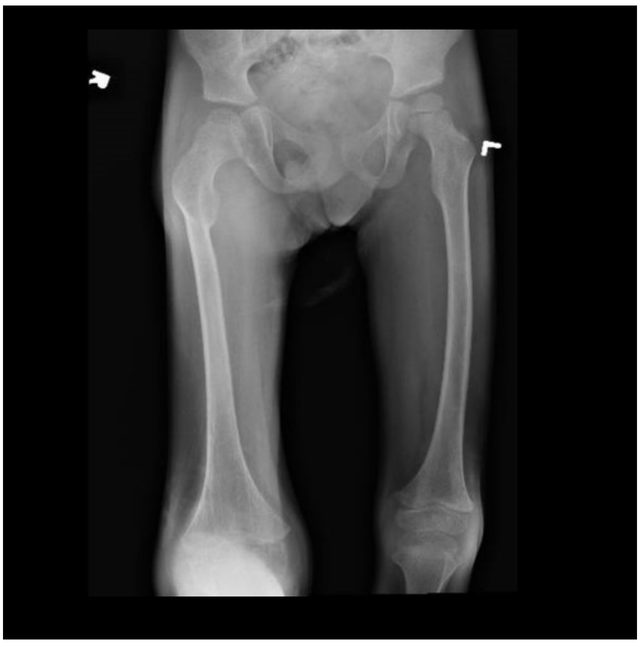

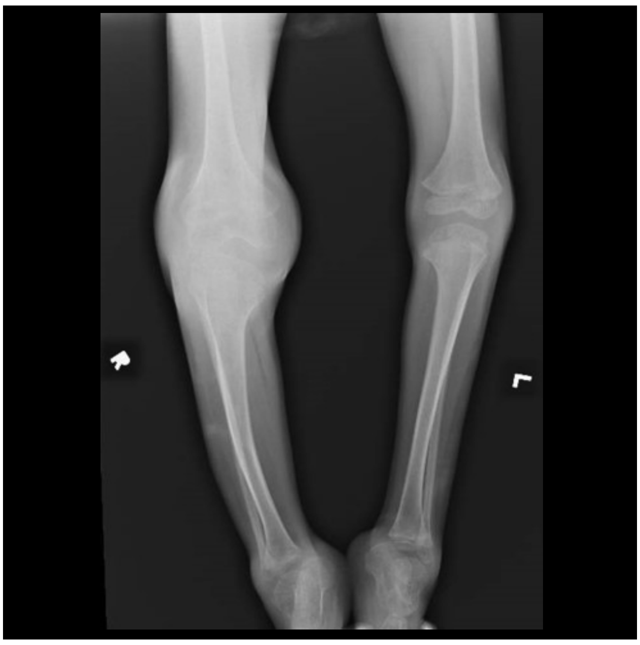

Square

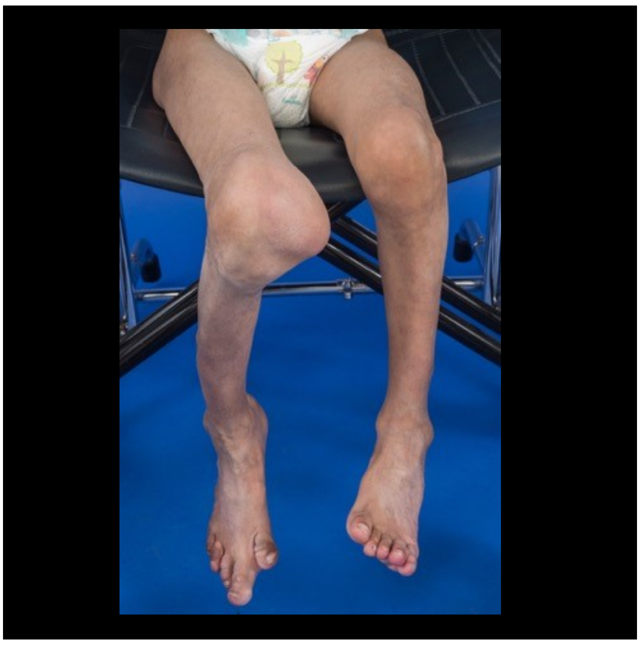

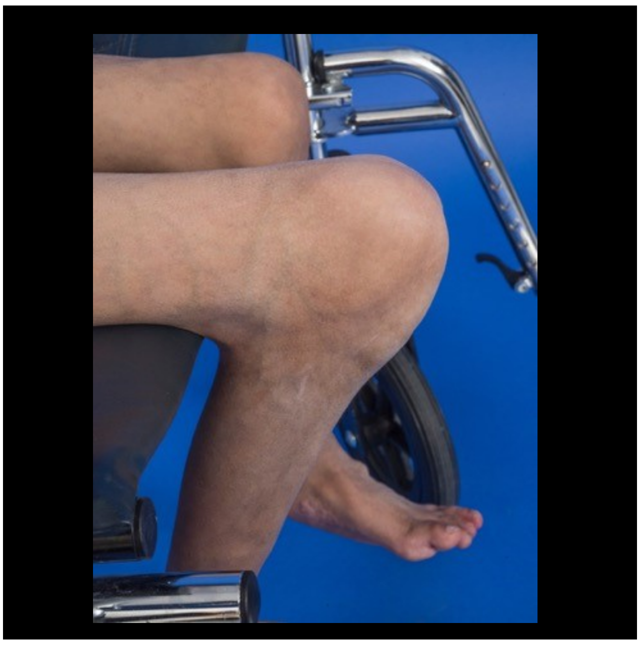

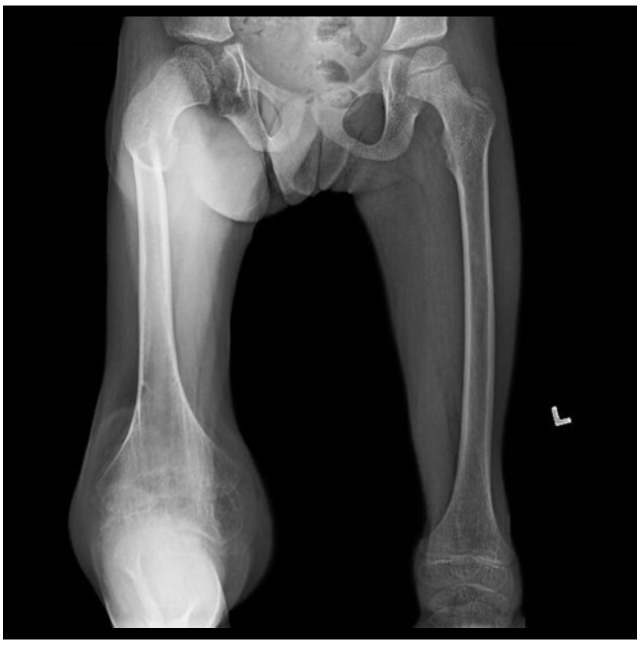

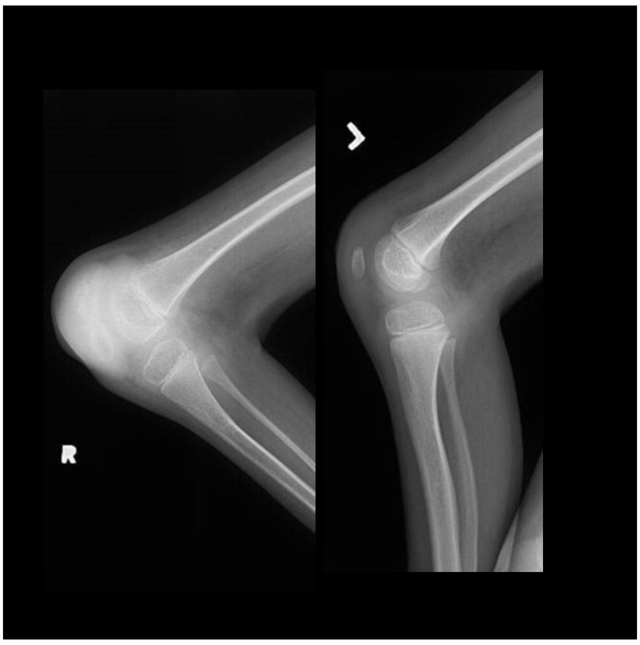

Case 7

Circle | Square

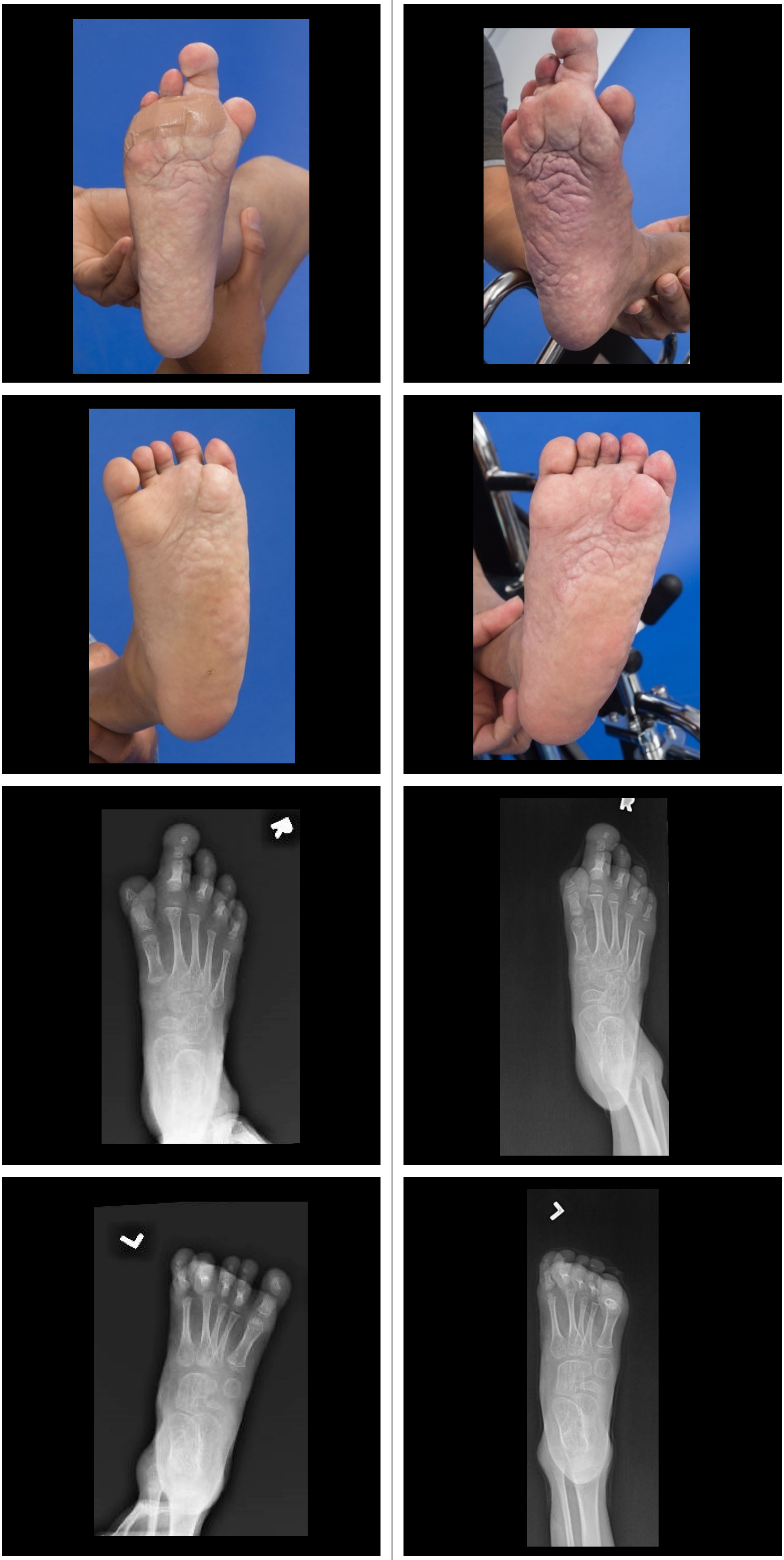

Case 8

Case 8

Circle

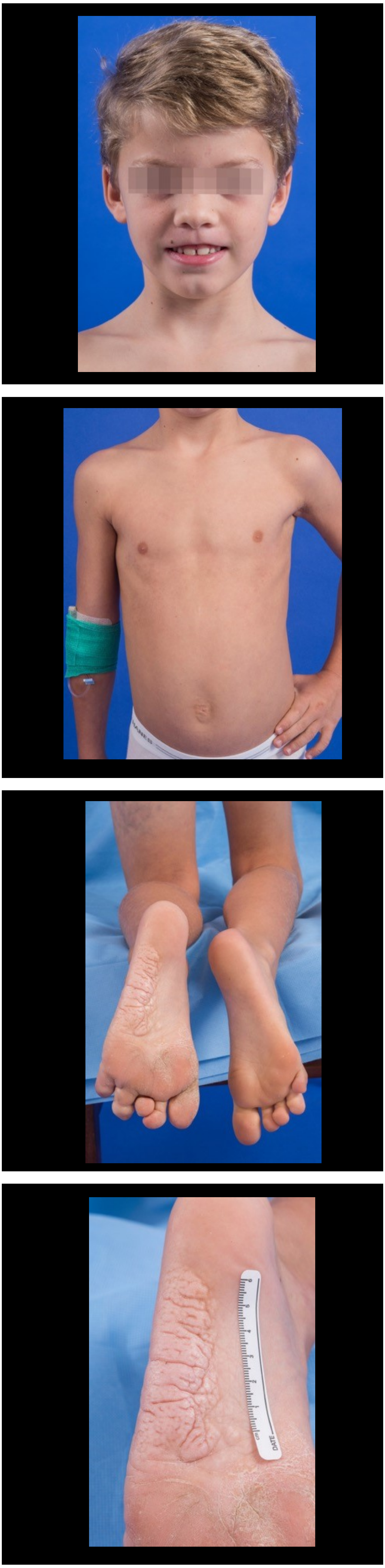

Square

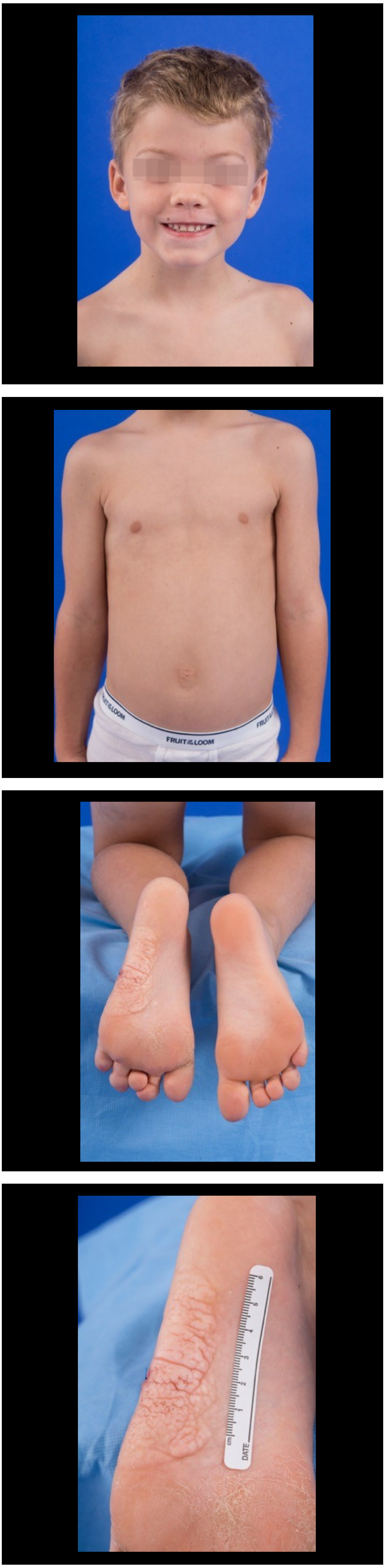

Case 8

Circle

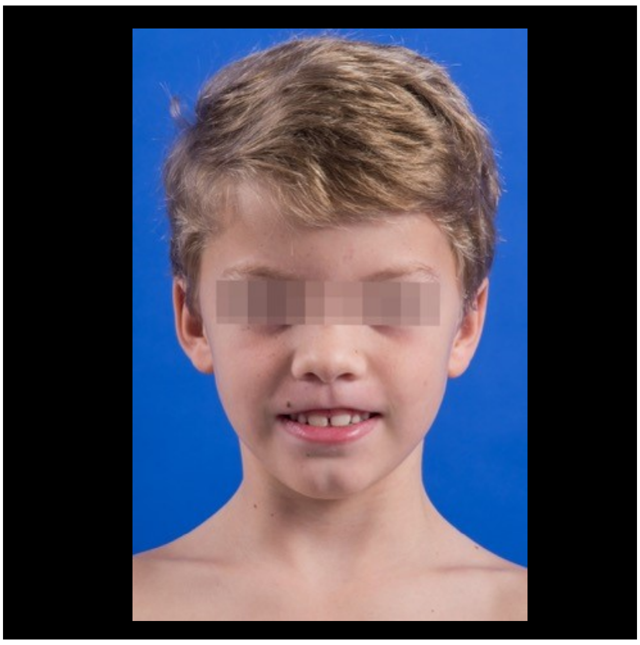

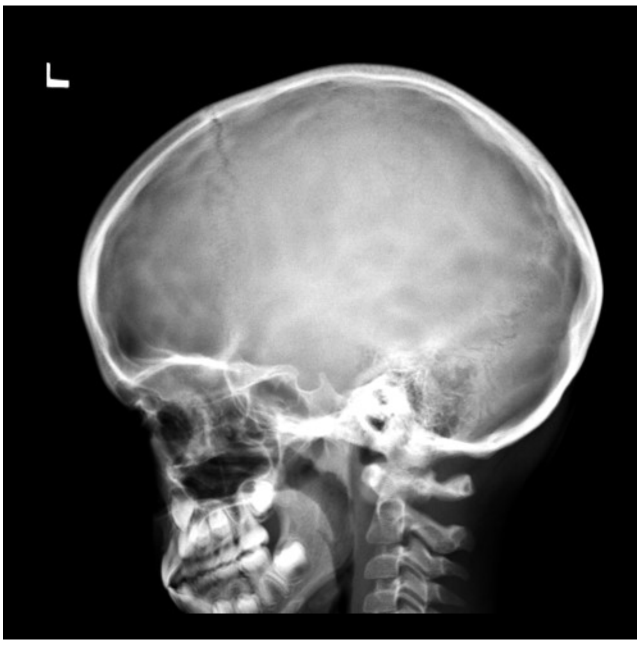

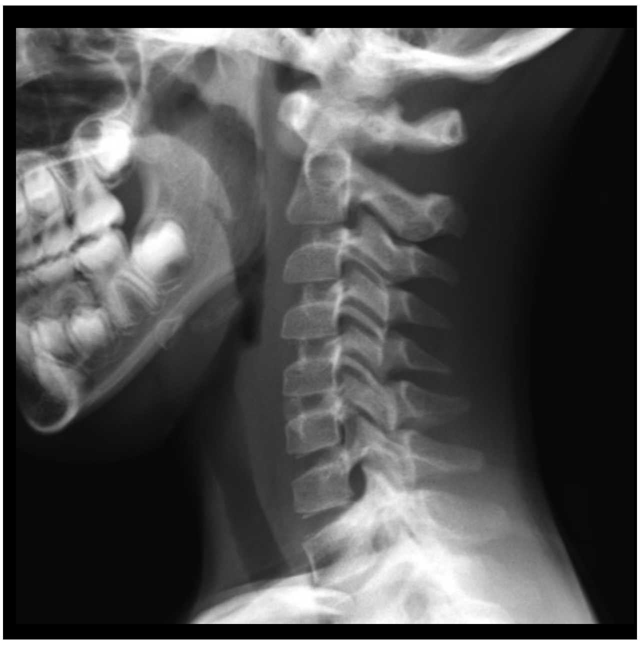

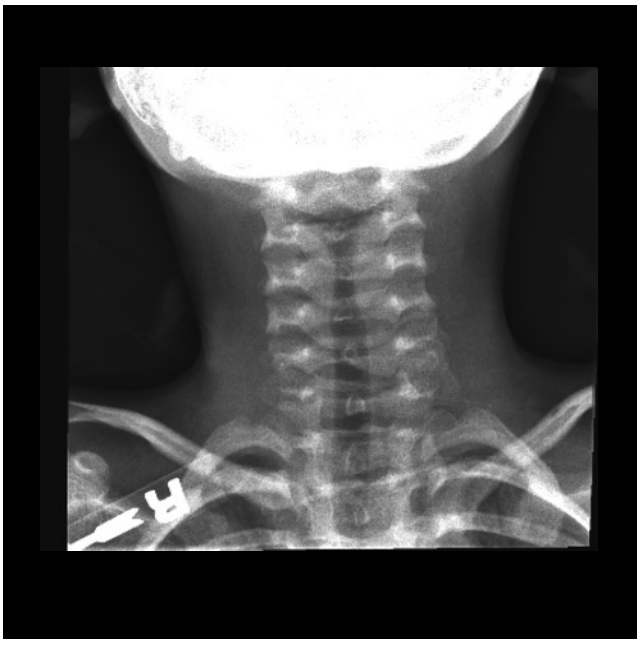

Square

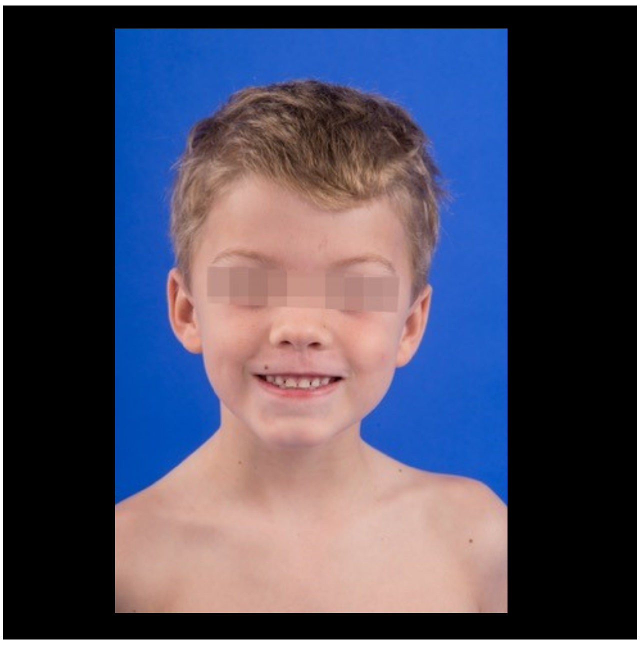

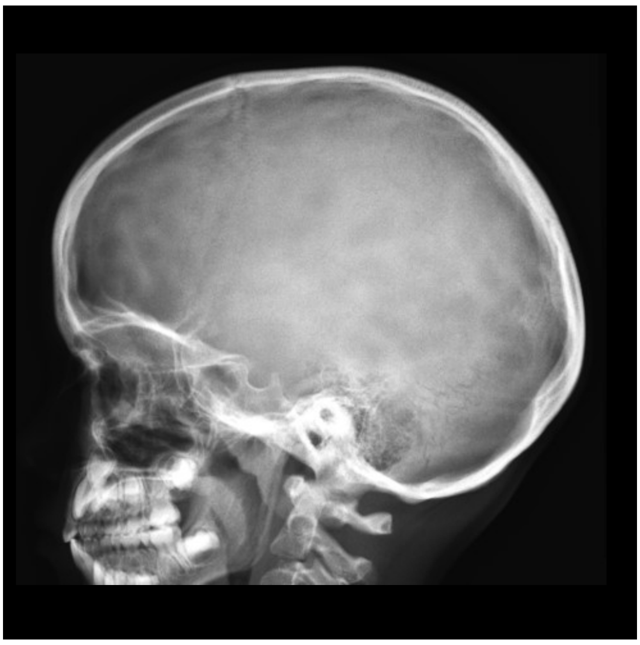

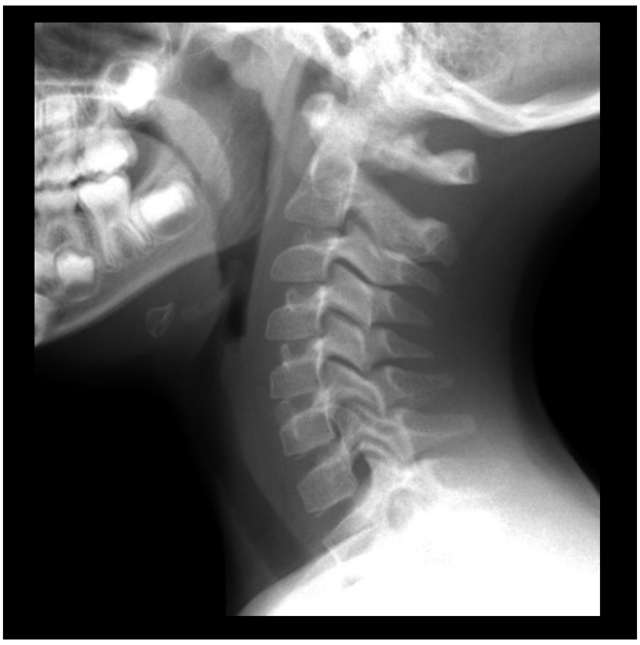

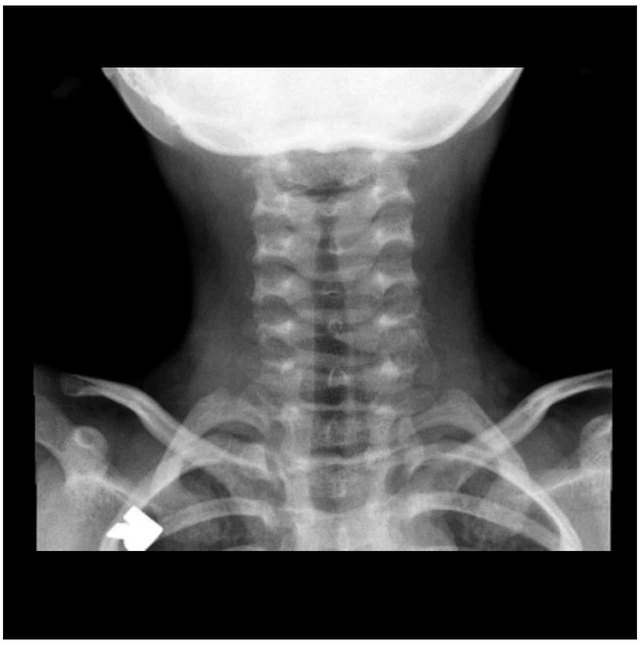

Case 8

Circle

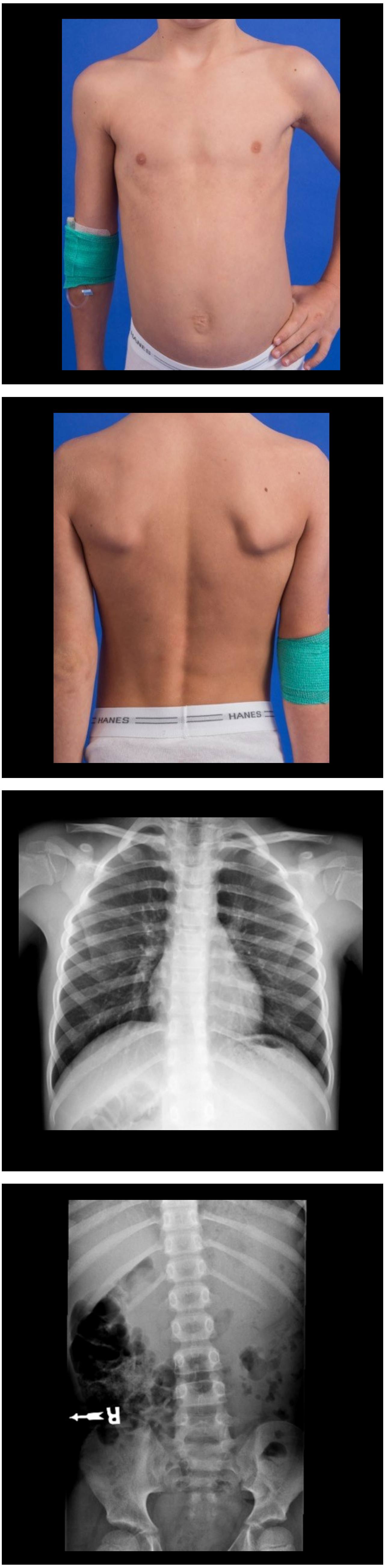

Square

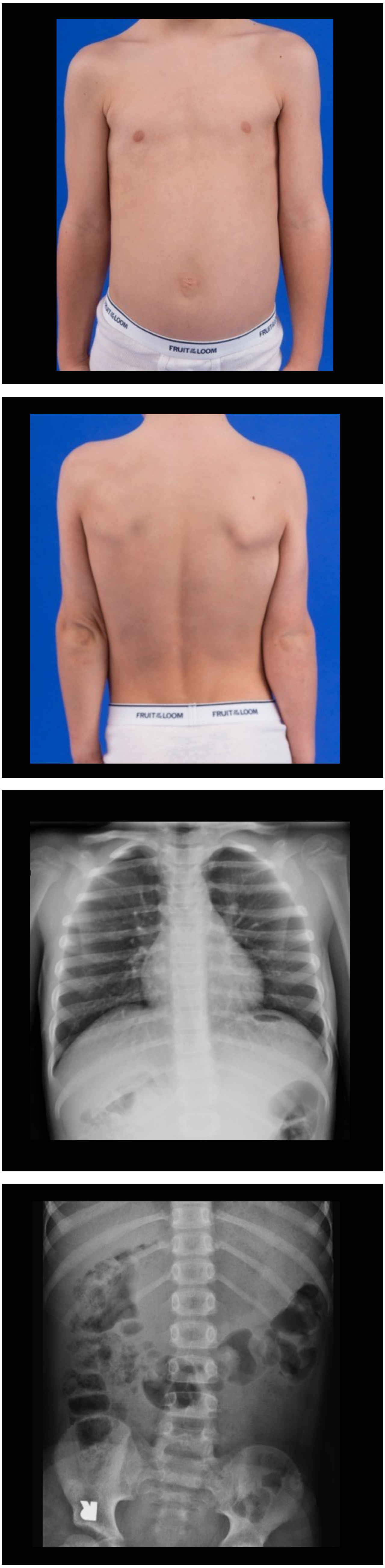

Case 8

Circle

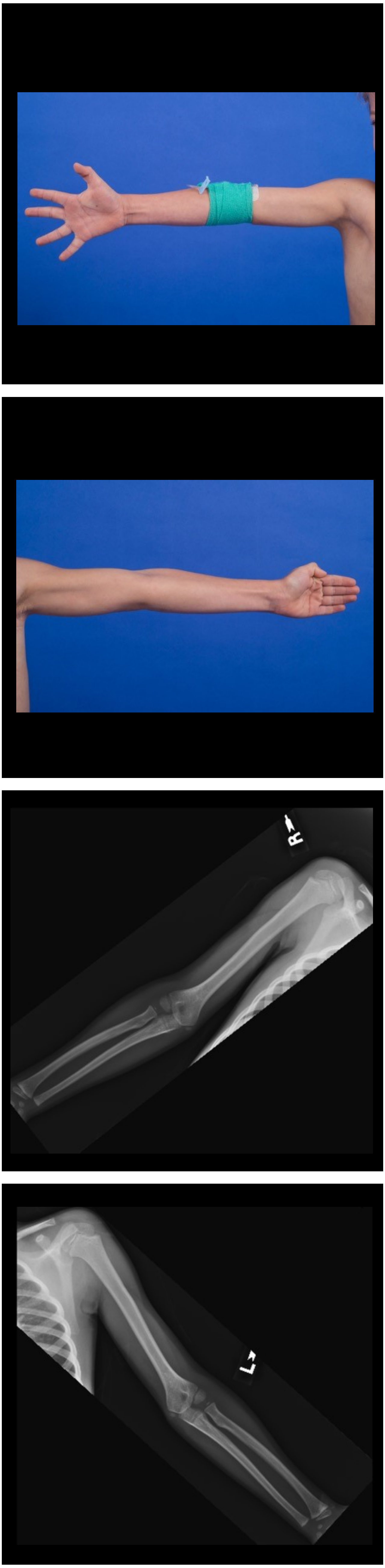

Square

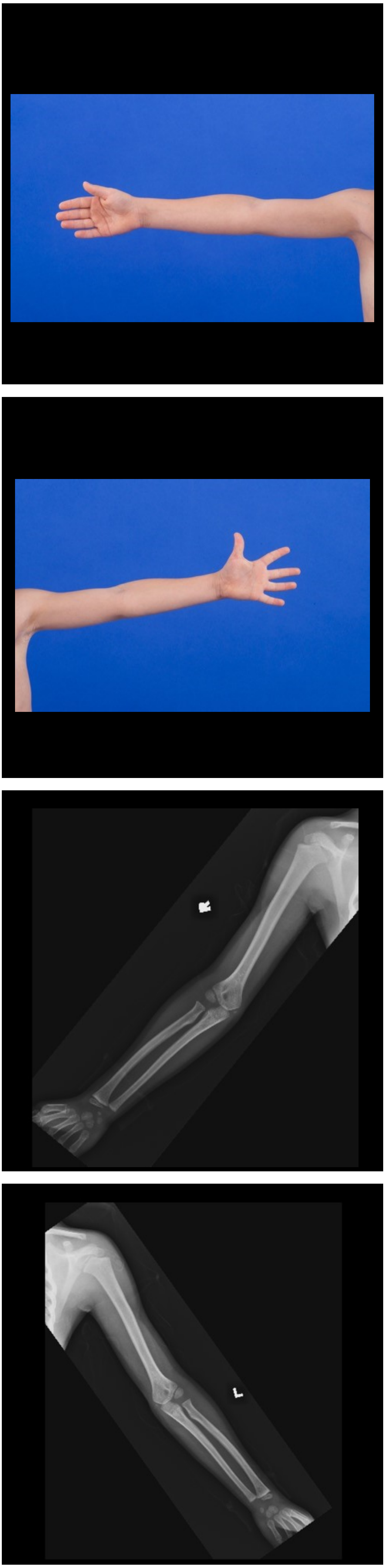

Case 8

Circle

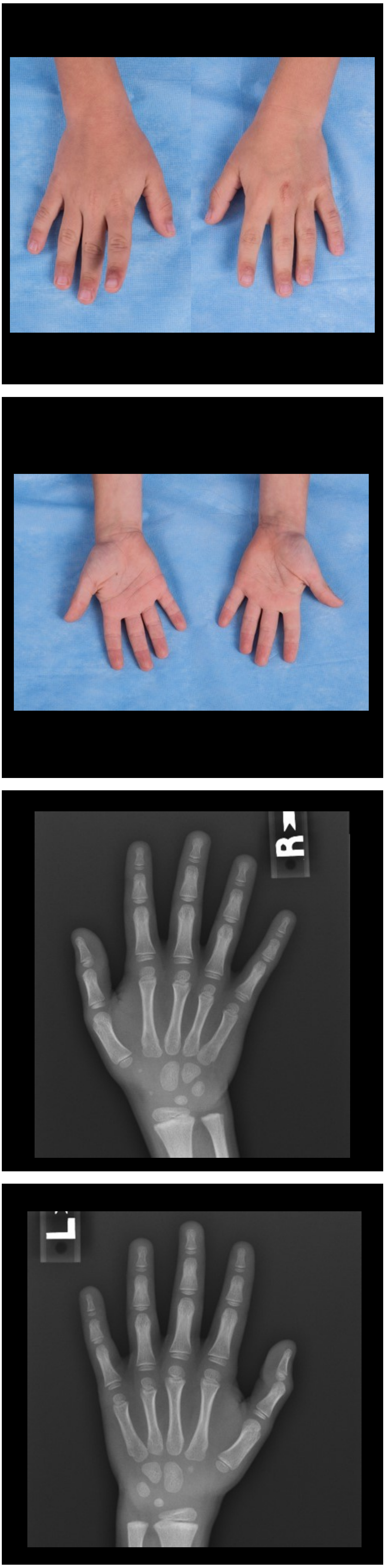

Square

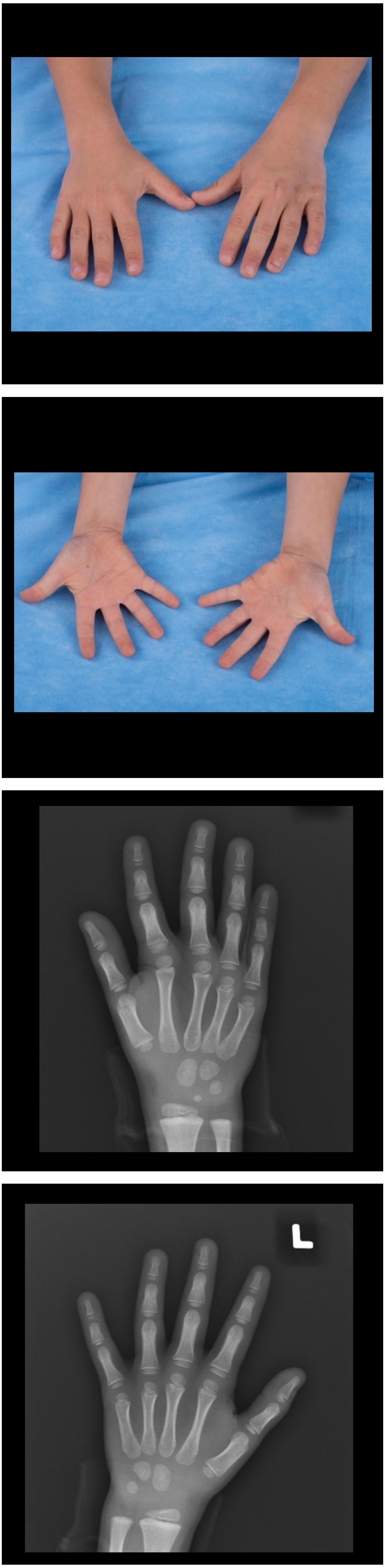

Case 8

Circle

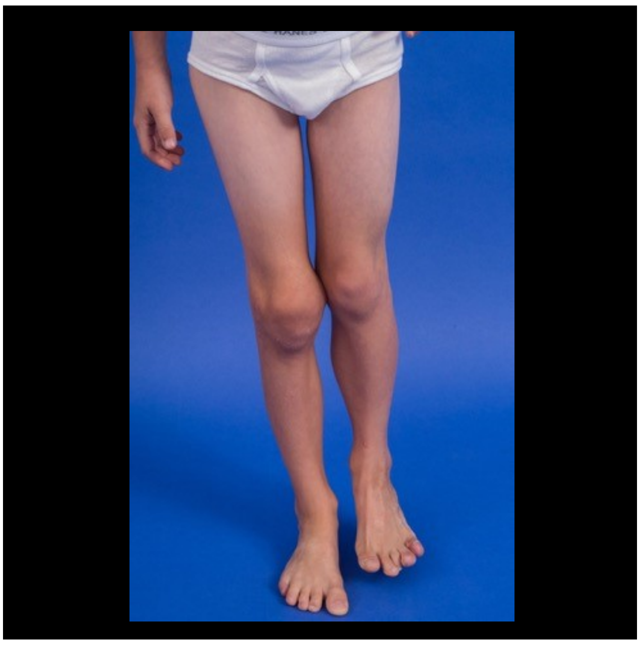

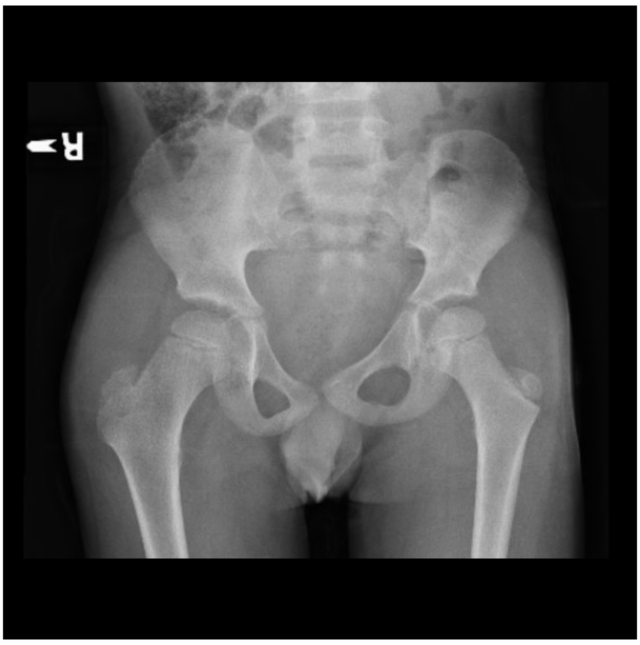

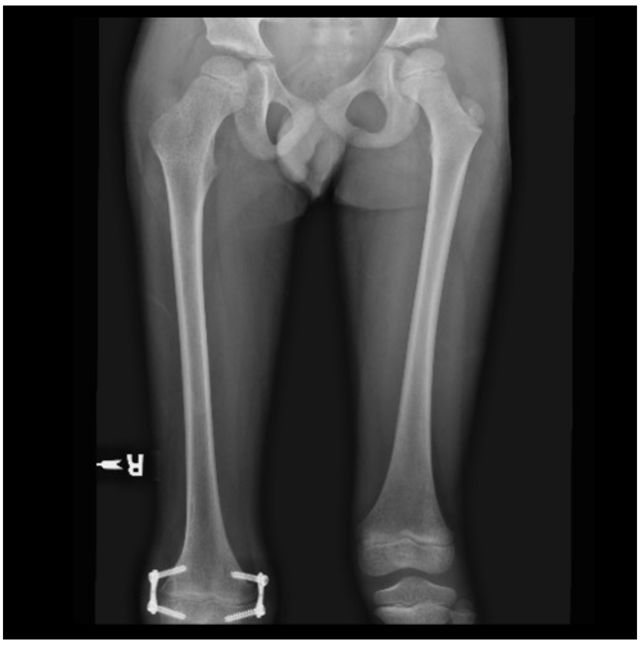

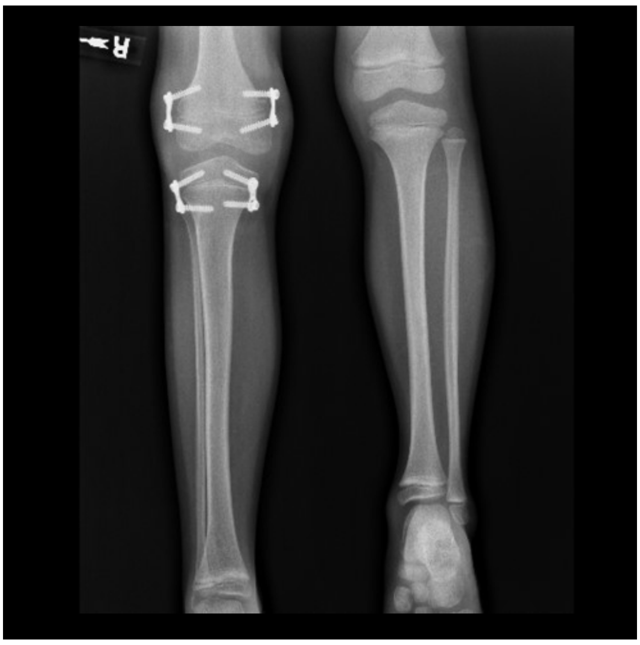

Square

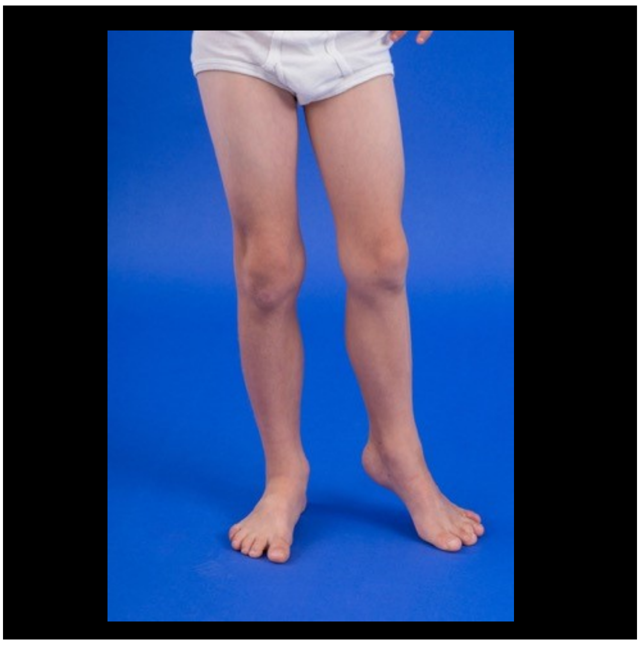

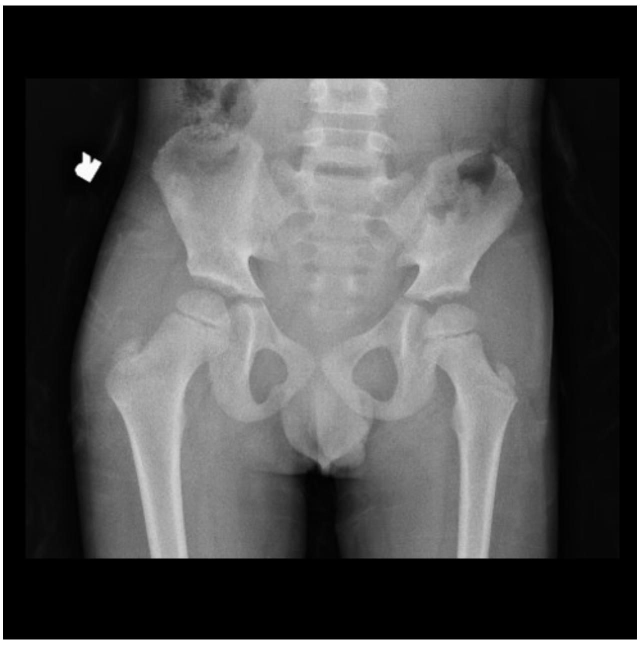

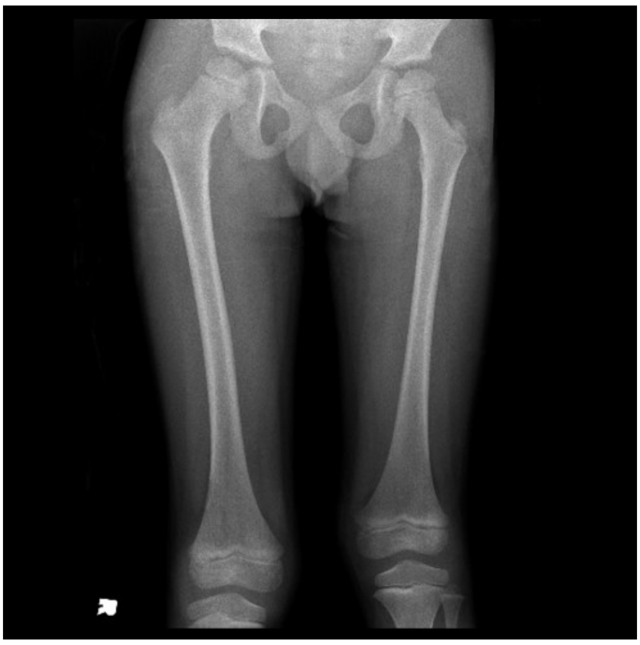

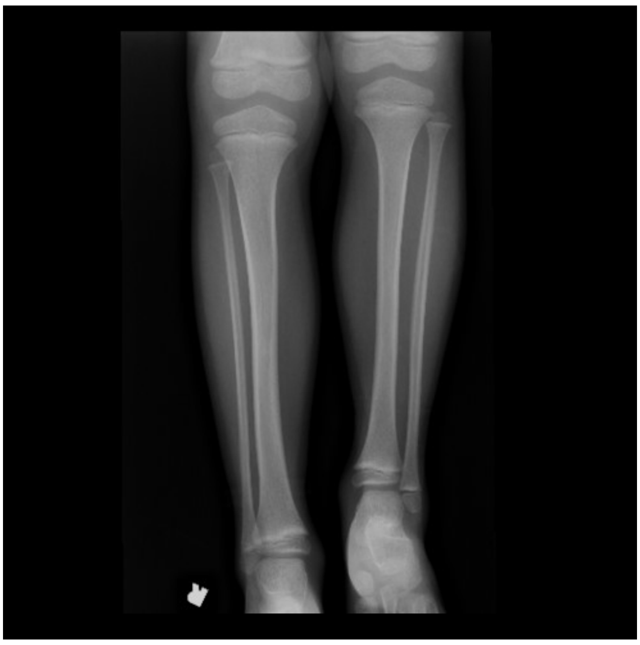

Case 8

Circle

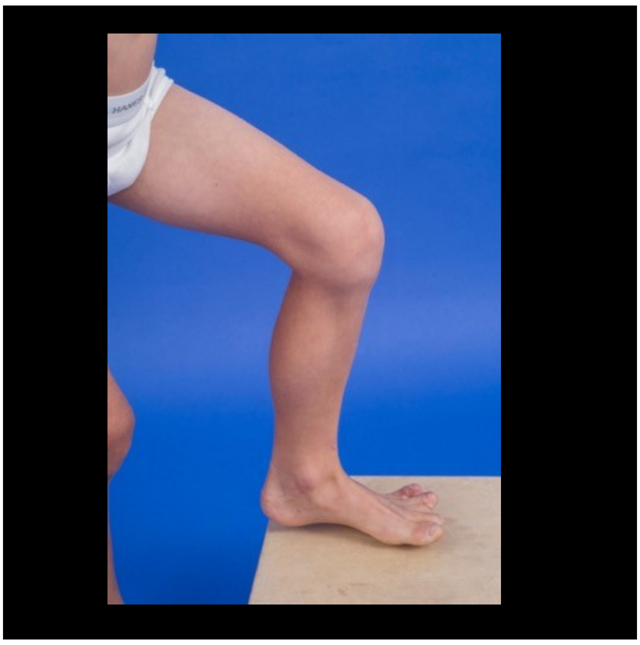

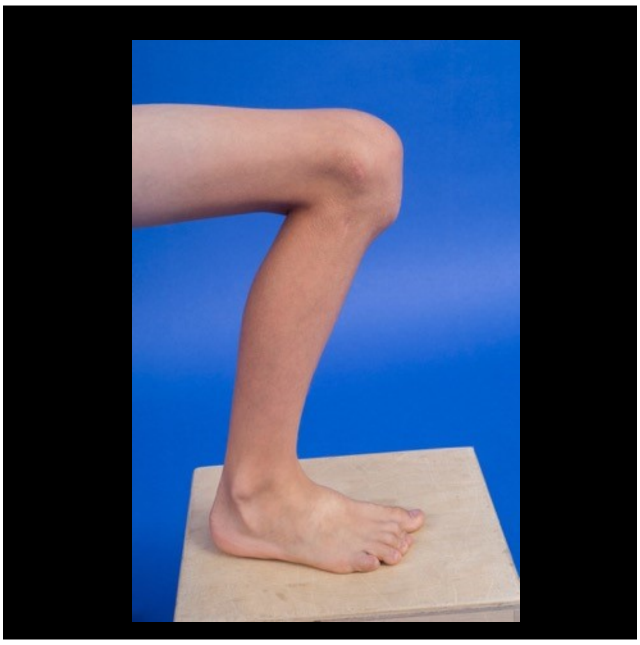

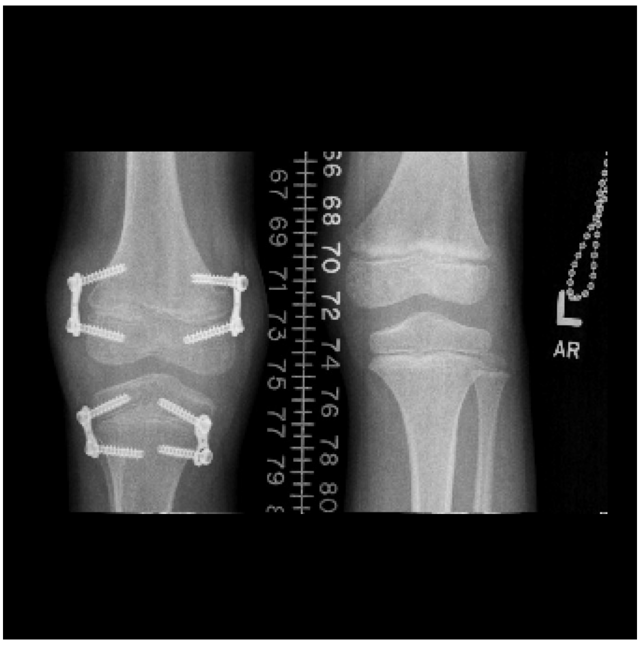

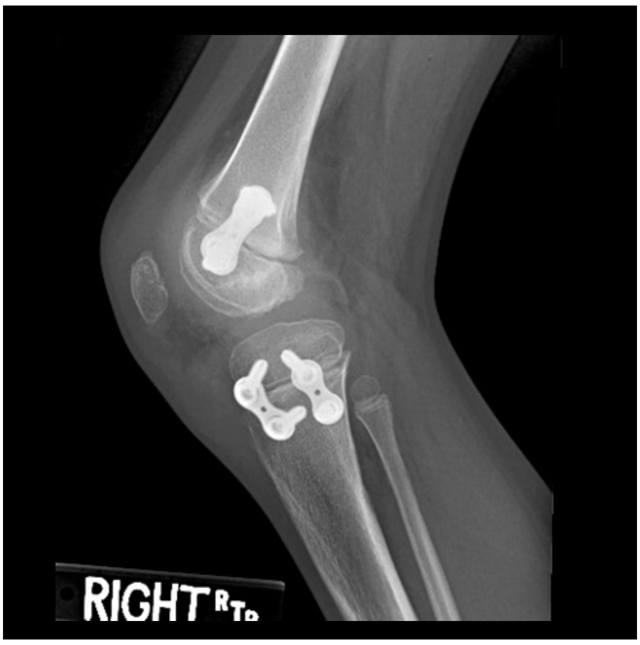

Square

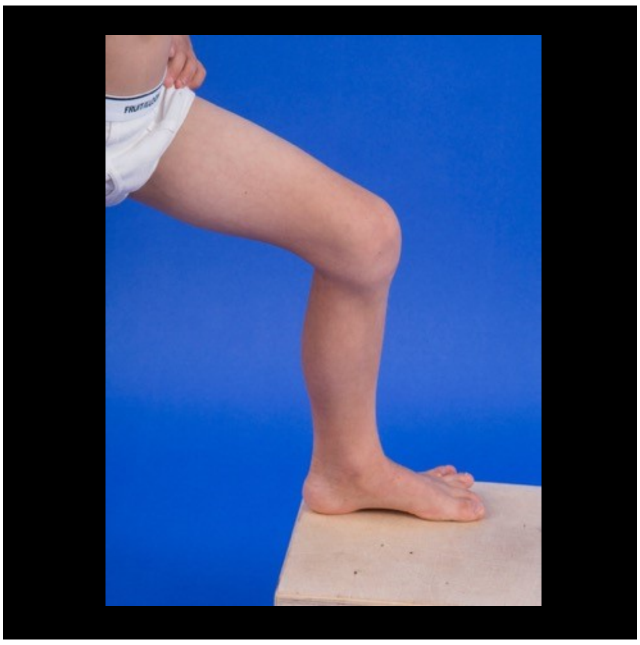

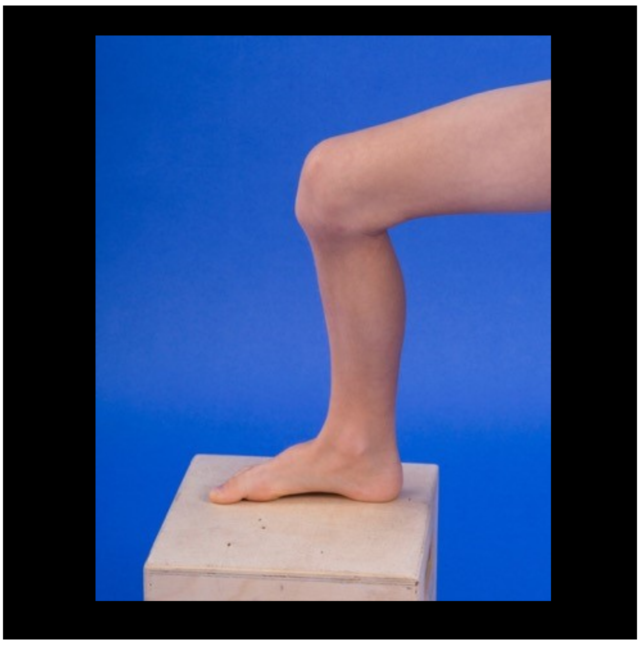

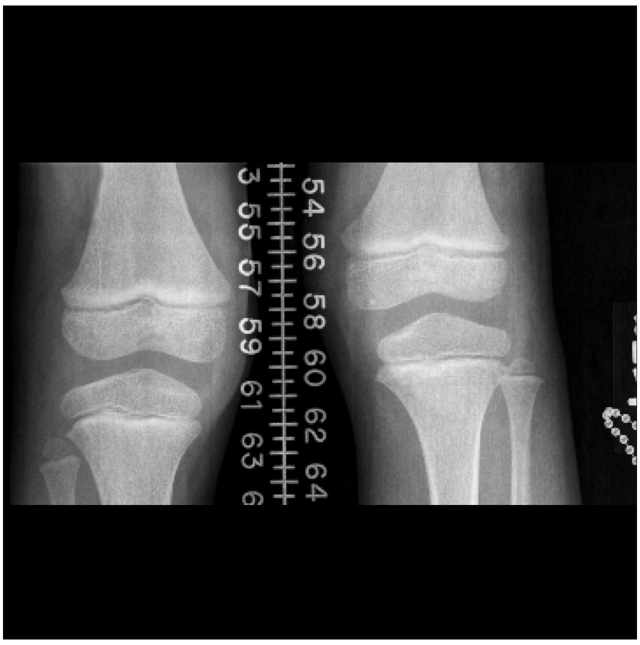

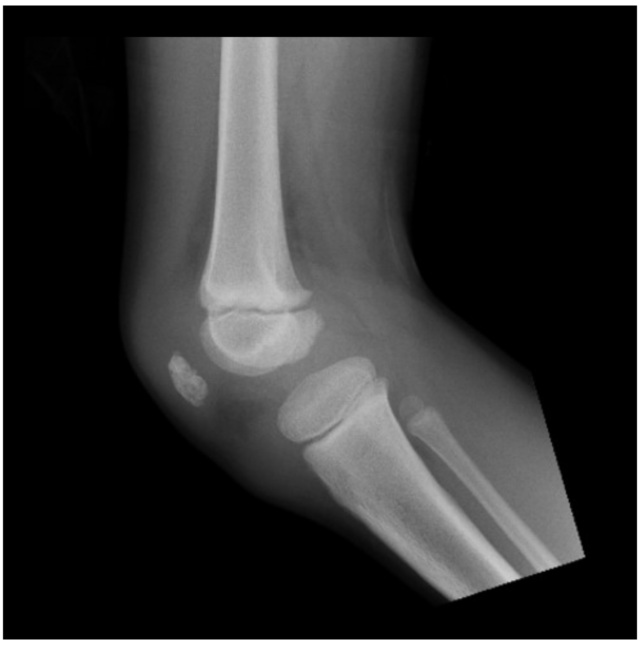

Case 8

Circle

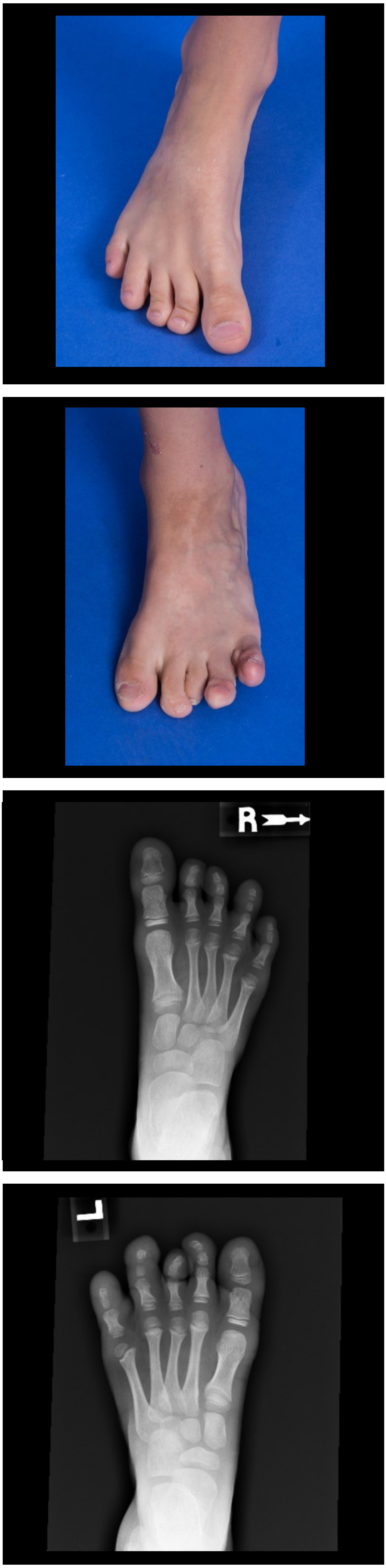

Square

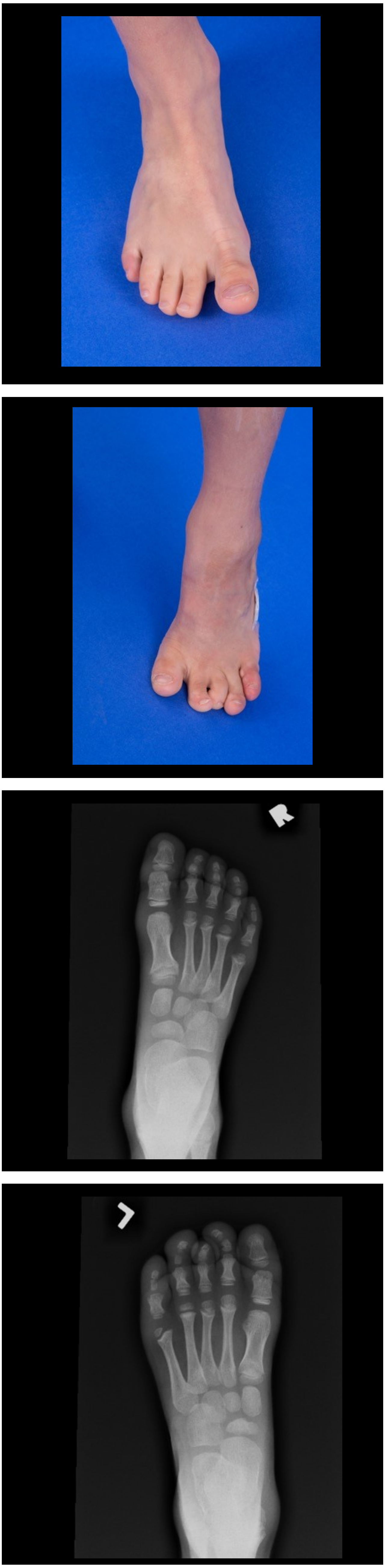

Supplement: Supplementary file 1 — Additional file 1: Fig. S1. Cases reviewed by raters. [file 13023_2022_2325_MOESM1_ESM.pdf]
